# Comparative analyses and structural insights of new class glutathione transferases in *Cryptosporidium* species

**DOI:** 10.1038/s41598-020-77233-5

**Published:** 2020-11-23

**Authors:** Mbalenhle Sizamile Mfeka, José Martínez-Oyanedel, Wanping Chen, Ikechukwu Achilonu, Khajamohiddin Syed, Thandeka Khoza

**Affiliations:** 1 Department of Biochemistry, School of Life Sciences, University of KwaZulu-Natal (Pietermaritzburg Campus), Scottsville, Pietermaritzburg, KwaZulu-Natal 3209 South Africa; 2 Laboratorio de Biofísica Molecular, Departamento de Bioquímica y Biología Molecular, Facultad de Ciencias Biológicas, Universidad de Concepción, Barrio Universitario S/N, Casilla 160_C, Concepción, Chile; 3 Department of Molecular Microbiology and Genetics, University of Göttingen, 37077 Göttingen, Germany; 4 Protein Structure-Function Research Unit, School of Molecular and Cell Biology, University of the Witwatersrand, Braamfontein, Johannesburg, South Africa; 5 Department of Biochemistry and Microbiology, Faculty of Science and Agriculture, University of Zululand, 1 Main Road Vulindlela, KwaDlangezwa, 3886 South Africa

**Keywords:** Biochemistry, Computational biology and bioinformatics

## Abstract

Cryptosporidiosis, caused by protozoan parasites of the genus *Cryptosporidium*, is estimated to rank as a leading cause in the global burden of neglected zoonotic parasitic diseases. This diarrheal disease is the second leading cause of death in children under 5 years of age. Based on the *C. parvum* transcriptome data, glutathione transferase (GST) has been suggested as a drug target against this pathogen. GSTs are diverse multifunctional proteins involved in cellular defense and detoxification in organisms and help pathogens to alleviate chemical and environmental stress. In this study, we performed genome-wide data mining, identification, classification and in silico structural analysis of GSTs in fifteen *Cryptosporidium* species. The study revealed the presence three GSTs in each of the *Cryptosporidium* species analyzed in the study. Based on the percentage identity and comprehensive comparative phylogenetic analysis, we assigned *Cryptosporidium* species GSTs to three new GST classes, named Vega (ϑ), Gamma (γ) and Psi (ψ). The study also revealed an atypical thioredoxin-like fold in the *C. parvum* GST1 of the Vega class, whereas *C. parvum* GST2 of the Gamma class and *C. melagridis* GST3 of the Psi class has a typical thioredoxin-like fold in the N-terminal region. This study reports the first comparative analysis of GSTs in *Cryptosporidium* species.

## Introduction

Cryptosporidiosis is a zoonotic parasitic disease that is caused by *Cryptosporidium* spp.^1–3^. This disease is estimated to be among the highest ranking causes in the global burdens of zoonotic parasitic disease, with an estimate of 8.37 million disability-adjusted life years^2,4^. Recently, large population studies revealed that cryptosporidiosis has become a fast-growing burden to children under the age of 5 years^5,6^. Moreover, the Global Enteric Multicenter Study (GEMS) showed that *Cryptosporidium* is significantly associated with diarrheal disease among children < 24 months of age in sub-Saharan Africa and South Asia^5^. Similar studies also found *Cryptosporidium* to be the second leading cause of moderate to severe diarrhea in infants after Rotavirus^6^*.* It is interesting to note that vaccines/treatment are already available or fast being developed for three of four diarrheal pathogens (*Rotavirus, Shigella* and heat-stable, enterotoxigenic *Escherichia coli*), the exception being *Cryptosporidium,* highlighting the need to address this disease^7^. Despite the global burden of cryptosporidiosis, to date nitazoxanide (NTZ) is the only treatment available for this disease. NTZ only appears to be effective in patients with a good immune response, whilst having limited efficacy in malnourished children and ineffective in immunocompromised people^8–10^. The lack of effective treatment for cryptosporidiosis, coupled with the fact that it is now considered the most common cause of human parasitic diarrhea in the world, highlights the need for more research on *Cryptosporidium* to identify new drug targets and thus develop new drugs^11^.

Cryptosporidiosis is typically characterized by nausea, profuse watery diarrhea, abdominal cramps, vomiting and low-grade fever, which manifest after 14 days and last up to 2.5 months in immune-competent patients^12,13^. These symptoms are usually self-limiting in immune-competent patients; however, in immunocompromised hosts they can be devastating, with the disease manifesting as life-threatening and often becoming extraintestinal^13^. The gastrointestinal infection can spread to other sites, such as the gall bladder, biliary tract, pancreas and pulmonary system. Cryptosporidiosis can be contracted through the fecal–oral route, through contact with infected animals or humans or contaminated food or water^13^.

Of the *Cryptosporidium* species that exist, *C. hominis* and *C. parvum* are responsible for the highest level of clinically relevant infections worldwide^3^. The remaining species have mild zoonotic properties causing moderate-to-severe diarrhea in humans^3^. *Cryptosporidium* species are reported to have an efficient defense mechanism that allows it to cope with a wide range of environmental stresses such as changes in temperature, drugs, free radicals, as well as the host’s immune responses at various life stages^12^. Genome analysis of *C. parvum* revealed that it contains various defense proteins such as glutathione transferase (GST), glutathione peroxidase and superoxide dismutase, which are known for detoxification, signal modulation and aromatic amino acid catabolism^14^. The existence of these enzymes may provide *C. parvum* with the abilities to maintain its parasitic lifecycle, enabling it to survive and persist in its host.

Among the above-mentioned enzymes, GST is found to be expressed in all stages of the *C. parvum* parasite’s life cycle^15^, thus making it a promising therapeutic target^16^. GSTs have been studied as drug targets against infectious agents and metabolic disorders^17–19^. GSTs are a diverse group of multifunctional proteins that are distributed ubiquitously in eukaryotes and prokaryotes^20,21^. These enzymes play an important role in cellular defense and detoxification^20,22,23^. They catalyze the nucleophilic conjugation of the reduced tripeptide glutathione (GSH) thiol group to the electrophilic substrates to convert them to less harmful, more soluble compounds. Based on the location, the GST superfamily is divided into three sub-families namely, soluble or cytosolic GSTs, mitochondrial GSTs and membrane-associated proteins involved in eicosanoid and gluthatione metabolism (MAPEG) with the cytosolic GSTs being the most characterized (Table S1). The GSTs are generally divided into classes based on amino acid sequence similarity, with GSTs within each class sharing similar immunological cross-reactivity and specificity towards the electrophilic substrate and sensitivity to inhibitors^20,24,25^. GSTs within each class typically share as little as 60% amino acid sequence identity; however, some classes can share from as little as 40%^20,23,26–28^. It is generally accepted that the assignment of different GSTs to specific classes must fall within these limits, with sequences sharing less than 25–30% designated to their own class^20,23,26–28^. Information on different GST classes found in organisms, their cellular localization and functions are listed in Table S1.

Typical GSTs are dimeric in structure and each monomer is divided into two domains^20,23^. The N-terminal domain of conical GSTs assumes a topology resembling the thioredoxin fold with a βαβ-ββα motif. This domain also houses an important conserved region of the active site where a catalytically active Tyr, Ser or Cys is found to interact with the GSH thiol group. The C-terminal domain of typical GSTs is all helical and connected by a short linker sequence called the *cis*-Pro loop with a highly-conserved proline residue in *cis* conformation^23^. The active site is comprised of the glutathione binding site (G-site) and the hydrophobic substrate binding site (H-site), located in the N- terminal and C-terminal domain respectively. The G-site exclusively binds glutathione and is highly conserved, whilst the H-site accepts more variability so to accommodate an extensive range of toxic electrophilic substances^20,23^.

Despite the importance of GSTs, especially as a potential drug target against *Cryptosporidium*^16^, to the best of our knowledge, no literature is available to date on *Cryptosporidium* GSTs with regards to their distribution, the GST classes and structural information. Thus, this study is aimed at addressing this research gap. In this study, genome data mining, identification, phylogenetic and structural analysis of GSTs in fifteen *Cryptosporidium* species has been carried out.

## Methods

### Species and database

*Cryptosporidium* species genomes that are available for public use at the *Cryptosporidium* database or CryptoDB^29^ (https://cryptodb.org/cryptodb/app; release 48 beta, 27 August 2020; accessed on 14 September 2020) and at National Center for Biotechnology information (NCBI)^30^ (https://www.ncbi.nlm.nih.gov/datasets/genomes/?txid=5806; accessed on 14 September 2020) were used in the study. The *Cryptosporidium* pathogens examined in this study include ones from both humans and other mammals (Table 1).

**Table 1:** *Cryptosporidium* species used in the study and their major host specificity.

Species and isolates	Host range	Reference(s)
*Cryptosporidium andersoni* isolate 30847	Cattle, sheep, bactrian camel, gerbil	^31^
*Cryptosporidium hominis* isolate TU502_2012	Humans, monkeys, macaque, kangaroo, calf and piglets	^32,33^
*Cryptosporidium hominis* isolate 30976	Humans, monkeys, macaque and kangaroo	^33,34^
*Cryptosporidium hominis* TU502	Humans, monkeys macaque, kangaroo, calf and piglets	^33,35^
*Cryptosporidium hominis* UdeA01	Humans, monkeys, macaque, kangaroo	^36,37^
*Cryptosporidium meleagridis* strain UKMEL1	Human, turkey, chicken, bobwhite quail, dog	^32,37^
*Cryptosporidium parvum* Iowa II	Humans, cattle, sheep, pigs, deer and mice	^14,37–39^
*Cryptosporidium tyzzeri* isolate UGA55	Domestic mice	^40^
*Cryptosporidium ubiquitum* isolate 39726	Deer, sheep, goat, squirrel, mice and beavers	^31,41^
*Cryptosporidium muris* RN66	Mice and cats	^42,43^
*Cryptosporidium baileyi* strain TAMU-09Q1	Chickens and black-headed full, quails, ostriches and ducks	^37,44,45^
*Cryptosporidium viatorum* isolate UKVIA1	Humans and rats	^46,47^
*Cryptosporidium* sp*. chipmunk* LX-2015	Mice, squirrels, chipmunks	^41,48,49^
*Cryptosporidium ryanae* isolate 45019	Cattle	^50^
*Cryptosporidium bovis* isolate 42482	Sheep and cattle	^51,52^

### Genome data mining, identification and classification of GSTs

*Cryptosporidium* species genomes available at CryptoDB^29^ were mined for GSTs. Two different methods followed for GST mining. First, the genomes of *Cryptosporidium* species were mined using the term “glutathione transferase”. Second, the species genomes were blasted with GST proteins from *Homo sapiens* (protein ID: P08263)^53^ and *C. parvum* Iowa II (protein ID: EAK89476.1)^14,38^. The BLASTP mined proteins revealed a range of apicomplexan species which were filtered out to show only *Cryptosporidium* species. The hit proteins were then collected and subjected to protein family analysis using the Pfam^54^ and InterPro^55^ programs. The results were analyzed and the hit proteins that were classified as GST by Pfam (PF14497, PF13417 and, PF17172)^54^ and InterPro (IPR036282, IPR004045 and IPR010987)^55^ were selected.

For the collection of more hits, *Cryptosporidium* species genomes available at NCBI database^30^ was blasted with two GST proteins from *C. andersoni* 30847 (cand_012830 & cand_023790) and from *C. meleagridis* UKMEL1 (CmeUKMEL1_05845) that were collected from CryptoDB^29^. The hit proteins were screened for GSTs following the method described above.

A final total count was presented by deleting the duplicated GSTs. The selected GSTs were then grouped into different classes or groups based on their percentage identity, following the conventional criterion of less than 25–30% identity being a new class^20,23,26–28^.

### Analysis of homology

The percentage identity between GSTs was deduced using Clustal Omega^56^. The full-length GSTs were subjected to Clustal analysis which produced the percentage identity amongst each of the proteins as matrix identity results. These results were laid out in an Excel spreadsheet where the results were analyzed to identify the percentage identity between GSTs.

### Collection of different GST classes’ protein sequences

For comparative analysis, GST protein sequences belonging to different GST classes were collected using multiple methods to build a library for phylogenetic analysis. On the European Molecular Biology Laboratory (EMBL) site^57^, GSTs sequences that are placed under the GST superfamily (IPR040079) were retrieved. The GST classes namely CLIC (IPR002946), Alpha (IPR003080), Mu class (IPR003081), Pi (IPR003082), Omega (IPR005442), Zeta (IPR005955) and Sigma (IPR003083) were collected under EMBL. More sequences were obtained through text search using the UniProt protein knowledge base^58^. A specific GST class was searched on the site and the hits obtained were further verified using Pfam^54^ and InterPro^55^ to ensure uniformity with the GSTs collected from the EMBL site^57^. The remaining GSTs that were not in the databases were retrieved from published articles.

The *Cryptosporidium* species GST sequences along with protein sequences of different GST classes used in the phylogenetic analysis are presented in Supplementary Dataset 1.

### Phylogenetic analysis

The GST sequences in supplementary dataset 1 were used to make a phylogenetic tree for inferring their evolutionary relationship. First, all the GST protein sequences were aligned by MAFFT v6.864 embedded on the Trex-online server^59^. Then, the alignment was automatically submitted to the server for inferring the tree with different models and the optimized tree was selected. Finally, the tree was submitted to iTOL for viewing and annotation^60^. Thioredoxin from *Oryctolagus cuniculus* (protein ID: P08628) was used as an outgroup.

For the construction of the phylogenetic tree of the *Cryptosporidium* GST proteins, the protein sequences were aligned using MUSCLE software^61^ embedded in MEGA7^62^. The evolutionary history was inferred by using the maximum likelihood method with 100 bootstrap replication based on the JTT matrix-based model^63^. Evolutionary analyses were conducted in MEGA7.

### Cellular localization and transmembrane helices prediction

Cellular localization of GSTs was predicted using the Bologna Unified Subcellular Component Annotator (BUSCA)^64^. BUSCA is the latest, accurate program available for the prediction of proteins’ subcellular localization; it integrates different computational tools such as identifying signal and transit peptides (DeepSig and TP-pred3), GPI-anchors (PredGPI) and transmembrane domains (ENSEMBLE3.0 and BetAware) with tools for discriminating subcellular localization of both globular and membrane proteins (BaCelLo, MemLociand SChloro)^64^. The outcomes of these different programs were processed and integrated to predict subcellular localization of both eukaryotic and bacterial proteins^64^. Prediction of transmembrane helices in GSTs was done using TMHMM Server v. 2.0^65^. This program is well known for its high degree of accuracy in the prediction of transmembrane helices and discrimination between soluble and membrane proteins.

### Template identification

To construct 3D models of proteins, reference protein structures previously solved by crystallization or Nuclear Magnetic Resonance are needed. These would serve to simulate not only the fold of a protein but also a full atom model to build. These proteins are referred to as templates. Either single or multiple templates can be used in constructing the 3D model of a protein^66^. In this study, three different web servers, namely NCBI BLAST (v2.10.1)^67^, i-TASSER (v5.1)^68^ and PHYRE (v2.0)^69^, were consulted to identify the most suitable templates for GST proteins. Based on the highest percentage identity and sequence coverage, the best templates were selected for modeling each GST protein. In cases where the templates had the same percentage identity and sequence coverage, we selected the template with the highest resolution for modelling.

### Protein sequence alignment for modeling

T-COFFEE webserver^70^ was used for aligning the GST proteins and the template sequences. The aligned files were downloaded in FASTA format and modified to generate files to be used for protein modelling^71^.

### Protein modeling, optimization and validation

The MODELLER v9.21 program^71^ was used to build GST models. Multiple structures were produced by Modeller 9.21. The model with the best DOPE assessment was selected as the output structure to be used. The structures modeled were viewed using PyMOL^72^. The model for each GST was then subjected to evaluation for stereochemistry and energetic quality at the Structural Analysis and Verification Server (http://servicesn.mbi.ucla.edu/SAVES/) and ProsaII (https://prosa.services.came.sbg.ac.at/)^73^. Based on the validation results, the protein models were then refined on the GalaxyWeb Refiner server^74^. After refinement, the models were again subjected to evaluation and validation using programs such as ERRAT^75^, Verify3D^76^, PROCHECK^77,78^, and RAMPAGE^79^ and ProsaII^73^.

## Results and discussion

### Two different sizes of GSTs present in *Cryptosporidium* species

Genome data mining of 15 *Cryptosporidium* species revealed the presence of 3 GST genes in each of the species genomes (Table 2). The presence of more than one GST gene is common in eukaryotic species^23^. Among 45 GSTs, 30 were found to have the characteristic GST motifs^20,27^, such as the N-terminal domain, which houses the G site, and C terminal domain, which determines the substrate specificity (H-site) (Table 2 and Fig. S1). The remaining 15 GSTs have one of the characteristics GST motifs indicating either these sequences are diverse or fragmented or not properly annotated (Table 2). These GSTs were considered incomplete and were not included for further analysis unless indicated. Future genome editing and better gene prediction programs will help in getting the complete sequences for these GSTs and possibly predicting characteristic N- and C-terminal motifs. In total, 30 GSTs were taken for further analysis. Analysis of GST protein sizes revealed the presence of two different lengths of GSTs in *Cryptosporidium* species (Table 2). One type of GST protein is shorter in size with amino acids ranging between 157 and 268, and another type of GST protein is longer in size, with amino acids ranging between 373 and 466 (Table 2). GSTs from *Cryptosporidium* species seem to be the longest in amino acid length, as most of the GSTs reported in other organisms to date are 200–250 amino acids in length^23^. Furthermore, it can be noted that the addition in length is found only on the outer N- and C-terminal regions, with the center of the protein containing the GST-superfamily domains (Table 2). In order to assess whether *Cryptosporidium* species GST proteins are indeed properly annotated gene products, we further analyzed the gene structure. Interestingly, all the longer GSTs had a single exon, thus no introns, but shorter GSTs were the products of 1–4 exons (Table 2). This could be indicative of shorter GSTs being prone to having multiple isoforms owing to gene shuffling. Due to presence of these multiple introns, the production of more diverse short GSTs can be expected compared to longer GSTs^80^.

**Table 2:** Glutathione transferase (GST) analysis in *Cryptosporidium* species.

Species	Total number of GSTs	GST number	Protein ID	Protein size (no of amino acids)	Characteristic GST motifs location		Gene structure (no. of exons)
					N terminal	C terminal	
*Cryptosporidium andersoni* isolate 30847	3	GST1	cand_012830^a^	197	12–97	95–195	3 exons
		GST2	cand_023790^a^	466	67–149	166–319	1 exon
		GST3	OII73498.1^b^	260	–	124–235	1 exon
*Cryptosporidium hominis* isolate TU502_2012	3	GST1	ChTU502y2012_407g2365^a^	186	1–62	64–186	2 exons
		GST2	ChTU502y2012_421g0615^a^	428	69–151	146–315	1 exon
		GST3	ChTU502y2012_303g0055/OLQ15919.1^a^	268	–	153–236	1 exon
*Cryptosporidium hominis* isolate 30976	3	GST1	GY17_00002363^a^	186	1–62	60–183	2 exons
		GST2	GY17_00000733^a^	428	69–151	146–315	1 exon
		GST3	PPS94453.1^b^	268	–	152–236	1 exon
*Cryptosporidium hominis* TU502	3	GST1	XP_667744.1^b^	161	1–62	64–161	1 exon
		GST2	Chro.80347^a^	428	69–151	146–315	1 exon
		GST3	XP_666781.1^b^	268	–	154–236	1 exon
*Cryptosporidium hominis* UdeA01	3	GST1	CUV07467.1^b^	161	1–62	64–161	1 exon
		GST2	CHUDEA8_2970^a^	428	69–151	146–315	1 exon
		GST3	CUV04748.1^b^	268	–	154–236	1 exon
*Cryptosporidium meleagridis* strain UKMEL1	3	GST1	CmeUKMEL1_03350^a^	193	9–94	96–193	3 exons
		GST2	CmeUKMEL1_14570 ^a^	428	69–151	146–315	1 exon
		GST3	CmeUKMEL1_05845^a^	268	31–118	101–243	1 exon
*Cryptosporidium parvum* Iowa II	3	GST1	cgd7_4780^a^	186	1–62	60–183	2 exons
		GST2	cgd8_2970^a^	429	69–151	146–315	1 exon
		GST3	cgd2_3730^a^	268	–	156–236	1 exon
*Cryptosporidium tyzzeri* isolate UGA55	3	GST1	CTYZ_00001095^a^	186	1–62	60–186	2 exons
		GST2	CTYZ_00000322^a^	429	69–151	146–315	1 exon
		GST3	TRY52903.1^b^	268	–	153–236	1 exon
*Cryptosporidium ubiquitum* isolate 39726	3	GST1	cubi_03151^a^	213	1–89	91–213	4 exons
		GST2	cubi_03523^a^	428	69–151	146–315	1 exon
		GST3	XP_028873506.1^b^	266	–	159–235	1 exon
*Cryptosporidium muris* RN66	3	GST1	XP_002141168.1^b^	160	1–60	58–158	2 exons
		GST2	XP_002140043.1^b^	466	–	211–312	1 exon
		GST3	XP_002142877.1^b^	260	–	164–233	1 exon
*Cryptosporidium baileyi* strain TAMU–09Q1	3	GST1	JIBL01000090.1^b^	156	1–57	59–156	1 exon
		GST2	JIBL01000106.1^b^	390	36–118	113–275	1 exon
		GST3	JIBL01000138.1^b^	236	1–87	69–223	1 exon
*Cryptosporidium viatorum* isolate UKVIA1	3	GST1	QZWW01000010.1^b^	161	1–62	64–161	1 exon
		GST2	QZWW01000018.1^b^	428	69–151	146–315	1 exon
		GST3	QZWW01000026.1^b^	249	–	134–217	1 exon
*Cryptosporidium* sp. *chipmunk* LX–2015	3	GST1	JXRN01000042.1^b^	205	1–106	108–205	1 exon
		GST2	JXRN01000009.1^b^	425	69–151	–	1 exon
		GST3	JXRN01000023.1^b^	250	–	135–217	1 exon
*Cryptosporidium ryanae* isolate 45,019	3	GST1	VHLK01000064.1^b^	166	–	37–154	1 exon
		GST2	VHLK01000046.1^b^	373	36–118	113–274	1 exon
		GST3	VHLK01000056.1^b^	230	1–85	89–221	1 exon
*Cryptosporidium bovis* isolate 42,482	3	GST1	VHIT01000033.1^b^	147	–	30–142	1 exon
		GST2	VHIT01000012.1^b^	376	21–103	98–264	1 exon
		GST3	VHIT01000028.1^b^	227	1–85	98–221	1 exon

The GST number in column 2 is an indication of the number of GSTs that a specific species possesses. Whilst the number on column 3 indicates the group the protein belongs to (based on the percentage identity)^20,23,26–28^.

^a^Protein ID from CryptoDatabase.

^b^Protein ID from NCBI database.

–, characteristic GST domain not identified.

### *Cryptosporidium* species GSTs are cytosolic in nature

Most of the GSTs identified in organisms are cytosolic in nature, with the exception of GSTs belonging to the classes MAPEG and Kappa (mitochondrial) (Table S1). In order to identify the cellular localization, we subjected *Cryptosporidium* species GST protein sequences to the TMHMM Server v. 2.0 for the prediction of transmembrane helices in their structure^65^ and the BUSCA server^64^ for identifying possible localization in a cell. TMHMM prediction revealed that none of the *Cryptosporidium* species GSTs had transmembrane helices, indicating they were soluble and thus possibly cytosolic (Table S2). To authenticate our results, we also subjected 395 GSTs belonging to 17 different classes to TMHMM prediction (Table S3). The TMHMM predicted the presence of no transmembrane helices in previously designated cytosolic GSTs, whereas transmembrane helices were predicted for previously designated microsomal GSTs (Table S3). This indicated that the TMHMM results on the prediction of no transmembrane helices in *Cryptosporidium* species GSTs were in agreement with previous annotations. Furthermore, BUSCA indicated that all 30 *Cryptosporidium* species GSTs were cytosolic (Table S4). Based on these in silico results, we concluded that the 30 *Cryptosporidium* species GSTs were cytosolic in nature.

### *Cryptosporidium* species GSTs belongs to new classes

**Figure 1:**
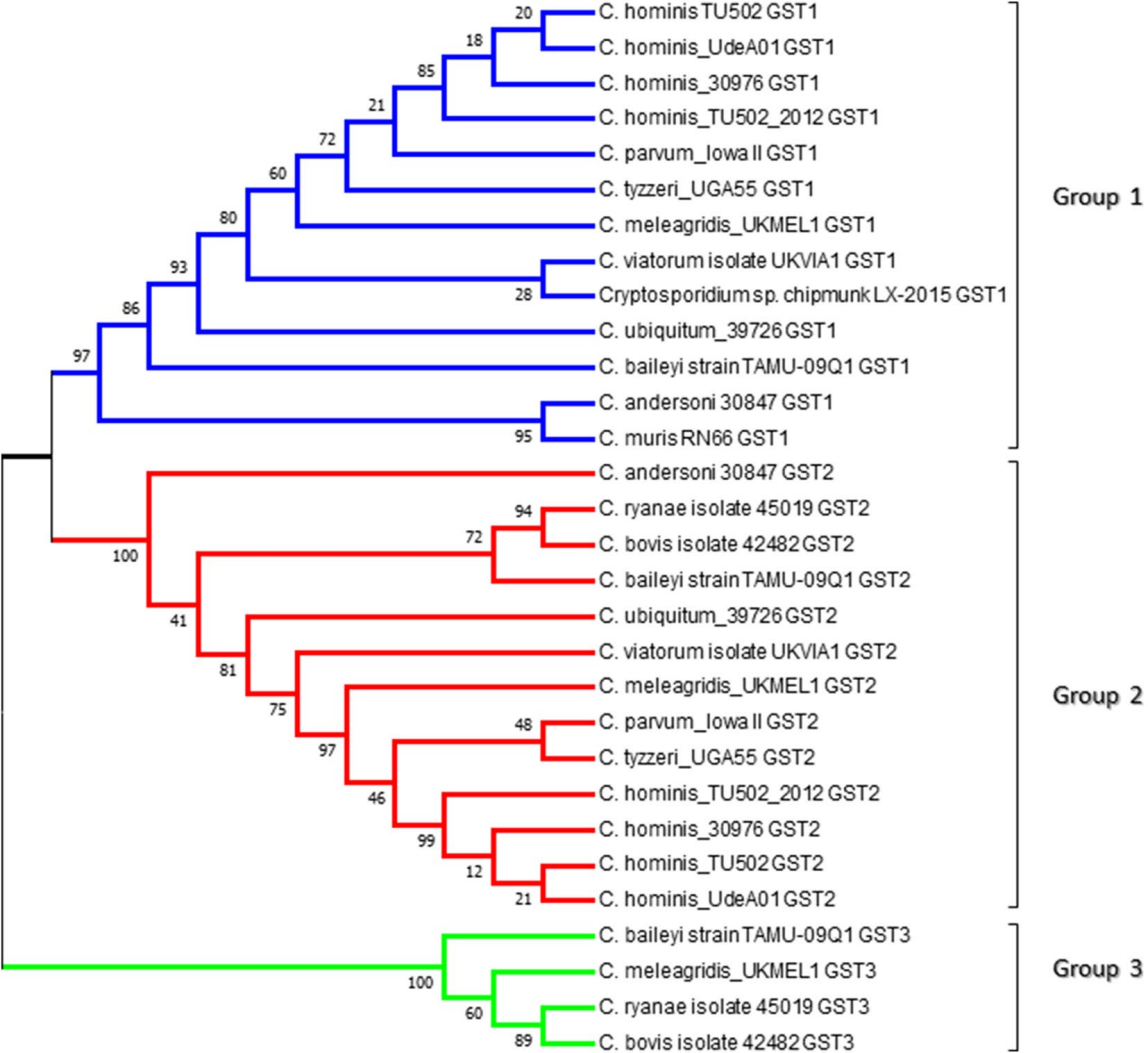
Phylogenetic analysis of glutathione transferase (GST) proteins from *Cryptosporidium* species. The evolutionary history was inferred by using the maximum likelihood method based on the JTT matrix-based model^63^. Evolutionary analyses were conducted in MEGA7^62^. The percentage of trees (bootstrap value) in which the associated taxa clustered together is shown next to the branches.

Phylogenetic analysis of *Cryptosporidium* species GSTs revealed that the 30 GSTs could be grouped into three different groups (Fig. 1). The shorter GSTs were grouped together (Group 1) and so were the longer GSTs (group 2). Interestingly, despite the short amino acid length, four GSTs diverged from these two groups (Group 3) (Fig. 1). Analysis of the amino acid percentage identity among *Cryptosporidium* species GSTs further confirmed that they indeed belonged to three different groups. Group 1 GSTs shared an amino acid percentage identity of 54–100%, whereas groups 2 and 3 shared identities of 48–100% and 42–71%, respectively. Group 3 GSTs had 13–21% identity with Group 2 GSTs and 14–22% identity to Group 1 GSTs. The percentage identity between Groups 1 and 2 was 17–25%. This indicates that all three groups of *Cryptosporidium* species GSTs indeed belonged to three different classes as the percentage identity between these groups was below 25–30%, qualifying them to be their own class^20,23,26–28^.

**Figure 2:**
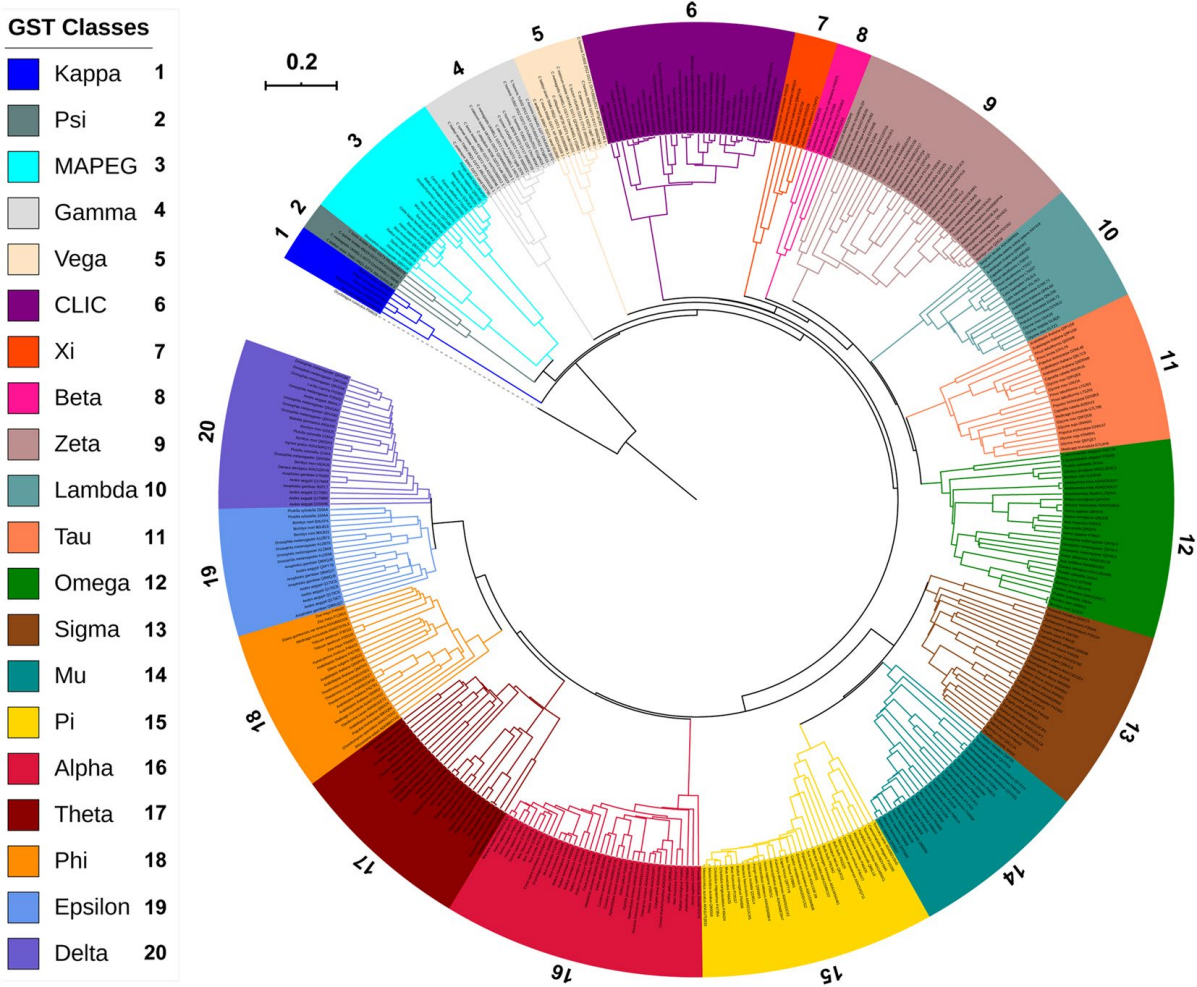
Phylogenetic tree of the glutathione transferases (GSTs) protein sequences of *Cryptosporidium* species with GSTs from 17 different GST classes. Thioredoxin from *Oryctolagus cuniculus* (protein ID: P08628) is used as an outgroup. Three new GST classes reported in this study from *Cryptosporidium* species named Vega, Gamma and Psi are also shown in the tree. A high-resolution phylogenetic tree is provided in Supplementary Dataset 2.

Although the above results clearly indicated that *Cryptosporidium* species GSTs belong to three different groups, it was still not clear whether they fell under one of the GST classes described in the literature (Table S1). Thus, the comprehensive phylogenetic analysis of proteins belonging to 17 known GST classes and *Cryptosporidium* species GSTs was carried out (Fig. 2). Phylogenetic analysis revealed that *Cryptosporidium* species GSTs did not align with any of the 17 pre-existing GST classes and formed three new groups (Fig. 2). This clearly indicates that *Cryptosporidium* species GSTs belong to three different new GST classes. Thus, we named groups 1, 2 and 3 of *Cryptosporidium* GSTs Vega (ϑ), Gamma (γ) and Psi (ψ), respectively. A point to be noted is that all the GST proteins aligned together as per their GST class on the phylogenetic tree, indicating our phylogenetic analysis is correct and thus we conclude that *Cryptosporidium* species GSTs indeed belong to new GST classes.

### *Cryptosporidium parvum* GST1 of Vega class has atypical thioredoxin-like fold

Identification of three new GST classes in *Cryptosporidium* species in this study necessitated examination of the structural aspects of these new classes to see if any deviations or novel folds might be present, compared to the canonical structure of GSTs^20,27^. Analysis of the primary structure revealed that all *Cryptosporidium* species GSTs have N- and C-terminal regions characteristic of GSTs that usually contain a G-site and H-site^20,27^, respectively (Table 2 and Fig. S1). All GSTs have the highly conserved proline amino acid residue (Fig. S1) that is part of the *cis*-Pro loop responsible for connecting the N- and C-terminal regions in order to maintain the GST structural integrity^81^. It was observed from Fig. S1 that Psi class GSTs have a Tyr residue in the N-terminal domain in close proximity to the expected active site Tyr. The same was observed with the Vega class GSTs with the expectation *of C. muris* and *C. baileyi*. Vega and Psi GSTs have a few tyrosine residues in the N-terminal region, but they are not at a position that is considered part of an active site^20,27^ (Fig. S1). Similarly, the majority of the Gamma class GSTs consist of an active site Tyr residue with the exception *C. andersoni, C. baileyi, C. ryanae* and *C. bovis* species. In these species, Phe replaces the active site Tyr residue. Mutagenesis studies have shown that the presence of Phe at the supposed position of the active site Tyr significantly reduces the catalytic activity. This highlights the critical role played by the active site Tyr in the catalytic activity of GST^82,83^. The effect of these mutations in the context of *Cryptosporidium* GSTs is yet to be studied.

Multiple sequence alignments of Vega and Gamma GSTs revealed that amino acids in the N- and C-terminal regions of these GSTs are highly conserved (Fig. S1). For this reason, we selected *C. parvum* GSTs 1 and 2 (C*p*GST1 and C*p*GST2) as representative of the Vega and Gamma GST classes for structural analysis along with *C. meleagridis* UKMEL1 GST3 (C*m*GST3) for the Psi class. Structural analysis of the three GSTs was carried out using in silico homology modeling. The structural analysis was aimed at assessing only the secondary structural elements that are characteristic of GST proteins^20,27^. These GST models are not aimed to assess the binding affinities or the residues involved in binding to different ligands. In order to build 3D models we performed a template search at three different webpages, namely NCBI^67^, PHYRE^69^ and I-TASSER^68^. The templates found were of low sequence identity but had relatively good coverage (Table S5). This was expected, since these GSTs are new. We then proceeded to build 3D models using a multiple template method, as this approach is known to improve the quality of homology models^84^. We built 3D models for all three GSTs, attempting single and multiple templates, while also using different combinations of the available templates listed in Table S5. The best 3D models with good quality closest to the templates were chosen for the structural analysis.

**Figure 3:**
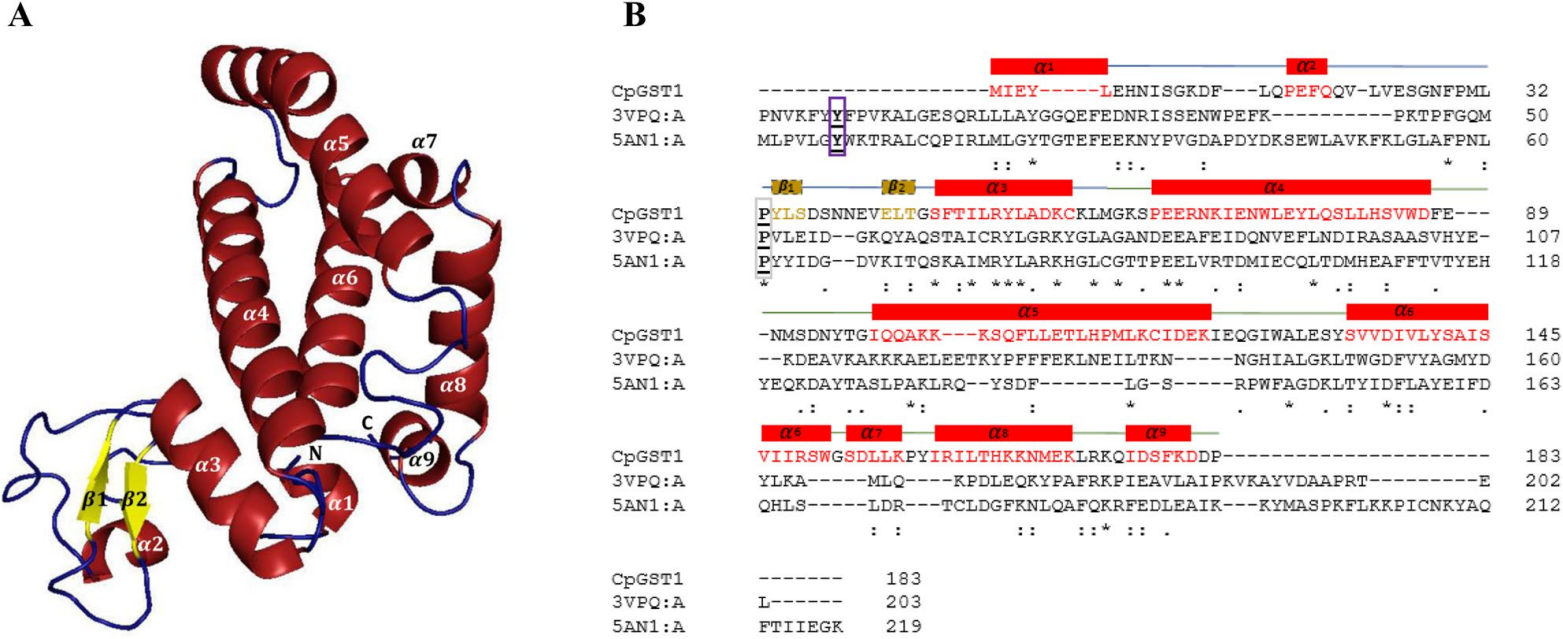
In silico structural analysis of Vega class representative *Cryptosporidium parvum* glutathione transferase 1 (C*p*GST1). 3D model of C*p*GST1 (**A**) and its amino acid sequence alignment with templates (**B**). Secondary structural annotations were done as per modeled structure where α-helices and corresponding amino acids are colored in red while the β-sheets and their corresponding amino acids are colored in yellow. The active-site tyrosine and the *cis*-proline residues are boxed in purple and grey respectively. The template Protein Data Bank codes, 3VPQ-A and 5AN1-A, represents GST protein crystal structures from *Bombyx mori* (Sigma class GST) and *Penaeus vannamei* (Mu class GST).

**Figure 4:**
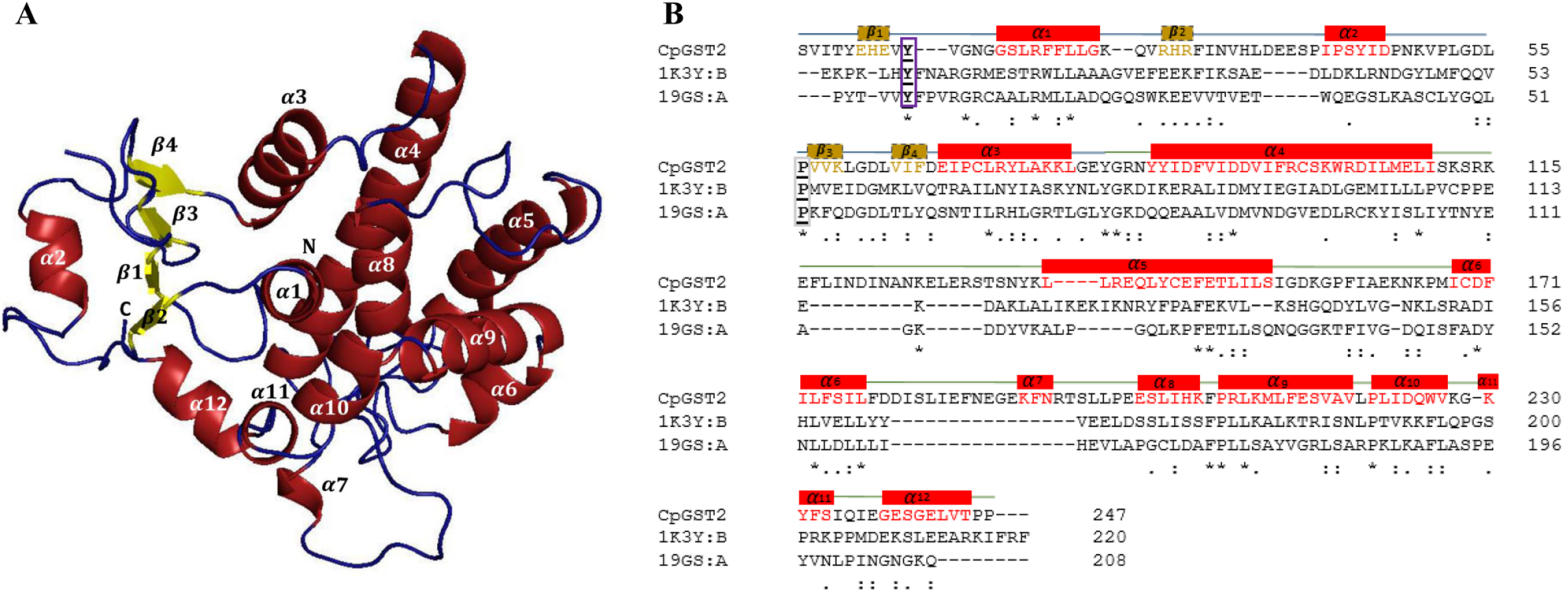
In silico structural analysis of Gamma class representative *Cryptosporidium parvum* glutathione transferase 2 (C*p*GST2). 3D model of C*p*GST2 (**A**) and its amino acid sequence alignment with templates (**B**). Secondary structural annotations were done as per modeled structure where α-helices and corresponding amino acids are colored in red while the β-sheets and their corresponding amino acids are colored in yellow. The active-site tyrosine and the *cis*-proline residues are boxed in purple and grey respectively. The template Protein Data Bank codes, 1K3Y-B and 19GS-A, represents GST protein crystal structures of Alpha class (1K3Y-B) and Pi class (19GS-A) GSTs from humans.

**Figure 5:**
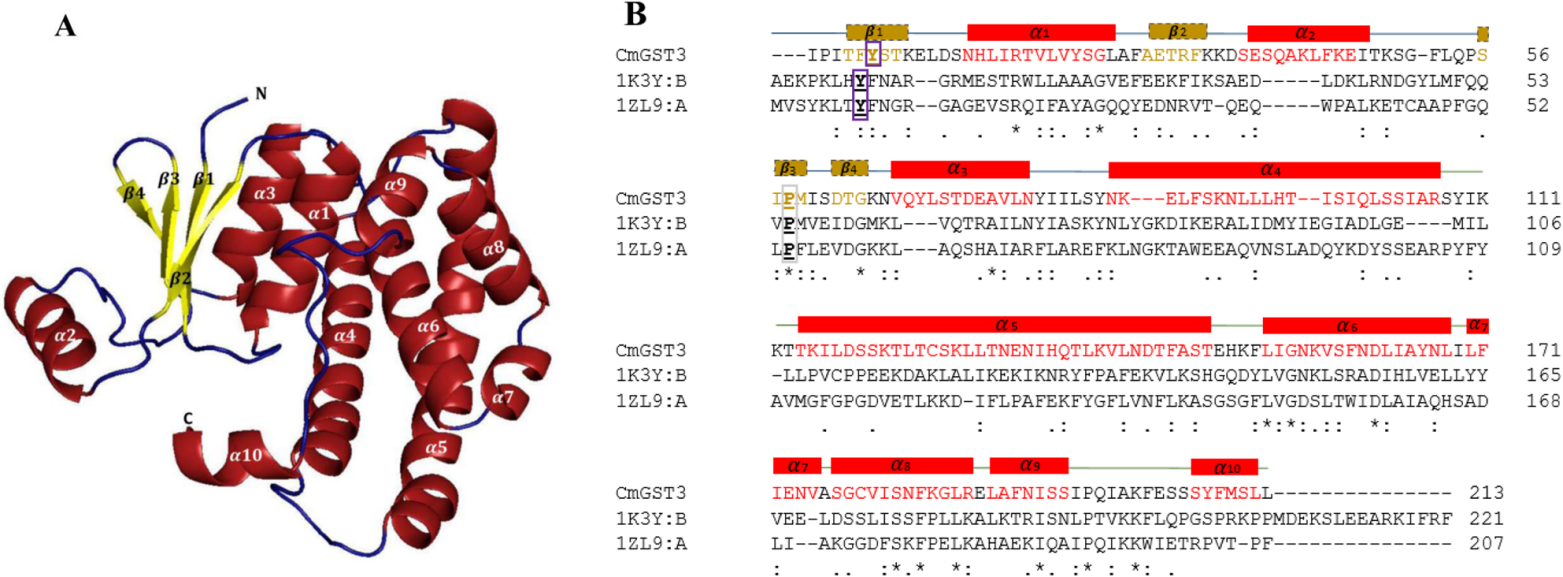
In silico structural analysis of Psi class representative *Cryptosporidium meleagridis* strain UKMEL1 GST3 glutathione transferase 3 (C*p*GST3). 3D model of C*m*GST3 (**A**) and its amino acid sequence alignment with templates (**B**). Secondary structural annotations were done as per modeled structure where α-helices and corresponding amino acids are colored in red while the β-sheets and their corresponding amino acids are colored in yellow. The active-site tyrosine and the *cis*-proline residues are boxed in purple and grey respectively. The template Protein Data Bank codes, 1K3Y-B and 1ZL9-A, represents GST protein crystal structures from Human (Alpha class GST) and *Caenorhabditis elegans* (Pi class GST).

Here, we present the combination of templates that gave C*p*GST1, C*p*GST2 and *Cm*GST3 models. The templates used to model C*p*GST1 were a *Bombyx mori* Sigma class GST (3VPQ-A)^85^ that had 94% coverage and 26% identity and a *Penaeus vannamei* Mu class (5AN1-A)^86^ with 98% coverage and 23% identity (Fig. 3 and Table S5). For C*p*GST2 the templates were both from *Homo sapiens* Alpha class (1K3Y-B)^87^ and Pi class (19GS-A)^88^, with sequence identity at 21%, coverage at 94% and 22% identity and 84% coverage (Fig. 4 Table S5), respectively. The C*m*GST3 templates used were from *Caenorhabditis elegans* Pi class GST (1ZL9-A) (https://www.rcsb.org/structure/1ZL9) with 94% coverage and 21% identity and a *Homo sapiens* Alpha class (1K3Y-B)^87^ with 98% coverage and 22% identity (Fig. 5 and Table S5).

For each GST, 20 models were built using the MODELLER v9.21 program^71^. The best model evaluated by DOPE score was selected and subjected to structural quality analysis. The selected model for each GST was then refined on the GalaxyWeb Refiner server^74^ and further subjected to structural quality evaluation using different programs such as ERRAT^75^, Verify3D^76^, PROCHECK^77,78^, RAMPAGE^79^ and ProsaII^73^. The overall quality of the models was assessed by the combination of these programs’ values and by comparing these with the templates’ structural evaluation scores (Tables S6 and S7). The models generated for C*p*GST1 and C*p*GST2 were found to be of good quality, as different structural validation programs indicated that the quality of the model structures was close to the quality of the template structures (Tables S6 and S7). The model generated for C*m*GST3 had all parameters in acceptable range including Z-score of − 3.68 indicating the model is of good quality with the exception of Verify3D where 26% residues had an average 3D-1D score > 0.2 (Tables S6 and S7). The three GST models generated in the study, along with their corresponding sequence alignments with their templates, are presented in Figs. 3, 4 and 5.

Structural analysis revealed the presence of 2β-sheets and 3α-helices in the N-terminal region and 6α-helices in the C-terminal region of C*p*GST1 (Fig. 3). The overall structure of C*p*GST1 at the N-terminal domain seems completely different compared to the canonical GST N-terminal domain^20,27^. The N-terminal region of C*p*GST1 did not have the typical thioredoxin-like fold, nor did it follow the βαβ-α-ββα motif; it was rather composed of two antiparallel β-sheets and 3α-helices (Fig. 3). It is rare to find GSTs that do not possess the conventional thioredoxin βαβ-α-ββα motif. Kappa class GSTs, which are mitochondrial GSTs, are the closest GSTs that do not follow the traditional thioredoxin fold but have still been found to carry out a similar molecular function as conical GSTs^89–91^. This is also common for MAPEG GST and the mPGES-1 (microsomal ProstaGlandin E-Synthase type 1) subfamily of proteins, as they too are a group of structurally unrelated proteins with GSH transferase activities^23,91^. Because the GST superfamily shares such vast variations in terms of their structural conformation, this ααββα conformation of C*p*GST1 can be considered a unique Vega class feature.

In contrast to the C*p*GST1 model, the C*p*GST2 and C*m*GST3 models N-terminal domain follows the thioredoxin-like fold, which is characteristic of cytosolic enzymes in the GST superfamily^20,22,27^. The N-terminal domain was complete with 4β-sheets and 3α-helices following a βαβ and ββα arrangement with the two motifs linked by an α2 (Figs. 4 and 5). The C-terminal domain contains helices with each model C*p*GST2 and C*m*GST3 having a varying number of helices (Figs. 4 and 5). It has been suggested that an increase in the number of helices in the C-terminal domain, may allow for a broader substrate range and/or offer a deeper catalytic pocket that facilitates the conjugation of larger substrates^92,93^.

## Conclusions

In this genomic era, in silico based comparative studies at genome level or at protein family level have become an important tool to uncover novel aspects in organisms. This study is such an example, where genomes of *Cryptosporidium* species were mined for glutathione transferases (GSTs), enzymes playing a key role in cellular defense and detoxification that are also a potential drug target against pathogens and metabolic disorders. Analysis revealed an interesting feature, namely the presence of two different sizes of GSTs (short and long) in these species. The longer GST proteins were found to be longer than the GSTs found in other organisms, with the size attributed to C- and N-terminal extensions. One of the major findings of the study is the identification of GSTs belonging to three new GST classes in *Cryptosporidium* species. In addition, *Cryptosporidium parvum* GST1 had an atypical thioredoxin fold in the N-terminal region with an αα-ββ-α motif rather than the typical thioredoxin-like fold with a βαβ-α-ββα motif. Future study includes functional and structural (X-ray or NMR) characterization of *Cryptosporidium* species GSTs. The study results serve as reference for future mining and annotation of GSTs *Cryptosporidium* species.

## Supplementary information

Supplementary Information.

Supplementary Dataset 1.

Supplementary Dataset 2.
