# Supplementary material for: Comparative analyses and structural insights of new class glutathione transferases in Cryptosporidium species
Source: Sci Rep. 2020 Nov 23;10:20370. doi: 10.1038/s41598-020-77233-5 (PMC7683740; doi:10.1038/s41598-020-77233-5)
Supplement: Supplementary file 1 — Supplementary Information. [file 41598_2020_77233_MOESM1_ESM.docx]

**Comparative Analyses and Structural Insights of New Class Glutathione Transferases in *Cryptosporidium* Species**

Mbalenhle Sizamile Mfeka ^1^, José Martínez-Oyanedel ^2^, Wanping Chen ^3^, Ikechukwu Achilonu ^4^, Khajamohiddin Syed ^5^*, Thandeka Khoza ^1^*

^1^ Department of Biochemistry, School of Life Sciences, University of KwaZulu-Natal (Pietermaritzburg campus), Scottsville, 3209, KwaZulu-Natal, South Africa.

^2^ Laboratorio de Biofísica Molecular, Departamento de Bioquímica y Biología Molecular, Facultad de Ciencias Biológicas, Universidad de Concepción, Barrio Universitario S/N, Casilla 160_C, Concepción, Chile.

^3^ Department of Molecular Microbiology and Genetics, University of Göttingen, Göttingen 37077, Germany; chenwanping1@foxmail.com.

^4^ Protein Structure-Function Research Unit, School of Molecular and Cell Biology, University of the Witwatersrand, Braamfontein, Johannesburg, South Africa.

^5^ Department of Biochemistry and Microbiology, Faculty of Science and Agriculture, University of Zululand, 1 Main Road Vulindlea, KwaDlangezwa 3886, South Africa.

* Corresponding authors’ email:

khajamohiddinsyed@gmail.com and khozat1@ukzn.ac.za

**Table S1. Information on different glutathione transferase classes found in organisms.**

| **GST Class** | **Cellular localization** | **General Information** | **Reference** |
| --- | --- | --- | --- |
| Alpha | Cytosol | Found in a broad range of species. Involved in the biosynthesis of sex steroids and keto-steroid isomerase activity. | ^1^ |
| Beta | Cytosol | Typically found in bacterial species. Known for conjugating antibiotics, assisting in antibiotic resistance to other organisms. | ^2^ |
| CLIC | Cytosol | Found in a broad range of species. Enter intracellular membranes and form membrane channels. | ^3^ |
| Delta and Epsilon | Cytosol | Typically found in insects. Thought to contribute to detoxication or antioxidative stress during development. Delta GSTs are also involved in oogenesis. | ^4,5^ |
| Kappa | Mito-chondrial | To date, found in primates and mice. Oligomerization of adiponectin. | ^6^ |
| Lambda | Cytosol | Typically found in plants. Function is not yet known as they have no detectable GSH-conjugating activity. | ^7^ |
| MAPEG | Microsomal | Found in a broad range of species. Involved in production of leukotrienes and prostaglandin E and are mediators of inflammation. | ^8^ |
| Mu | Cytosol | Found in a broad range of species. Forms inhibitory complexes with ASK1, another member of the MAP kinase pathway. | ^9^ |
| Omega | Cytosol | Found in a broad range of species. Catalyzes reduction and thioltransferase reactions. | ^10^ |
| Phi | Cytosol | Typically found in plants. Inhibits oxidative damage through the removal of endogenous cytotoxic hydroperoxides. | ^11^ |
| Pi | Cytosol | Found in a broad range of species. Regulates JNK and TRAF signaling and catalyzes the S-glutathionylation reactions. | ^12^ |
| Sigma | Cytosol | Found in a broad range of species. Involved in prostaglandin synthesis by isomerization of PGH_2_ – PGD_2._ | ^3^ |
| Tau | Cytosol | Typically found in plants.  Involved in reactive oxygen species scavenging and improves plant chilling tolerance | ^13^ |
| Theta | Cytosol | Found in a broad range of species.  Has dichloromethane dehalogenase activity for the degradation of dichloromethane to obtain energy | ^2^ |
| Xi | Cytosol | Typically found in bacteria, fungi, and archaea.  Aids in extreme haloalkaphilic conditions. | ^14^ |
| Zeta | Cytosol | Found in a broad range of species. Involved in isomerization of maleyacetoacetate to fumaracetoacetate in tyrosine degradation pathway and biotransformation of dichloroacetic acid to glyoxylate | ^15^ |
| Gamma (γ), Vega (ϑ) and Psi (ψ) | Cytosol* | Found in *Cryptosporidium* species. Function is not known. | This work |

Symbol: *, Based on *in silico* prediction. Abbreviations: GSH, Glutathione; ASK1, Apoptosis signal-regulated kinase 1; MAP, Mitogen activated protein; JNK, c-Jun N-Terminal Kinase; TRAF, Tumor necrosis factor receptor (TNF)-associated factor; PGH2, Prostaglandin H2; PGD2, Prostaglandin D2.

**Group 1 (Vega (ϑ))**

CLUSTAL O(1.2.4) multiple sequence alignment

C. andersoni 30847 GST1. MNDSNYPYSVKSPLKLIYFACRGSCDVIRLLLNDKCIP**Y**--------------------- 39

C. muris RN66 GST1 -------------------------------------MI--------------------- 2

C. baileyi strain TAMU-09Q1 GST1 ------------------------------------------------------------ 0

C. ubiquitum_39726 GST1 ---MEYIGSLDNPLRLIYFSCRGTCDAIRLLLVDQEIP**Y**E-------------------- 37

Cryptosporidium sp. chipmunk LX-2015 GST1 -----------------ILYFRGTCDVIRLLLVDQEIP**Y**EGKLKRYCQLIIYTVFSSGIQ 43

C. viatorum isolate UKVIA1 GST1 -----------------------------------ILK**Y**--------------------- 4

C. meleagridis_UKMEL1 GST1 ---MEYIGSLDNPLRLIYFSCRGTCDVIRLLLVDQEIP**Y**--------------------- 36

C. tyzzeri_UGA55 GST1 -----------------------------------MIE**Y**--------------------- 4

C. parvum_Iowa II GST1 -----------------------------------MIE**Y**--------------------- 4

C. hominis_TU502_2012 GST1 -----------------------------------MIE**Y**--------------------- 4

C. hominis_30976 GST1 -----------------------------------MIE**Y**--------------------- 4

C. hominis TU502 GST1 -----------------------------------MIE**Y**--------------------- 4

C. hominis_UdeA01 GST1 -----------------------------------MIE**Y**--------------------- 4

C. andersoni 30847 GST1 -----EEHNIQGKDFLQPEFQNVLLESDNFPIL**P**YLSDPNSEMELTGSLTILRYLGRKCN 94

C. muris RN66 GST1 -----NVFHMKGKDFLQSEFQNVLLESDNFPIL**P**YLSDPNSEIELTGSLTILRYLGRKCN 57

C. baileyi strain TAMU-09Q1 GST1 ------EHNISGKDFLQEEFQQVLIESGNFPML**P**YLSDSNNEVELTGSFTILRYLGEKCK 54

C. ubiquitum_39726 GST1 -----------GKDFLQPEFQHVLAESGNFPML**P**YLSDSNNEVELTGSFTILRYLADKCK 86

Cryptosporidium sp. chipmunk LX-2015 GST1 KFLNILEHNISGKDFLQPEFQQVLVESGNFPML**P**YLSDSNNEVELTGSFTILRYLADKCK 103

C. viatorum isolate UKVIA1 GST1 -----IEHNISGKDFLQPEFQQVLVESGNFPML**P**YLSDSNNEVELTGSFTILRYLADKCK 59

C. meleagridis_UKMEL1 GST1 -----EEHNISGKDFLQPEFQQVLLESGNFPML**P**YFSDSNNEVELTGSFTILRYLADKCK 91

C. tyzzeri_UGA55 GST1 -----LEHNISGKDFLQPEFQQVLVESGNFPML**P**YFSDSNNEVELTGSFTILRYLADKCK 59

C. parvum_Iowa II GST1 -----LEHNISGKDFLQPEFQQVLVESGNFPML**P**YLSDSNNEVELTGSFTILRYLADKCK 59

C. hominis_TU502_2012 GST1 -----LEHNISGKDFLQPEFQQVLVESGNFPML**P**YFSDSNNEVELTGSFTILRYLADKCK 59

C. hominis_30976 GST1 -----LEHNISGKDFLQPEFQQVLVESGNFPML**P**YFSDSNNEVELTGSFTILRYLADKCK 59

C. hominis TU502 GST1 -----LEHNISGKDFLQPEFQQVLVESGNFPML**P**YFSDSNNEVELTGSFTILRYLADKCK 59

C. hominis_UdeA01 GST1 -----LEHNISGKDFLQPEFQQVLVESGNFPML**P**YFSDSNNEVELTGSFTILRYLADKCK 59

****** ***:** **.***:***:** *.*:*****:******. **:

C. andersoni 30847 GST1 LMGNNYEDELQIENWLEYLQLVLNILWEFDSNSDNFNNIQKNKKRGQFLLENLHPMLHNI 154

C. muris RN66 GST1 LMGNNYEDELQIENWFEYLQLVLNILWEFDSNLDSFNNIQKNKKRGQFLLENLHPMLHNI 117

C. baileyi strain TAMU-09Q1 GST1 LMGNNVTERNKIENWLEFLQSLLHSIWDFENNITNYTESQKKKRKSQFLLENLHPMLRSI 114

C. ubiquitum_39726 GST1 LMGNNSEERNRVENWLEFLQSLLHSVWDFENISENYTGVQQTKKKSKFLLDTLHPMLKCI 146

Cryptosporidium sp. chipmunk LX-2015 GST1 LMGKSPEERNKIENWLEYLQSLLHSVWDFENRSDNYTGVQQTKKRSQFLLETLHPMLKCI 163

C. viatorum isolate UKVIA1 GST1 LMGKSPEERNKIENWLEYLQSLLHSVWDFENMSDNYTGIQHTKKKSQFLLETLHPMLKCI 119

C. meleagridis_UKMEL1 GST1 LMGKSPEERNKIENWLEYLQSLLHSLWDFENMSDNYTGIQQTKKKRQFLLETLHPMLKCI 151

C. tyzzeri_UGA55 GST1 LMGKSPEERNKVENWLEYLQSLLHSVWDFENMSDNYTGIQQAKKKSQFLLETLHPMLKCI 119

C. parvum_Iowa II GST1 LMGKSPEERNKIENWLEYLQSLLHSVWDFENMSDNYTGIQQAKKKSQFLLETLHPMLKCI 119

C. hominis_TU502_2012 GST1 LMGKSPEERNKIENWLEYLQSLLHSVWDFENMSDNYTGIQQAKKKSQFLLETLHPMLKCI 119

C. hominis_30976 GST1 LMGKSPEERNKIENWLEYLQSLLHSVWDFENMSDNYTGIQQAKKKSQFLLETLHPMLKCI 119

C. hominis TU502 GST1 LMGKSPEERNKIENWLEYLQSLLHSVWDFENMSDNYTGIQQAKKKSQFLLETLHPMLKCI 119

C. hominis_UdeA01 GST1 LMGKSPEERNKIENWLEYLQSLLHSVWDFENMSDNYTGIQQAKKKSQFLLETLHPMLKCI 119

***:. :. ::***:*:** :*: :*:*:. .:. *: *:: :***:.*****: *

C. andersoni 30847 GST1 QVRLDNGKKWIMEEYSVADIMLYTVVSAIIRSWGYEILQPYDK----------------- 197

C. muris RN66 GST1 QARLDNGKKWILEEYSVADIMLYTVVSAIIRSWGYEILQPYDK----------------- 160

C. baileyi strain TAMU-09Q1 GST1 NDKIESN-LWALNDYSIIDIVLYSTISVVIKLWSIDLLKPYEK----------------- 156

C. ubiquitum_39726 GST1 DDKIEQG-FWALESYSVVDIVLYSTISVVIRSWGSDLLKPYIRILSHKKNMEKLRKQIDS 205

Cryptosporidium sp. chipmunk LX-2015 GST1 DEKIEHG-VWVLDSYSVVDIVLYSAISVIIRSWGGDLLKPYTR----------------- 205

C. viatorum isolate UKVIA1 GST1 DEKIEQG-VWALDSYSVVDVVLYSAISVVIRSWGIDLLKPYIK----------------- 161

C. meleagridis_UKMEL1 GST1 DEKIEQG-VWALESYSVVDIVLYSAISVVIRSWGSDLLKPYIR----------------- 193

C. tyzzeri_UGA55 GST1 DEKIEQG-VWALEYYSVVDIVLYSAISVIIRSWGSDLLKPYIRILTHKKNMEKLRKQIDS 178

C. parvum_Iowa II GST1 DEKIEQG-IWALESYSVVDIVLYSAISVIIRSWGSDLLKPYIRILTHKKNMEKLRKQIDS 178

C. hominis_TU502_2012 GST1 DEKIEQG-VWALESYSVVDIVLYSAISVIIRSWGSDLLKPYIRILTHKKNMEKLRKQIDS 178

C. hominis_30976 GST1 DEKIEQG-VWALESYSVVDIVLYSAISVIIRSWGSDLLKPYIRILTHKKNMEKLRKQIDS 178

C. hominis TU502 GST1 DEKIEQG-VWALESYSVVDIVLYSAISVIIRSWGSDLLKPYIR----------------- 161

C. hominis_UdeA01 GST1 DEKIEQG-VWALESYSVVDIVLYSAISVIIRSWGSDLLKPYIR----------------- 161

: ::: . * :: **: *::**:.:*.:*: *. ::*:** :

C. andersoni 30847 GST1 -------- 197

C. muris RN66 GST1 -------- 160

C. baileyi strain TAMU-09Q1 GST1 -------- 156

C. ubiquitum_39726 GST1 FKDDPRRF 213

Cryptosporidium sp. chipmunk LX-2015 GST1 -------- 205

C. viatorum isolate UKVIA1 GST1 -------- 161

C. meleagridis_UKMEL1 GST1 -------- 193

C. tyzzeri_UGA55 GST1 FKDDPRRF 186

C. parvum_Iowa II GST1 FKDDPRRF 186

C. hominis_TU502_2012 GST1 FKDDPRRF 186

C. hominis_30976 GST1 FKDDPRRF 186

C. hominis TU502 GST1 -------- 161

C. hominis_UdeA01 GST1 -------- 161

**Group 2 (Gamma (γ))**

CLUSTAL O(1.2.4) multiple sequence alignment

C. andersoni 30847 GST2 MIGVNSSISTGVASFSRDLSSLPGTSFIP-----AKAGSPQKSPSNLYGVIQAPRATSIR 55

C. ubiquitum_39726 GST2 MNNIGAGTTASPKNIATKVSSELNEIYSPKMSNLIRSNAPCRL---TSNRVMIPSKSTYR 57

C. viatorum isolate UKVIA1 GST2 MNNIEASTTASPKIIAAKVSSELNEIYSPKMSKLVRNNIPCRL---TTNRVMAPSRSTYR 57

C. meleagridis_UKMEL1 GST2 MNSKETSTISSPKIIASKISSESSEIYSPKISTLTRNSIPCRL---TSNRVMASSKSTYR 57

C. hominis_TU502_2012 GST2 MNNKETSTIPSPKTIASKISSELSEIYSPKMSTLVRNNIPCRL---TSNRVMAPSKSTYR 57

C. hominis_UdeA01 GST2 MNNKETSTIPSPKTIASKISSELSEIYSPKMSTLVRNNIPCRL---TSNRVMAPSKSTYR 57

C. hominis_30976 GST2 MNNKETSTIPSPKTIASKISSELSEIYSPKMSTLVRNNIPCRL---TSNRVMAPSKSTYR 57

C. hominis_TU502 GST2 MNNKETSTIPSPKTIASKISSELSEIYSPKMSTLVRNNIPCRL---TSNRVMAPSKSTYR 57

C. parvum_Iowa II GST2 MNNKETSTIPSPNIIASKISSELSEIYSPKMSTLVRNNIPCRL---TSNRVMAPSKSTYR 57

C. tyzzeri_UGA55 GST2 MNNKETSTIPSPKIIASKISSELSEIYSPKMSTLVRNNIPCRL---TSNRVMAPSKSTYR 57

C. baileyi strain TAMU-09Q1 GST2 ---------------------------------PMRSNVPCRL---TNNRIMMPSKSTVK 24

C. ryanae isolate 45019 GST2 ---------------------------------MSKTIIPFRL---TSNRIMIPSRSAVR 24

C. *bovis* isolate 42482 GST2 ---------------------------------------------------MIPSRSAVR 9

:: :

C. andersoni 30847 GST2 VMLPVRDIGDLTVVTYEHQAFVGCGGSLRFFLLGKQVKHKFINVPVDKDNPIPDYIESSK 115

C. ubiquitum_39726 GST2 VILPVRDIGDLSVITYEHEI**Y**LGNGGSLRFFLLGKQVRHRFINVPLDEENPIPSYIDSDK 117

C. viatorum isolate UKVIA1 GST2 VILPVRDIGDLSVITYEHEV**Y**VGNGGSLRFFLLGKQVRHRFINVHLDEEDPIPSYIDPNK 117

C. meleagridis_UKMEL1 GST2 VILPVRDIGDLSVITYEHEV**Y**VGNGGSLRFFLLGKQVRHRFINVHLDEESPIPSYIDPNK 117

C. hominis_TU502_2012 GST2 VILPVRDIGDLSVITYEHEV**Y**VGNGGSLRFFLLGKQVRHRFINVHLDEESPIPSYIDPNK 117

C. hominis_UdeA01 GST2 VILPVRDIGDLSVITYEHEV**Y**VGNGGSLRFFLLGKQVRHRFINVHLDEESPIPSYIDPNK 117

C. hominis_30976 GST2 VILPVRDIGDLSVITYEHEV**Y**VGNGGSLRFFLLGKQVRHRFINVHLDEESPIPSYIDPNK 117

C. hominis_TU502 GST2 VILPVRDIGDLSVITYEHEV**Y**VGNGGSLRFFLLGKQVRHRFINVHLDEESPIPSYIDPNK 117

C. parvum_Iowa II GST2 VILPVRDIGDLSVITYEHEV**Y**VGNGGSLRFFLLGKQVRHRFINVHLDEESPIPSYIDPNK 117

C. tyzzeri_UGA55 GST2 VILPVRDIGDLSVITYEHEV**Y**VGNGGSLRFFLLGKQVRHRFINVHLDEESPIPSYIDPNK 117

C. baileyi strain TAMU-09Q1 GST2 VVLPVRDIGELSVVTYEHDI**F**VGNGGSIRFFLLGKQVRHRFINVPLDEENPIPSFIDSSR 84

C. ryanae isolate 45019 GST2 VVLPVRDIGELTVITFEHDI**F**VGNGGSIRFFLLGKQVRHRFVNVPLDEEKPIPSYIDSSR 84

C. *bovis* isolate 42482 GST2 VVLPVRDIGELTVVTFEHNV**F**VGNGGSIRFFLLGKQVRHRFVNVPLDEEKPIPSYIDSSR 69

*:*******:*:*:*:**: ::* ***:*********:*:*:** :*::.***.:*: .:

C. andersoni 30847 GST2 VPLGEL**P**IIKLGDLVIFDEIPCLRFLAKKLGEYGRNYYIDFVIDDVVMRCNRWRDILMDL 175

C. ubiquitum_39726 GST2 VPLGDL**P**IVKLGDLVIFDEIPCLRYLAKKLGEYGRNYYIDFVIDDVIFRCSKWRDVLMDL 177

C. viatorum isolate UKVIA1 GST2 VPLGDL**P**VVKLGDLVIFDEIPCLRYLAKKLGEYGRNYYIDFVIDDVIFRCSRWRDILMEL 177

C. meleagridis_UKMEL1 GST2 VPLGDL**P**IVKLGDLVIFDEIPCLRYLAKKLGEYGRNYYIDFVIDDVIFRCSKWRDILMEL 177

C. hominis_TU502_2012 GST2 VPLGDL**P**VVKLGDLVIFDEIPCLRYLAKKLGEYGRNYYIDFVIDDVIFRCSKWRDILMEL 177

C. hominis_UdeA01 GST2 VPLGDL**P**VVKLGDLVIFDEIPCLRYLAKKLGEYGRNYYIDFVIDDVIFRCSKWRDILMEL 177

C. hominis_30976 GST2 VPLGDL**P**VVKLGDLVIFDEIPCLRYLAKKLGEYGRNYYIDFVIDDVIFRCSKWRDILMEL 177

C. hominis_TU502 GST2 VPLGDL**P**VVKLGDLVIFDEIPCLRYLAKKLGEYGRNYYIDFVIDDVIFRCSKWRDILMEL 177

C. parvum_Iowa II GST2 VPLGDL**P**VVKLGDLVIFDEIPCLRYLAKKLGEYGRNYYIDFVIDDVIFRCSKWRDILMEL 177

C. tyzzeri_UGA55 GST2 VPLGDL**P**VVKLGDLVIFDEIPCLRYLAKKLGEYGRNYYIDFVIDDVIFRCSKWRDILMEL 177

C. baileyi strain TAMU-09Q1 GST2 VPLGDL**P**IIKLGDLVLFDEIPCLRYLAKKLGEYGRNYYIDFVIDDIILRCSRWRDVIMEI 144

C. ryanae isolate 45019 GST2 VPLGDL**P**IIKLGDLVLFDEIPCLRYLAKKLGEYGRNYYVDFVIDDIILRCSRWRDIIMEL 144

C. *bovis* isolate 42482 GST2 VPLGDL**P**IVKLGDLVLFDEIPCLRYLAKKLGEYGRNYYVDFIIDDIILRCSRWRDIIMEL 129

****:**::******:********:*************:**:***:::**.:***::*::

C. andersoni 30847 GST2 ILSSNNCMLAASTNLDKSEAPYSLNNSENSGGSAISSLEGYKQLREQLYTEFEVLITSIG 235

C. ubiquitum_39726 GST2 ISRNYSELSNGN---------------INTNKELESSISNYKLLREQLYCEFETLIASIG 222

C. viatorum isolate UKVIA1 GST2 ISKSHKEFLNSN---------------INANNELEKSISNYKLLREQLYCEFETLIASIG 222

C. meleagridis_UKMEL1 GST2 ILKSHKEFLIND---------------INTNKELERLISNYKLLREQLYCEFETLISSIG 222

C. hominis_TU502_2012 GST2 ISKSHKEFLIND---------------INAKKELERSISNYKLLREQLYCEFETLISTIG 222

C. hominis_UdeA01 GST2 ISKSHKEFLIND---------------INAKKELERSISNYKLLREQLYCEFETLISTIG 222

C. hominis_30976 GST2 ISKSHKEFLIND---------------INAKKELERSISNYKLLREQLYCEFETLISTIG 222

C. hominis_TU502 GST2 ISKSHKEFLIND---------------INAKKELERSISNYKLLREQLYCEFETLISTIG 222

C. parvum_Iowa II GST2 ISKSRKEFLIND---------------INANKELERSTSNYKLLREQLYCEFETLILSIG 222

C. tyzzeri_UGA55 GST2 ISKSRKEFLINE---------------INANKELERSISNYKLLREQLYCEFETLISSIG 222

C. baileyi strain TAMU-09Q1 GST2 ISKGAAIVPNKN---------------S-V-QDHINSLSNYKVLREKFYSEFETLITCIG 187

C. ryanae isolate 45019 GST2 IPGSNTGVSAKS---------------Y-I--EETNSLSNYKLLREQFYYEFETLITCIG 186

C. *bovis* isolate 42482 GST2 ITENNTGSYSKN---------------H-VKGEEINPISNYKLLREQFYYEFETLITCIG 173

* . . . ..** ***::* ***.** **

C. andersoni 30847 GST2 EKEGSYIADKDKPMICDFALFSVLFDDINLSDISPDSMFQRIELLPDNCLIHQFPRLKSL 295

C. ubiquitum_39726 GST2 DK-GPFIAEKNKPMICDFILFSILFDDISLIEFSETEKFNRVTLLPERSIIHKFPRLKML 281

C. viatorum isolate UKVIA1 GST2 DK-GPFIAEKNKPMICDFILFSILFDDISLIEFNETEKFNRMKLLPEESIIHKFPRLKML 281

C. meleagridis_UKMEL1 GST2 DK-GPFIAEKNKPMICDFILFSILFDDISLIEFNEGEKLNRTSLLPEESIIHKFPRLKML 281

C. hominis_TU502_2012 GST2 DK-GPFIAEKNKPMICDFILFSILFDDISLIEFNEGEKINRTSLLPEESLIHKFPRLKML 281

C. hominis_UdeA01 GST2 DK-GPFIAEKNKPMICDFILFSILFDDISLIEFNEGEKINRTSLLPEESLIHKFPRLKML 281

C. hominis_30976 GST2 DK-GPFIAEKNKPMICDFILFSILFDDISLIEFNEGEKFNRTSLLPEESLIHKFPRLKML 281

C. hominis_TU502 GST2 DK-GPFIAEKNKPMICDFILFSILFDDISLIEFNEGEKFNRTSLLPEESLIHKFPRLKML 281

C. parvum_Iowa II GST2 DK-GPFIAEKNKPMICDFILFSILFDDISLIEFNEGEKFNRTSLLPEESLIHKFPRLKML 281

C. tyzzeri_UGA55 GST2 DK-GPFIAEKNKPMICDFILFSILFDDISLIEFNEGEKFNRTSLLPEESLIHKFPRLKML 281

C. baileyi strain TAMU-09Q1 GST2 ER-GPFIADKNKAMICDFALFSILFDDVSLMEINEHDQLNRTVLLPENCIIHKFPRLKLL 246

C. ryanae isolate 45019 GST2 DS-GIFIADSNRPMICDFILFSILFDDISLVEIDENNQFNRTTMIPENSIIHRFPRLKIL 245

C. *bovis* isolate 42482 GST2 ES-GIFIADGNKPMICDFILFSILFDDISLVEIDDNNQFNRTAMLPENSIIHRFPRLKIL 232

: * :**: :: ***** ***:****:.* ::. . ::* ::*:..:**:***** *

C. andersoni 30847 GST2 FLVMSELPLVNQWIKGKYFIQSNIDKATNKDSNENAAASTFPLQSSIVGNQQPSHSLYSL 355

C. ubiquitum_39726 GST2 FESVAILPLIDQWIKGKYFAIQIEGESSELVT---------PPTSLTTQDHG-------- 324

C. viatorum isolate UKVIA1 GST2 FESVATLPLIDQWVKGKYFDIQIEGENSELLT---------PPASLSTQDYA-------- 324

C. meleagridis_UKMEL1 GST2 FESVVMLPLIDQWVKGKYFSIQIEGESGELVT---------PPTSLSTQDHV-------- 324

C. hominis_TU502_2012 GST2 FESVAVLPLIDQWVKGKYFSIQIEGESGELVT---------PPASLSTQDHV-------- 324

C. hominis_UdeA01 GST2 FESVAVLPLIDQWVKGKYFSIQIEGESGELVT---------PPASLSTQDHV-------- 324

C. hominis_30976 GST2 FESVAVLPLIDQWVKGKYFSIQIEGESGELVT---------PPASLSTQDHV-------- 324

C. hominis_TU502 GST2 FESVAVLPLIDQWVKGKYFSIQIEGESGELVT---------PPASLSTQDHV-------- 324

C. parvum_Iowa II GST2 FESVAVLPLIDQWVKGKYFSIQIEGESGELVT---------PPASLSTQDHV-------- 324

C. tyzzeri_UGA55 GST2 FESVAVLPLIDQWVKGKYFSIQIEGESGELVT---------PPASLSTQDYV-------- 324

C. baileyi strain TAMU-09Q1 GST2 FESIAALPLIDQWIKGKYFIIEVENDNNDQVN---------LLTSNLLQESN-------- 289

C. ryanae isolate 45019 GST2 FESISSLPLIEQWIKGKYFLVNIESESNIGDL---------ITQKNSFPIM--------- 287

C. *bovis* isolate 42482 GST2 FESISSLPLIEQWIKGKYFIVNVEGEINAKDL---------TAQKNSFPIM--------- 274

* : ***::**:***** . .. .

C. andersoni 30847 GST2 GAGNGIASPGVFSIYQSSHPLNPPIPRFQYPMIPYMPNQGLVQASAGVRFAFPGAGLPIN 415

C. ubiquitum_39726 GST2 --------TNF-VVGTNSFIGCPNSFGYQPPVFQQLPNQLFAHVNAGIRFFPQNMAMPIN 375

C. viatorum isolate UKVIA1 GST2 --------TNF-VSGSNSFNGYQHSLGYQPPVFQQLPNQIFAHVNAGVRFFPQKMSLPIN 375

C. meleagridis_UKMEL1 GST2 --------RNS-VLGSNSFNWYQHSFGYQPPVPQQLPNQIFTHVSAGVRFFPQKVPLPIN 375

C. hominis_TU502_2012 GST2 --------KNS-VLGSNSFNVYQHSFGYQPPVLQQLPNQIFTHVNAGVRFFPQKMSLPIN 375

C. hominis_UdeA01 GST2 --------KNS-VLGSNSFNVYQHSFGYQPPVLQQLPNQIFTHVNAGVRFFPQKMSLPIN 375

C. hominis_30976 GST2 --------KNS-VLGSNSFNVYQHSFGYQPPVLQQLPNQIFTHVNAGVRFFPQKMSLPIN 375

C. hominis_TU502 GST2 --------KNS-VLGSNSFNVYQHSFGYQPPVLQQLPNQIFTHVNAGVRFFPQKMSLPIN 375

C. parvum_Iowa II GST2 --------KNS-VLGSNSFNAYQHSFGYQPPVLQQLPNQIFTHVNAGVRFFPQKMSLPIN 375

C. tyzzeri_UGA55 GST2 --------KSS-VLGSNSFNVYQHSFGYQPPVLQQLPNQIFTHVNAGVRFFPQKMSLPIN 375

C. baileyi strain TAMU-09Q1 GST2 --------QSLINGYNNAFVNYPPMLRFQPFP-FQHGNQIFAQANAGVRFFSPSTMPTNN 340

C. ryanae isolate 45019 GST2 ---------EHTNHFFKKEVGNNQSSRFLPPIFQQPPNQIFGQATAGIRFLHQPINHINN 338

C. *bovis* isolate 42482 GST2 ---------EHSNNFYRQDLEHSPLPRFSPPIFQQFPGQVFAQTTAGIRFVPQPFNHMNS 325

: .* : :..**:** .

C. andersoni 30847 GST2 NQQIPVIQANSSFINPHFAPQLNPSLIHPF--PIYQTNLGSP-CNRMSPSQSFT--- 466

C. ubiquitum_39726 GST2 QPIFS---PNNSFVSQPITNYY-PFLNNQIQNHGYLGGVSSPFVQRISPSQSFKLKF 428

C. viatorum isolate UKVIA1 GST2 QPIFS---PNNSFISQPIANYHHHFLNNQIQSHGYLGGVSSPFIQKASPR-SFKLKF 428

C. meleagridis_UKMEL1 GST2 P-TFP---TNNSFISQPITNNYHHFLNSQIQDHRYLGRASSPFIQRVSPSQSFKLEF 428

C. hominis_TU502_2012 GST2 P-IFP---TNNSFISQPITNNYHHFLNSQVQGHRYLGGVSSPFMQRVSPSQSFKLEF 428

C. hominis_UdeA01 GST2 P-IFP---TNNSFISQPITNNYHHFLNSQVQGHRYLGGVSSPFMQRVSPSQSFKLEF 428

C. hominis_30976 GST2 P-IFP---TNNSFISQPITNNYHHFLNSQVQGHRYLGGVSSPFMQRVSPSQSFKLEF 428

C. hominis_TU502 GST2 P-IFP---TNNSFISQPITNNYHHFLNSQVQGHRYLGGVSSPFMQRVSPSQSFKLEF 428

C. parvum_Iowa II GST2 PSIFP---TNNSFISQPITNNYHHFFNSQVQGHRYLGGVSSPFIQRVSPSQSFKLEF 429

C. tyzzeri_UGA55 GST2 QSIFP---TNNSFISQPITNNYHHFLNRQVQGHRYLGGVSSPFIQRVSPSQSFKLEF 429

C. baileyi strain TAMU-09Q1 GST2 YPIIP---SSNSFISHSFVNYHPHLWSNQFMGNYCSSGFSSPVQQKMSPAQSF---- 390

C. ryanae isolate 45019 GST2 LHRIS---PSNSFGLQ-PQFHAPQAWMHQINGNCYFNNI------------------ 373

C. *bovis* isolate 42482 GST2 FHRIS---PSNSFNLQ-PRSYAPQTWMHQINGSCYFNNITSPMQFRASPNPSFRM-- 376

: ..** .

**Group 3 (Psi class)**

C. baileyi strain TAMU-09Q1 GST3 -------------------------------PMYL**Y**TTKELDNTQILRSLMVVSSLPFYE 29

C. meleagridis_UKMEL1 GST3 MKSISLLASVFAFLALFSTSVESVKAKARIIPITF**Y**STKELDSNHLIRTVLVYSGLAFAE 60

C. ryanae isolate 45019 GST3 ---------------------------------MF**Y**TSKTIDNSHLIRTLLVLSGIPFNE 27

C. bovis isolate 42482 GST3 ---------------------------------MF**Y**TSKTLDSTHLIRTLLILSSLPFNE 27

:*::* :*..:::*:::: *.: * *

C. baileyi strain TAMU-09Q1 GST3 VRFTKDSEAKKFFFDKIKSLGYLTPSI**P**VLSDPETFNSYISTEEAISQYILLSYYKELYP 89

C. meleagridis_UKMEL1 GST3 TRFKKDSESQAKLFKEITKSGFLQPSI**P**MISDTGKNVQYLSTDEAVLNYIILSYNKELFS 120

C. ryanae isolate 45019 GST3 FRFKKNSPSLEEMFNSVVESGFLVPSI**P**MITDNEYSVKNISQEEAIIHYLILSYYPDLFP 87

C. bovis isolate 42482 GST3 FRFKKNSSSMEEMFSSIIESGFLNPTI**P**MISDNEYSVRNLSQDEAIVHYLVLSYYGELFQ 87

**.*:* : :*..: . *:* *:**:::* :* :**: :*::*** :*:

C. baileyi strain TAMU-09Q1 GST3 STISEYIYSIQAASLMTSYMKKLTNILSESITLPCTKILTLNDIKHLLNVLEKKRSESKS 149

C. meleagridis_UKMEL1 GST3 KNLLLHTISIQLSSIARSYIKKTTKILDSSKTLTCSKLLTNENIHQTLKVLNDTFASTEH 180

C. ryanae isolate 45019 GST3 KVISDYAISLQIGSAVRSYIQKVHKIIELSQKLVCEKLLTIDNINITLKLLDDKFIETGS 147

C. bovis isolate 42482 GST3 KSISDHAISLQIGSTVRSYISKVSGLLELSESLKCEKLLEIENVNVTLRLVNDRFTDTEY 147

. : : *:* .* **:.* ::. * .* * *:* :::: *.:::. .:

C. baileyi strain TAMU-09Q1 GST3 KYFYGEKYTYIDVSLYNLILFIENVSPGCVIRRYPSLTKLAFEFSQIPQVLAYERSPHFL 209

C. meleagridis_UKMEL1 GST3 KFLIGNKVSFNDLIAYNLILFIENVASGCVISNFKGLRELAFNISSIPQIAKFESSSYFM 240

C. ryanae isolate 45019 GST3 RFYFGGRYSYFDASVYTLILFVENISSGCITSNYEGLKAFSKEFSSISQISKFEKSSYFL 207

C. bovis isolate 42482 GST3 KFFYGGKYSYIDTVVYTLILFIENISNGCIISNFDGLRSFSKEFSSIPQISKFEKSSYFL 207

:: * : :: * *.****:**:: **: .: .* :: ::*.* *: :* * :*:

C. baileyi strain TAMU-09Q1 GST3 SLTIPGTRAFAKPINFVLMSKAFDTLS- 236

C. meleagridis_UKMEL1 GST3 SLLVPGTHTFAQRINFAHSSPMFLSLTS 268

C. ryanae isolate 45019 GST3 SLIVPGTTRFVKPIDFVSQAHES----- 230

C. bovis isolate 42482 GST3 SLLIPGTKEFVKPIDFVTQS-------- 227

** :*** *.: *:*. :

**Figure S1.** Multiple amino acid alignment of glutathione transferases (GSTs) from *Cryptosporidium* species. The conserved amino acids, active site tyrosine and the *cis-*proline are bold and underlined. The N-terminal region is highlighted in cyan, the C-terminal region is highlighted in green and the purple text indicates the overlapping region where the N- and C-terminal regions are shared.

**Table S2.** Prediction of transmembrane helices in glutathione transferase (GST) proteins of *Cryptosporidium* species and GSTs belonging to different classes. Prediction of transmembrane helices in GSTs were carried out using TMHMM - 2.0 ^16^

| *C. andersoni* 30847 GST1(cand_012830) | len=197 | ExpAA=2.35 | First60=0.01 | PredHel=0 | Topology=o |
| --- | --- | --- | --- | --- | --- |
| *C. hominis* TU502_2012 GST1(ChTU502y2012_407g2365/Q18145.1) | len=186 | ExpAA=1.21 | First60=0.00 | PredHel=0 | Topology=o |
| *C. hominis* 30976 GST1(GY17_00002363) | len=186 | ExpAA=1.21 | First60=0.00 | PredHel=0 | Topology=o |
| *C. hominis* TU502 GST1(XP_667744.1) | len=161 | ExpAA=2.65 | First60=0.00 | PredHel=0 | Topology=o |
| *C. hominis* UdeA01 GST1(CUV07467.1) | len=161 | ExpAA=2.65 | First60=0.00 | PredHel=0 | Topology=o |
| *C. meleagridis* UKMEL1 GST1(CmeUKMEL1_03350) | len=193 | ExpAA=1.11 | First60=0.01 | PredHel=0 | Topology=o |
| *C. parvum* Iowa II GST1(cgd7_4780) | len=186 | ExpAA=1.48 | First60=0.00 | PredHel=0 | Topology=o |
| *C. tyzzeri* UGA55 GST1(CTYZ_00001095) | len=186 | ExpAA=3.20 | First60=0.00 | PredHel=0 | Topology=o |
| *C. ubiquitum* 39726 GST1(cubi_03151) | len=213 | ExpAA=0.57 | First60=0.01 | PredHel=0 | Topology=o |
| *C. muris* RN66 GST1(XP_002141168.1) | len=160 | ExpAA=1.78 | First60=0.00 | PredHel=0 | Topology=o |
| *Cryptosporidium* sp. *chipmunk* LX-2015 *GST1 (JXRN01000042.1)* | len=205 | ExpAA=3.35 | First60=0.15 | PredHel=0 | Topology=o |
| *C. viatorum* isolate UKVIA1 GST1(QZWW01000010.1) | len=161 | ExpAA=1.92 | First60=0.00 | PredHel=0 | Topology=o |
| *C. baileyi* strain TAMU-09Q1 GST1(JIBL01000090.1) | len=156 | ExpAA=2.50 | First60=0.00 | PredHel=0 | Topology=o |
| *C. baileyi* strain TAMU-09Q1 GST2(JIBL01000106.1) | len=390 | ExpAA=0.01 | First60=0.01 | PredHel=0 | Topology=o |
| *C. viatorum* isolate UKVIA1 GST2(QZWW01000018.1) | len=428 | ExpAA=0.02 | First60=0.00 | PredHel=0 | Topology=o |
| *C. ryanae* isolate 45019 GST2(VHLK01000046.1) | len=373 | ExpAA=0.03 | First60=0.01 | PredHel=0 | Topology=o |
| *C. andersoni* 30847 GST2(cand_023790) | len=466 | ExpAA=0.03 | First60=0.00 | PredHel=0 | Topology=o |
| *C. hominis* TU502_2012 GST2(ChTU502y2012_421g0615) | len=428 | ExpAA=0.01 | First60=0.00 | PredHel=0 | Topology=o |
| *C. hominis* 30976 GST2(GY17_00000733) | len=428 | ExpAA=0.01 | First60=0.00 | PredHel=0 | Topology=o |
| *C. hominis* TU502 GST2(Chro.80347) | len=428 | ExpAA=0.01 | First60=0.00 | PredHel=0 | Topology=o |
| *C. hominis* UdeA01 GST2(CHUDEA8_2970) | len=428 | ExpAA=0.01 | First60=0.00 | PredHel=0 | Topology=o |
| *C. meleagridis* UKMEL1 GST2(CmeUKMEL1_14570) | len=428 | ExpAA=0.01 | First60=0.00 | PredHel=0 | Topology=o |
| *C. parvum* Iowa II GST2(cgd8_2970). | len=429 | ExpAA=0.01 | First60=0.00 | PredHel=0 | Topology=o |
| *C. tyzzeri* UGA55 GST2(CTYZ_0000322) | len=429 | ExpAA=0.01 | First60=0.00 | PredHel=0 | Topology=o |
| *C. ubiquitum* 39726 GST2(cubi_03523) | len=428 | ExpAA=0.01 | First60=0.00 | PredHel=0 | Topology=o |
| *C. bovis* isolate 42482 GST2(VHIT01000012.1) | len=376 | ExpAA=0.33 | First60=0.27 | PredHel=0 | Topology=o |
| *C. meleagridis* UKMEL1 GST3(CmeUKMEL1_05845) | len=268 | ExpAA=11.30 | First60=9.03 | PredHel=0 | Topology=o |
| *C. baileyi* strain TAMU-09Q1 GST3(JIBL01000138.1) | len=236 | ExpAA=0.49 | First60=0.19 | PredHel=0 | Topology=o |
| *C. ryanae* isolate 45019 GST3(VHLK01000056.1) | len=230 | ExpAA=0.76 | First60=0.19 | PredHel=0 | Topology=o |
| *C. bovis* isolate 42482 GST3(PRJNA545579) | len=227 | ExpAA=2.32 | First60=0.19 | PredHel=0 | Topology=o |

Abbreviations: len, the length of the protein sequence; ExpAA, the expected number of amino acids in transmembrane helices; First60, The expected number of amino acids in transmembrane helices in the first 60 amino acids of the protein; PredHel, the number of predicted transmembrane helices by N-best; Topology, the topology predicted by N-best.

**Table S3.** Comparative analysis of transmembrane helices in eukaryotic glutathione transferase. Prediction of transmembrane helices in GSTs were carried out using TMHMM - 2.0 ^16^. Abbreviation: PredHel, the number of predicted transmembrane helices.

| **GST Class** | **Location (experimental)** | **Number of GSTs analyzed** | **TMHMM 2.0 results** |
| --- | --- | --- | --- |
| Alpha | Cytosol | 37 | PredHel=0 |
| Beta | Cytosol | 6 | PredHel=0 |
| CLIC | Cytosol | 30 | PredHel=0 |
| Delta | Cytosol | 29 | PredHel=0 |
| Epsilon | Cytosol | 22 | PredHel=0 |
| Kappa | Mitochondrial | 5 | PredHel=0 |
| Lambda | Cytosol | 18 | PredHel=0 |
| MAPEG | Microsomal | 20 | PredHel=1-4 |
| Mu | Cytosol | 27 | PredHel=0 |
| Omega | Cytosol | 32 | PredHel=0 |
| Phi | Cytosol | 23 | PredHel=0 |
| Pi* | Cytosol | 33 | PredHel=0 |
| Sigma | Cytosol | 27 | PredHel=0 |
| Tau | Cytosol | 21 | PredHel=0 |
| Theta | Cytosol | 28 | PredHel=0 |
| Xi | Cytosol | 4 | PredHel=0 |
| Zeta | Cytosol | 33 | PredHel=0 |

*, only one GST from *Chlorocebus sabaeus* (protein ID: A0A0D9R8K4) showed one transmembrane helix.

**Table S4.** Analysis of *Cryptosporidium* species glutathione transferases (GSTs) cellular localization using Bologna Unified Subcellular Component Annotator (BUSCA) web-server^17^. All *Cryptosporidium* species GSTs predicated to be located in cytoplasm. The two GSTs transmembrane membrane helix scores are below the values (0.93 and 1 for transmembrane) that can be considered as membrane bound proteins GSTs.

| **GST** | **GO-id** | **GO-term** | **Score** | **Alternative localization** | **Features** |
| --- | --- | --- | --- | --- | --- |
| *C. muris* RN66 GST1(XP_002141168.1). | GO:0005737 | C:cytoplasm | 0.7 | GO:0005634 - C:nucleus (score=0.3) |  |
| *C. viatorum* isolate UKVIA1 GST1(QZWW01000010.1) | GO:0005737 | C:cytoplasm | 1 | - |  |
| *C. baileyi* strain TAMU-09Q1 GST1(JIBL01000090.1) | GO:0005737 | C:cytoplasm | 0.7 | GO:0005634 - C:nucleus (score=0.3) |  |
| *C. baileyi* strain TAMU-09Q1 GST2(JIBL01000106.1) | GO:0005737 | C:cytoplasm | 0.7 | GO:0005634 - C:nucleus (score=0.3) |  |
| *C. viatorum* isolate UKVIA1 GST2(QZWW01000018.1) | GO:0005737 | C:cytoplasm | 0.7 | GO:0005634 - C:nucleus (score=0.3) |  |
| *C. ryanae* isolate 45019 GST2(VHLK01000046.1) | GO:0005737 | C:cytoplasm | 0.7 | GO:0005634 - C:nucleus (score=0.3) |  |
| *C. andersoni* 30847 GST2(cand_023790) | GO:0005739 | C:mitochondrion | 0.64 | - | Mitochondrial Transit Peptide |
| *C. hominis*_TU502_2012 GST2(ChTU502y2012_421g0615) | GO:0005737 | C:cytoplasm | 0.7 | GO:0005634 - C:nucleus (score=0.3) |  |
| *C. hominis*_30976 GST2(GY17_00000733) | GO:0005737 | C:cytoplasm | 0.7 | GO:0005634 - C:nucleus (score=0.3) |  |
| *C. hominis*_TU502 GST2(Chro.80347) | GO:0005737 | C:cytoplasm | 0.7 | GO:0005634 - C:nucleus (score=0.3) |  |
| *C. andersoni* 30847 GST1(cand_012830) | GO:0012505 | C:endomembrane system | 0.73 | GO:0031090 - C:organelle membrane (score=0.67) | Transmembrane Alpha Helix |
| *C. hominis*_UdeA01 GST2(CHUDEA8_2970) | GO:0005737 | C:cytoplasm | 0.7 | GO:0005634 - C:nucleus (score=0.3) |  |
| *C. meleagridis*_UKMEL1 GST2(CmeUKMEL1_14570) | GO:0005737 | C:cytoplasm | 0.7 | GO:0005634 - C:nucleus (score=0.3) |  |
| *C. parvum_*Iowa II GST2(cgd8_2970) | GO:0005737 | C:cytoplasm | 0.7 | GO:0005634 - C:nucleus (score=0.3) |  |
| *C. tyzzeri*_UGA55 GST2(CTYZ_0000322) | GO:0005737 | C:cytoplasm | 0.7 | GO:0005634 - C:nucleus (score=0.3) |  |
| *C. ubiquitum*_39726 GST2(cubi_03523) | GO:0005737 | C:cytoplasm | 0.7 | GO:0005634 - C:nucleus (score=0.3) |  |
| *C. bovis* isolate 42482 GST2(VHIT01000012.1) | GO:0005737 | C:cytoplasm | 0.7 | GO:0005634 - C:nucleus (score=0.3) |  |
| *C. meleagridis*_UKMEL1 GST3(CmeUKMEL1_05845) | GO:0012505 | C:endomembrane system | 0.84 | GO:0005886 - C:plasma membrane (score=0.47) | Transmembrane Alpha Helix |
| *C. baileyi* strain TAMU-09Q1 GST3(JIBL01000138.1). | GO:0005737 | C:cytoplasm | 0.7 | GO:0005634 - C:nucleus (score=0.3) |  |
| *C. ryanae* isolate 45019 GST3(VHLK01000056.1) | GO:0005615 | C:extracellular space | 1 | - |  |
| *C. bovis* isolate 42482 GST3(PRJNA545579) | GO:0005615 | C:extracellular space | 1 | - |  |
| *C. hominis*_TU502_2012 GST1(ChTU502y2012_407g2365/Q18145.1) | GO:0005737 | C:cytoplasm | 0.89 | GO:0005634 - C:nucleus (score=0.11) |  |
| *C. hominis*_30976 GST1(GY17_00002363) | GO:0005737 | C:cytoplasm | 0.89 | GO:0005634 - C:nucleus (score=0.11) |  |
| *Cryptosporidium* sp. chipmunk *LX-2015 GST1 (JXRN01000042.1)* | GO:0005615 | C:extracellular space | 1 | - | - |
| *C. hominis* TU502 GST1(XP_667744.1) | GO:0005737 | C:cytoplasm | 1 | - |  |
| *C. hominis*_UdeA01 GST1(CUV07467.1) | GO:0005737 | C:cytoplasm | 1 | - |  |
| *C. meleagridis*_UKMEL1 GST1(CmeUKMEL1_03350) | GO:0005737 | C:cytoplasm | 1 | - |  |
| *C. parvum*_Iowa II GST1(cgd7_4780) | GO:0005737 | C:cytoplasm | 0.84 | GO:0005634 - C:nucleus (score=0.16) |  |
| *C. tyzzeri*_UGA55 GST1(CTYZ_00001095) | GO:0005737 | C:cytoplasm | 0.97 | - |  |
| *C. ubiquitum*_39726 GST1(cubi_03151) | GO:0005737 | C:cytoplasm | 1 | - |  |

**Table S5.** Information on template hits obtained from different databases for homology modeling of *Cryptosporidium* *parvum* GSTs 1 and 2 and C. meleagridis UKMEL1 GST3 (CmGST3). Yellow-shaded templates were found to be the best combination to generate good quality 3D models.

***C. parvum* GST1 (CpGST1) hits**

|  | **PDB-Chain** | **% Identity** | **Query Cover** | **Resolution** | **R-Value Free** | **R-Value Work** |
| --- | --- | --- | --- | --- | --- | --- |
| **NCBI** | 4L5O-A | 51 | 23 % | 2.09 | 0.248 | 0.202 |
|  | 3ISO-A | 51 | 23 % | 1.90 | 0.226 | 0.179 |
|  | 2WRT-A | 45 | 24 % | 2.40 | 0.281 | 0.205 |
|  | 1FHE-A | 45 | 24 % | 3.00 | 0.354 | 0.237 |
|  | 5AN1-A | 37 | 40 % | 2.00 | 0.262 | 0.214 |
|  | 1YQ1-A | 28 | 59 % | 3.00 | 0.297 | 0.224 |
| **PHYRE** | 1B8X-A | 27 | 98 % | 2.70 | 0.310 | 0.209 |
|  | 5AN1-A | 23 | 98 % | 2.00 | 0.262 | 0.214 |
|  | 1BG5-A | 27 | 98 % | 2.60 | 0.359 | 0.193 |
|  | 1GTU-B | 22 | 98 % | 2.68 | 0.245 | 0.211 |
|  | 1UA5-A | 25 | 98 % | 2.50 | 0.221 | 0.169 |
|  | 1C72-A | 21 | 98 % | 2.80 | 0.190 | 0.190 |
|  | 1VF1-A | 20 | 98 % | 1.77 | 0.217 | 0.192 |
|  | 4Q5Q-A | 20 | 98 % | 1.93 | 0.227 | 0.180 |
|  | 3C8E-B | 19 | 96 % | 2.71 | 0.181 | 0.182 |
|  | 4MZW-A | 19 | 97 % | 1.92 | 0.194 | 0.152 |
|  | 1GUM-A | 18 | 98 % | 3.00 | 0.270 | 0.253 |
|  | 5H5L-A | 18 | 98 % | 2.00 | 0.238 | 0.173 |
| **ITASSER** | 3VPQ-A | 26 | 94 % | 1.70 | 0.227 | 0.188 |
|  | 4Q5F-A | 22 | 94 % | 2.45 | 0.239 | 0.188 |
|  | 1M0U-A | 25 | 93 % | 1.75 | 0.232 | 0.213 |
|  | 4ZB9 | 22 | 98 % | 2.40 | 0.273 | 0.216 |
|  | 4ECI | 23 | 95 % | 1.80 | 0.209 | 0.175 |
|  | 5F05-A | 19 | 96 % | 1.70 | 0.181 | 0.144 |
|  | 4Q5F | 21 | 93 % | 2.45 | 0.239 | 0.188 |
|  | 5HFK-A | 26 | 97 % | 1.55 | 0.212 | 0.185 |
|  | 1DUG-A | 30 | 93 % | 1.80 | 0.226 | 0.185 |
|  | 1B8X-A | 29 | 95 % | 2.70 | 0.310 | 0.209 |

***C. parvum* GST2 (CpGST2) hits**

|  | **PDB-Chain** | **% Identity** | **Query cover** | **Resolution** | **R-Value Free** | **R-Value Work** |
| --- | --- | --- | --- | --- | --- | --- |
| **NCBI** | 5H5L-A | 26 | 73 % | 2.00 | 0.238 | 0.173 |
|  | 2WS2-A | 22 | 73 % | 2.01 | 0.290 | 0.236 |
|  | 2AAW-A | 24 | 88 % | 2.40 | 0.237 | 0.194 |
|  | 2ON7-A | 27 | 37 % | 2.40 | 0.280 | 0.190 |
|  | 1OKT-A | 24 | 89 % | 1.90 | 0.259 | 0.222 |
|  | 3W8S-A | 21 | 92 % | 1.90 | 0.259 | 0.222 |
|  | 1TW9-A | 20 | 92 % | 1.71 | 0.232 | 0.180 |
|  | 4ZXG-A | 24 | 86 % | 1.70 | 0.229 | 0.183 |
|  | 2ON5-A | 21 | 74 % | 1.90 | 0.227 | 0.178 |
|  | 3FR3-A | 22 | 86 % | 1.90 | 0.252 | 0.211 |
|  | 3FR6-A | 24 | 86% | 2.60 | 0.302 | 0.216 |
|  | 4KDU-A | 27 | 29% | 1.60 | 0.200 | 0.168 |
| **PHYRE** | 5H5L-A | 21 | 93 % | 2.00 | 0.238 | 0.173 |
|  | 1K3Y-B | 21 | 94 % | 1.30 | 0.207 | 0.137 |
|  | 1M0U-B | 19 | 93 % | 1.75 | 0.232 | 0.213 |
|  | 3VPT-A | 19 | 93 % | 1.90 | 0.253 | 0.204 |
|  | 1GTU-B | 19 | 94 % | 2.88 | 0.245 | 0.211 |
|  | 2WB9-A | 18 | 90 % | 1.59 | 0.222 | 0.175 |
|  | 3ISO-B | 18 | 96 % | 1.90 | 0.226 | 0.179 |
|  | 1B8X-A | 17 | 95 % | 2.70 | 0.310 | 0.209 |
|  | 1BG5-A | 17 | 95 % | 2.60 | 0.359 | 0.193 |
|  | 4W66-A | 17 | 95 % | 2.39 | 0.238 | 0.186 |
|  | 5AN1-A | 17 | 94 % | 2.00 | 0.262 | 0.214 |
| **ITASSER** | 3ISO-A | 20 | 86 % | 1.90 | 0.226 | 0.179 |
|  | 2ON5-A | 19 | 83 % | 1.90 | 0.227 | 0.178 |
|  | 1TU7-A | 21 | 83 % | 1.5 | 0.181 | 0.150 |
|  | 19GS-A | 22 | 84 % | 1.9 | 0.249 | 0.212 |
|  | 2ON7 | 19 | 83 % | 2.4 | 0.284 | 0.190 |
|  | 4Q5N-A | 19 | 88 % | 2.5 | 0.283 | 0.224 |
|  | 19GS | 22 | 80 % | 1.9 | 0.249 | 0.212 |
|  | 3GTU-B | 19 | 86 % | 2.80 | 0.270 | 0.225 |
|  | 5H5L-A | 23 | 79 % | 2.00 | 0.238 | 0.173 |
|  | 4W66-A | 18 | 84 % | 2.39 | 0.238 | 0.186 |

***C. meleagridis* UKMEL1 GST3 (C*m*GST3) hits**

|  | **PDB-Chain** | **% Identity** | **Query cover** | **Resolution** | **R-Value Free** | **R-Value Work** |
| --- | --- | --- | --- | --- | --- | --- |
| **NCBI** | 1HNA-A | 19 | 92 % | 1.85 | 0.226 | 0.226 |
|  | 1XW5-A | 19 | 92 % | 1.80 | 0.232 | 0.206 |
|  | 2C4J-A | 19 | 92 % | 1.35 | 0.213 | 0.194 |
|  | 2DC5-A | 19 | 92 % | 1.60 | 0.202 | 0.188 |
|  | 1GSU-A | 22 | 73 % | 1.94 | 0.292 | 0.210 |
|  | 4GTU-A | 19 | 92 % | 3.30 | 0.315 | 0.245 |
|  | 6KLD-A | 52% | 8 % | 3.58 | - | - |
|  | 6GSX-A | 17% | 98% | 1.91 | - | 0.52 |
| **PHYRE** | 1K3Y-B | 22 | 98 % | 1.30 | 0.207 | 0.137 |
|  | 1GUM-A | 22 | 97 % | 3.00 | 0.270 | 0.253 |
|  | 1UA5-A | 21 | 97 % | 2.50 | 0.221 | 0.169 |
|  | 1B8X-A | 20 | 97 % | 2.70 | 0.310 | 0.209 |
|  | 2FHE-A | 20 | 98 % | 2.30 | 0.234 | 0.183 |
|  | 1BG5-A | 20 | 99 % | 2.60 | 0.359 | 0.193 |
|  | 4W66-A | 19 | 97 % | 2.60 | 0.359 | 0.193 |
|  | 1C72-A | 19 | 89 % | 2.80 | 0.280 | 0.190 |
|  | 1GTU-B | 18 | 98 % | 2.68 | 0.245 | 0.211 |
|  | 4Q5Q-A | 18 | 97 % | 1.93 | 0.227 | 0.180 |
| **ITASSER** | 3ISO-A | 20 | 95 % | 1.90 | 0.226 | 0.179 |
|  | 4Q5F-A | 18 | 94 % | 2.45 | 0.239 | 0.188 |
|  | 2AAW-C | 22 | 94 % | 2.40 | 0.237 | 0.194 |
|  | 2ON7 | 19 | 93 % | 2.40 | 0.280 | 0.190 |
|  | 1ZL9 | 21 | 93 % | 2.01 | 0.209 | 0.151 |
|  | 1ZL9-A | 21 | 94 % | 2.01 | 0.209 | 0.151 |
|  | 4RI6 | 20 | 93 % | 1.52 | 0.183 | 0.151 |
|  | 1XW6-A | 20 | 94 % | 1.90 | 0.247 | 0.227 |
|  | 1GUL-A | 28 | 92 % | 2.70 | 0.260 | 0.248 |
|  | 1B8X-A | 24 | 92 % | 2.70 | 0.310 | 0.209 |

**Table S6.** Validation of glutathione transferases (GSTs) from *Cryptosporidium parvum* (C*p*GST1 and C*p*GST2) and *Cryptosporidium meleagridis* GST3 (C*m*GST3) protein models. For comparative analysis templates were also included in the study.

|  | 3VPQ | 5AN1 | *Cp*GST1 post refinement | 1K3Y | 19GS | *Cp*GST2 post refinement | 1ZL9 | *Cm*GST3 post refinement |
| --- | --- | --- | --- | --- | --- | --- | --- | --- |
| Verify | 91.63% | 87.67% | 87.98% | 88.69% | 100% | 83.40% | 95.17 % | 26.29 % |
| Errat | 95.8974 | 92.8571 | 85.6322 | 97.1831 | 99 | 85.9649 | 97.4874 | 82.9146 |
| Procheck errors | 1 | 2 | 3 | 2 | 0 | 3 | 0 | 2 |
| Procheck Warnings | 4 | 4 | 2 | 4 | 7 | 2 | 6 | 3 |
| Procheck pass | 4 | 3 | 3 | 3 | 2 | 3 | 3 | 3 |
| Z-score | -8.08 | -8.44 | -6.71 | -7.89 | -7.66 | -5.81 | -7.79 | -3.68 |

**Table S7.** Glutathione transferases (GSTs) from *Cryptosporidium parvum* (C*p*GST1 and C*p*GST2) and *Cryptosporidium meleagridis* GST3 (C*m*GST3) protein models assessment by Ramachandran Plot. For comparative analysis templates were also included in the study.

|  | 3VPQ | 5AN1 | CpGST1 post refinement | 1K3Y | 19GS | *Cp*GST2 post refinement | 1ZL9 | *Cm*GST3 post refinement |
| --- | --- | --- | --- | --- | --- | --- | --- | --- |
| Favoured Region | 165 (92.7%) | 176 (90.7%) | 155 (92.3%) | 182 (92.4%) | 167 (93.3%) | 199 (90.9%) | 167 (93%) | 185 (92%) |
| Additionally allowed region | 11 (6.2%) | 16 (8.2%) | 11 (6.5%) | 14 (7.1%) | 10 (5.6%) | 17 (7.8%) | 11 (6.1%) | 14 (7%) |
| Generously allowed region | 4 (1.1%) | 2 (1%) | 2 (1.2) | 1 (0.5%) | 2 (1.1%) | 3 (1.4 %) | 1 (0.6%) | 0 |
| Disallowed region | 0 | 0 | 0 | 0 | 0 | 0 | 0 | 2 (1%) |

**Reference**

1 Deponte, M. & Becker, K. Glutathione S‐transferase from malarial parasites: structural and functional aspects. *Methods in enzymology* **401**, 241-253 (2005).

2 Shehu, D., Abdullahi, N. & Alias, Z. Cytosolic Glutathione S-transferase in Bacteria: A Review. *Polish Journal of Environmental Studies* **28** (2019).

3 Board, P. G. & Menon, D. Glutathione transferases, regulators of cellular metabolism and physiology. *Biochimica et biophysica acta (bba)-general subjects* **1830**, 3267-3288 (2013).

4 Scian, M. *et al.* Comparison of epsilon-and delta-class glutathione S-transferases: the crystal structures of the glutathione S-transferases DmGSTE6 and DmGSTE7 from Drosophila melanogaster. *Acta Crystallographica Section D: Biological Crystallography* **71**, 2089-2098 (2015).

5 Udomsinprasert, R. *et al.* Identification, characterization and structure of a new Delta class glutathione transferase isoenzyme. *Biochemical Journal* **388**, 763-771 (2005).

6 Robinson, A., Huttley, G. A., Booth, H. S. & Philip, G. Modelling and bioinformatics studies of the human Kappa-class glutathione transferase predict a novel third glutathione transferase family with similarity to prokaryotic 2-hydroxychromene-2-carboxylate isomerases. *Biochemical Journal* **379**, 541-552 (2004).

7 Chronopoulou, E. *et al.* in *Glutathione in Plant Growth, Development, and Stress Tolerance* 195-213 (Springer, 2017).

8 Akil, F. *et al.* The role of xenobotic metabolism MGST1 gene polymorphism in colorectal cancer patients. *Acta Med Indones* **44**, 284-289 (2012).

9 Torres-Rivera, A. & Landa, A. Glutathione transferases from parasites: a biochemical view. *Acta Tropica* **105**, 99-112 (2008).

10 Wu, B. & Dong, D. Human cytosolic glutathione transferases: structure, function, and drug discovery. *Trends in pharmacological sciences* **33**, 656-668 (2012).

11 Munyampundu, J.-P., Xu, Y.-P. & Cai, X.-Z. Phi class of glutathione S-transferase gene superfamily widely exists in nonplant taxonomic groups. *Evolutionary Bioinformatics* **12**, EBO. S35909 (2016).

12 Prade, L., Huber, R., Manoharan, T. H., Fahl, W. E. & Reuter, W. Structures of class pi glutathione S-transferase from human placenta in complex with substrate, transition-state analogue and inhibitor. *Structure* **5**, 1287-1295 (1997).

13 Yang, G. *et al.* In planta characterization of a tau class glutathione S-transferase gene from Juglans regia (JrGSTTau1) involved in chilling tolerance. *Plant cell reports* **35**, 681-692 (2016).

14 Di Matteo, A. *et al.* Structural Characterization of the Xi Class Glutathione Transferase From the Haloalkaliphilic Archaeon Natrialba magadii. *Frontiers in microbiology* **10**, 9, doi:10.3389/fmicb.2019.00009 (2019).

15 BOARD, G. P., BAKER, T. R., CHELVANAYAGAM, G. & JERMIIN, S. L. Zeta, a novel class of glutathione transferases in a range of species from plants to humans. *Biochemical Journal* **328**, 929-935 (1997).

16 Möller, S., Croning, M. D. & Apweiler, R. Evaluation of methods for the prediction of membrane spanning regions. *Bioinformatics (Oxford, England)* **17**, 646-653 (2001).

17 Savojardo, C., Martelli, P. L., Fariselli, P., Profiti, G. & Casadio, R. BUSCA: an integrative web server to predict subcellular localization of proteins. *Nucleic acids research* **46**, W459-W466 (2018).
