# Supplementary material for: Comparative analyses and structural insights of new class glutathione transferases in Cryptosporidium species
Source: Sci Rep. 2020 Nov 23;10:20370. doi: 10.1038/s41598-020-77233-5 (PMC7683740; doi:10.1038/s41598-020-77233-5)
Supplement: Supplementary file 2 — Supplementary Dataset 1. [file 41598_2020_77233_MOESM2_ESM.docx]

**Comparative Analyses and Structural Insights of New Class Glutathione Transferases in *Cryptosporidium* Species**

Mbalenhle Sizamile Mfeka ^1^, José Martínez-Oyanedel ^2^, Wanping Chen ^3^, Ikechukwu Achilonu ^4^, Khajamohiddin Syed ^5^*, Thandeka Khoza ^1^*

^1^ Department of Biochemistry, School of Life Sciences, University of KwaZulu-Natal (Pietermaritzburg campus), Scottsville, 3209, KwaZulu-Natal, South Africa.

^2^ Laboratorio de Biofísica Molecular, Departamento de Bioquímica y Biología Molecular, Facultad de Ciencias Biológicas, Universidad de Concepción, Barrio Universitario S/N, Casilla 160_C, Concepción, Chile.

^3^ Department of Molecular Microbiology and Genetics, University of Göttingen, Göttingen 37077, Germany; chenwanping1@foxmail.com.

^4^ Protein Structure-Function Research Unit, School of Molecular and Cell Biology, University of the Witwatersrand, Braamfontein, Johannesburg, South Africa.

^5^ Department of Biochemistry and Microbiology, Faculty of Science and Agriculture, University of Zululand, 1 Main Road Vulindlea, KwaDlangezwa 3886, South Africa.

* Corresponding authors’ email:

khajamohiddinsyed@gmail.com and khozat1@ukzn.ac.za

***Cryptosporidium* species GSTs**

>C. andersoni 30847 GST1(cand_012830)

MNDSNYPYSVKSPLKLIYFACRGSCDVIRLLLNDKCIPYEEHNIQGKDFLQPEFQNVLLESDNFPILPYLSDPNSEMELTGSLTILRYLGRKCNLMGNNYEDELQIENWLEYLQLVLNILWEFDSNSDNFNNIQKNKKRGQFLLENLHPMLHNIQVRLDNGKKWIMEEYSVADIMLYTVVSAIIRSWGYEILQPYDK

>C. hominis_TU502_2012 GST1(ChTU502y2012_407g2365/Q18145.1)

MIEYLEHNISGKDFLQPEFQQVLVESGNFPMLPYFSDSNNEVELTGSFTILRYLADKCKLMGKSPEERNKIENWLEYLQSLLHSVWDFENMSDNYTGIQQAKKKSQFLLETLHPMLKCIDEKIEQGVWALESYSVVDIVLYSAISVIIRSWGSDLLKPYIRILTHKKNMEKLRKQIDSFKDDPRRF

>C. hominis_30976 GST1(GY17_00002363)

MIEYLEHNISGKDFLQPEFQQVLVESGNFPMLPYFSDSNNEVELTGSFTILRYLADKCKLMGKSPEERNKIENWLEYLQSLLHSVWDFENMSDNYTGIQQAKKKSQFLLETLHPMLKCIDEKIEQGVWALESYSVVDIVLYSAISVIIRSWGSDLLKPYIRILTHKKNMEKLRKQIDSFKDDPRRF

>C. hominis TU502 GST1(XP_667744.1)

MIEYLEHNISGKDFLQPEFQQVLVESGNFPMLPYFSDSNNEVELTGSFTILRYLADKCKLMGKSPEERNKIENWLEYLQSLLHSVWDFENMSDNYTGIQQAKKKSQFLLETLHPMLKCIDEKIEQGVWALESYSVVDIVLYSAISVIIRSWGSDLLKPYIR

>C. hominis_UdeA01 GST1(CUV07467.1)

MIEYLEHNISGKDFLQPEFQQVLVESGNFPMLPYFSDSNNEVELTGSFTILRYLADKCKLMGKSPEERNKIENWLEYLQSLLHSVWDFENMSDNYTGIQQAKKKSQFLLETLHPMLKCIDEKIEQGVWALESYSVVDIVLYSAISVIIRSWGSDLLKPYIR

>C. meleagridis_UKMEL1 GST1(CmeUKMEL1_03350)

MEYIGSLDNPLRLIYFSCRGTCDVIRLLLVDQEIPYEEHNISGKDFLQPEFQQVLLESGNFPMLPYFSDSNNEVELTGSFTILRYLADKCKLMGKSPEERNKIENWLEYLQSLLHSLWDFENMSDNYTGIQQTKKKRQFLLETLHPMLKCIDEKIEQGVWALESYSVVDIVLYSAISVVIRSWGSDLLKPYIR

>C. parvum_Iowa II GST1(cgd7_4780)

MIEYLEHNISGKDFLQPEFQQVLVESGNFPMLPYLSDSNNEVELTGSFTILRYLADKCKLMGKSPEERNKIENWLEYLQSLLHSVWDFENMSDNYTGIQQAKKKSQFLLETLHPMLKCIDEKIEQGIWALESYSVVDIVLYSAISVIIRSWGSDLLKPYIRILTHKKNMEKLRKQIDSFKDDPRRF

>C. tyzzeri_UGA55 GST1(CTYZ_00001095)

MIEYLEHNISGKDFLQPEFQQVLVESGNFPMLPYFSDSNNEVELTGSFTILRYLADKCKLMGKSPEERNKVENWLEYLQSLLHSVWDFENMSDNYTGIQQAKKKSQFLLETLHPMLKCIDEKIEQGVWALEYYSVVDIVLYSAISVIIRSWGSDLLKPYIRILTHKKNMEKLRKQIDSFKDDPRRF

>C. ubiquitum_39726 GST1(cubi_03151)

MEYIGSLDNPLRLIYFSCRGTCDAIRLLLVDQEIPYEGKDFLQPEFQHVLAESGNFPMLPYLSDSNNEVELTGSFTILRYLADKCKLMGNNSEERNRVENWLEFLQSLLHSVWDFENISENYTGVQQTKKKSKFLLDTLHPMLKCIDDKIEQGFWALESYSVVDIVLYSTISVVIRSWGSDLLKPYIRILSHKKNMEKLRKQIDSFKDDPRRF

>C. muris RN66 GST1(XP_002141168.1)

MINVFHMKGKDFLQSEFQNVLLESDNFPILPYLSDPNSEIELTGSLTILRYLGRKCNLMGNNYEDELQIENWFEYLQLVLNILWEFDSNLDSFNNIQKNKKRGQFLLENLHPMLHNIQARLDNGKKWILEEYSVADIMLYTVVSAIIRSWGYEILQPYDK

>C. viatorum isolate UKVIA1 GST1(QZWW01000010.1)

ILKYIEHNISGKDFLQPEFQQVLVESGNFPMLPYLSDSNNEVELTGSFTILRYLADKCKLMGKSPEERNKIENWLEYLQSLLHSVWDFENMSDNYTGIQHTKKKSQFLLETLHPMLKCIDEKIEQGVWALDSYSVVDVVLYSAISVVIRSWGIDLLKPYIK

>C. baileyi strain TAMU-09Q1 GST1(JIBL01000090.1)

EHNISGKDFLQEEFQQVLIESGNFPMLPYLSDSNNEVELTGSFTILRYLGEKCKLMGNNVTERNKIENWLEFLQSLLHSIWDFENNITNYTESQKKKRKSQFLLENLHPMLRSINDKIESNLWALNDYSIIDIVLYSTISVVIKLWSIDLLKPYEK

>Cryptosporidium sp. chipmunk LX-2015 GST1 (JXRN01000042.1)

ILYFRGTCDVIRLLLVDQEIPYEGKLKRYCQLIIYTVFSSGIQKFLNILEHNISGKDFLQPEFQQVLVESGNFPMLPYLSDSNNEVELTGSFTILRYLADKCKLMGKSPEERNKIENWLEYLQSLLHSVWDFENRSDNYTGVQQTKKRSQFLLETLHPMLKCIDEKIEHGVWVLDSYSVVDIVLYSAISVIIRSWGGDLLKPYTR

>C. baileyi strain TAMU-09Q1 GST2(JIBL01000106.1)

PMRSNVPCRLTNNRIMMPSKSTVKVVLPVRDIGELSVVTYEHDIFVGNGGSIRFFLLGKQVRHRFINVPLDEENPIPSFIDSSRVPLGDLPIIKLGDLVLFDEIPCLRYLAKKLGEYGRNYYIDFVIDDIILRCSRWRDVIMEIISKGAAIVPNKNSVQDHINSLSNYKVLREKFYSEFETLITCIGERGPFIADKNKAMICDFALFSILFDDVSLMEINEHDQLNRTVLLPENCIIHKFPRLKLLFESIAALPLIDQWIKGKYFIIEVENDNNDQVNLLTSNLLQESNQSLINGYNNAFVNYPPMLRFQPFPFQHGNQIFAQANAGVRFFSPSTMPTNNYPIIPSSNSFISHSFVNYHPHLWSNQFMGNYCSSGFSSPVQQKMSPAQSF

>C. viatorum isolate UKVIA1 GST2(QZWW01000018.1)

MNNIEASTTASPKIIAAKVSSELNEIYSPKMSKLVRNNIPCRLTTNRVMAPSRSTYRVILPVRDIGDLSVITYEHEVYVGNGGSLRFFLLGKQVRHRFINVHLDEEDPIPSYIDPNKVPLGDLPVVKLGDLVIFDEIPCLRYLAKKLGEYGRNYYIDFVIDDVIFRCSRWRDILMELISKSHKEFLNSNINANNELEKSISNYKLLREQLYCEFETLIASIGDKGPFIAEKNKPMICDFILFSILFDDISLIEFNETEKFNRMKLLPEESIIHKFPRLKMLFESVATLPLIDQWVKGKYFDIQIEGENSELLTPPASLSTQDYATNFVSGSNSFNGYQHSLGYQPPVFQQLPNQIFAHVNAGVRFFPQKMSLPINQPIFSPNNSFISQPIANYHHHFLNNQIQSHGYLGGVSSPFIQKASPRSFKLKF

>C. ryanae isolate 45019 GST2(VHLK01000046.1)

MSKTIIPFRLTSNRIMIPSRSAVRVVLPVRDIGELTVITFEHDIFVGNGGSIRFFLLGKQVRHRFVNVPLDEEKPIPSYIDSSRVPLGDLPIIKLGDLVLFDEIPCLRYLAKKLGEYGRNYYVDFVIDDIILRCSRWRDIIMELIPGSNTGVSAKSYIEETNSLSNYKLLREQFYYEFETLITCIGDSGIFIADSNRPMICDFILFSILFDDISLVEIDENNQFNRTTMIPENSIIHRFPRLKILFESISSLPLIEQWIKGKYFLVNIESESNIGDLITQKNSFPIMEHTNHFFKKEVGNNQSSRFLPPIFQQPPNQIFGQATAGIRFLHQPINHINNLHRISPSNSFGLQPQFHAPQAWMHQINGNCYFNNI

>C. andersoni 30847 GST2(cand_023790)

MIGVNSSISTGVASFSRDLSSLPGTSFIPAKAGSPQKSPSNLYGVIQAPRATSIRVMLPVRDIGDLTVVTYEHQAFVGCGGSLRFFLLGKQVKHKFINVPVDKDNPIPDYIESSKVPLGELPIIKLGDLVIFDEIPCLRFLAKKLGEYGRNYYIDFVIDDVVMRCNRWRDILMDLILSSNNCMLAASTNLDKSEAPYSLNNSENSGGSAISSLEGYKQLREQLYTEFEVLITSIGEKEGSYIADKDKPMICDFALFSVLFDDINLSDISPDSMFQRIELLPDNCLIHQFPRLKSLFLVMSELPLVNQWIKGKYFIQSNIDKATNKDSNENAAASTFPLQSSIVGNQQPSHSLYSLGAGNGIASPGVFSIYQSSHPLNPPIPRFQYPMIPYMPNQGLVQASAGVRFAFPGAGLPINNQQIPVIQANSSFINPHFAPQLNPSLIHPFPIYQTNLGSPCNRMSPSQSFT

>C. hominis_TU502_2012 GST2(ChTU502y2012_421g0615)

MNNKETSTIPSPKTIASKISSELSEIYSPKMSTLVRNNIPCRLTSNRVMAPSKSTYRVILPVRDIGDLSVITYEHEVYVGNGGSLRFFLLGKQVRHRFINVHLDEESPIPSYIDPNKVPLGDLPVVKLGDLVIFDEIPCLRYLAKKLGEYGRNYYIDFVIDDVIFRCSKWRDILMELISKSHKEFLINDINAKKELERSISNYKLLREQLYCEFETLISTIGDKGPFIAEKNKPMICDFILFSILFDDISLIEFNEGEKINRTSLLPEESLIHKFPRLKMLFESVAVLPLIDQWVKGKYFSIQIEGESGELVTPPASLSTQDHVKNSVLGSNSFNVYQHSFGYQPPVLQQLPNQIFTHVNAGVRFFPQKMSLPINPIFPTNNSFISQPITNNYHHFLNSQVQGHRYLGGVSSPFMQRVSPSQSFKLEF

>C. hominis_30976 GST2(GY17_00000733)

MNNKETSTIPSPKTIASKISSELSEIYSPKMSTLVRNNIPCRLTSNRVMAPSKSTYRVILPVRDIGDLSVITYEHEVYVGNGGSLRFFLLGKQVRHRFINVHLDEESPIPSYIDPNKVPLGDLPVVKLGDLVIFDEIPCLRYLAKKLGEYGRNYYIDFVIDDVIFRCSKWRDILMELISKSHKEFLINDINAKKELERSISNYKLLREQLYCEFETLISTIGDKGPFIAEKNKPMICDFILFSILFDDISLIEFNEGEKFNRTSLLPEESLIHKFPRLKMLFESVAVLPLIDQWVKGKYFSIQIEGESGELVTPPASLSTQDHVKNSVLGSNSFNVYQHSFGYQPPVLQQLPNQIFTHVNAGVRFFPQKMSLPINPIFPTNNSFISQPITNNYHHFLNSQVQGHRYLGGVSSPFMQRVSPSQSFKLEF

>C. hominis_TU502 GST2(Chro.80347)

MNNKETSTIPSPKTIASKISSELSEIYSPKMSTLVRNNIPCRLTSNRVMAPSKSTYRVILPVRDIGDLSVITYEHEVYVGNGGSLRFFLLGKQVRHRFINVHLDEESPIPSYIDPNKVPLGDLPVVKLGDLVIFDEIPCLRYLAKKLGEYGRNYYIDFVIDDVIFRCSKWRDILMELISKSHKEFLINDINAKKELERSISNYKLLREQLYCEFETLISTIGDKGPFIAEKNKPMICDFILFSILFDDISLIEFNEGEKFNRTSLLPEESLIHKFPRLKMLFESVAVLPLIDQWVKGKYFSIQIEGESGELVTPPASLSTQDHVKNSVLGSNSFNVYQHSFGYQPPVLQQLPNQIFTHVNAGVRFFPQKMSLPINPIFPTNNSFISQPITNNYHHFLNSQVQGHRYLGGVSSPFMQRVSPSQSFKLEF

>C. hominis_UdeA01 GST2(CHUDEA8_2970)

MNNKETSTIPSPKTIASKISSELSEIYSPKMSTLVRNNIPCRLTSNRVMAPSKSTYRVILPVRDIGDLSVITYEHEVYVGNGGSLRFFLLGKQVRHRFINVHLDEESPIPSYIDPNKVPLGDLPVVKLGDLVIFDEIPCLRYLAKKLGEYGRNYYIDFVIDDVIFRCSKWRDILMELISKSHKEFLINDINAKKELERSISNYKLLREQLYCEFETLISTIGDKGPFIAEKNKPMICDFILFSILFDDISLIEFNEGEKINRTSLLPEESLIHKFPRLKMLFESVAVLPLIDQWVKGKYFSIQIEGESGELVTPPASLSTQDHVKNSVLGSNSFNVYQHSFGYQPPVLQQLPNQIFTHVNAGVRFFPQKMSLPINPIFPTNNSFISQPITNNYHHFLNSQVQGHRYLGGVSSPFMQRVSPSQSFKLEF

>C. meleagridis_UKMEL1 GST2(CmeUKMEL1_14570)

MNSKETSTISSPKIIASKISSESSEIYSPKISTLTRNSIPCRLTSNRVMASSKSTYRVILPVRDIGDLSVITYEHEVYVGNGGSLRFFLLGKQVRHRFINVHLDEESPIPSYIDPNKVPLGDLPIVKLGDLVIFDEIPCLRYLAKKLGEYGRNYYIDFVIDDVIFRCSKWRDILMELILKSHKEFLINDINTNKELERLISNYKLLREQLYCEFETLISSIGDKGPFIAEKNKPMICDFILFSILFDDISLIEFNEGEKLNRTSLLPEESIIHKFPRLKMLFESVVMLPLIDQWVKGKYFSIQIEGESGELVTPPTSLSTQDHVRNSVLGSNSFNWYQHSFGYQPPVPQQLPNQIFTHVSAGVRFFPQKVPLPINPTFPTNNSFISQPITNNYHHFLNSQIQDHRYLGRASSPFIQRVSPSQSFKLEF

>C. parvum_Iowa II GST2(cgd8_2970)

MNNKETSTIPSPNIIASKISSELSEIYSPKMSTLVRNNIPCRLTSNRVMAPSKSTYRVILPVRDIGDLSVITYEHEVYVGNGGSLRFFLLGKQVRHRFINVHLDEESPIPSYIDPNKVPLGDLPVVKLGDLVIFDEIPCLRYLAKKLGEYGRNYYIDFVIDDVIFRCSKWRDILMELISKSRKEFLINDINANKELERSTSNYKLLREQLYCEFETLILSIGDKGPFIAEKNKPMICDFILFSILFDDISLIEFNEGEKFNRTSLLPEESLIHKFPRLKMLFESVAVLPLIDQWVKGKYFSIQIEGESGELVTPPASLSTQDHVKNSVLGSNSFNAYQHSFGYQPPVLQQLPNQIFTHVNAGVRFFPQKMSLPINPSIFPTNNSFISQPITNNYHHFFNSQVQGHRYLGGVSSPFIQRVSPSQSFKLEF

>C. tyzzeri_UGA55 GST2(CTYZ_0000322)

MNNKETSTIPSPKIIASKISSELSEIYSPKMSTLVRNNIPCRLTSNRVMAPSKSTYRVILPVRDIGDLSVITYEHEVYVGNGGSLRFFLLGKQVRHRFINVHLDEESPIPSYIDPNKVPLGDLPVVKLGDLVIFDEIPCLRYLAKKLGEYGRNYYIDFVIDDVIFRCSKWRDILMELISKSRKEFLINEINANKELERSISNYKLLREQLYCEFETLISSIGDKGPFIAEKNKPMICDFILFSILFDDISLIEFNEGEKFNRTSLLPEESLIHKFPRLKMLFESVAVLPLIDQWVKGKYFSIQIEGESGELVTPPASLSTQDYVKSSVLGSNSFNVYQHSFGYQPPVLQQLPNQIFTHVNAGVRFFPQKMSLPINQSIFPTNNSFISQPITNNYHHFLNRQVQGHRYLGGVSSPFIQRVSPSQSFKLEF

>C. ubiquitum_39726 GST2(cubi_03523)

MNNIGAGTTASPKNIATKVSSELNEIYSPKMSNLIRSNAPCRLTSNRVMIPSKSTYRVILPVRDIGDLSVITYEHEIYLGNGGSLRFFLLGKQVRHRFINVPLDEENPIPSYIDSDKVPLGDLPIVKLGDLVIFDEIPCLRYLAKKLGEYGRNYYIDFVIDDVIFRCSKWRDVLMDLISRNYSELSNGNINTNKELESSISNYKLLREQLYCEFETLIASIGDKGPFIAEKNKPMICDFILFSILFDDISLIEFSETEKFNRVTLLPERSIIHKFPRLKMLFESVAILPLIDQWIKGKYFAIQIEGESSELVTPPTSLTTQDHGTNFVVGTNSFIGCPNSFGYQPPVFQQLPNQLFAHVNAGIRFFPQNMAMPINQPIFSPNNSFVSQPITNYYPFLNNQIQNHGYLGGVSSPFVQRISPSQSFKLKF

>*C. bovis* isolate 42482 GST2(VHIT01000012.1)

MIPSRSAVRVVLPVRDIGELTVVTFEHNVFVGNGGSIRFFLLGKQVRHRFVNVPLDEEKPIPSYIDSSRVPLGDLPIVKLGDLVLFDEIPCLRYLAKKLGEYGRNYYVDFIIDDIILRCSRWRDIIMELITENNTGSYSKNHVKGEEINPISNYKLLREQFYYEFETLITCIGESGIFIADGNKPMICDFILFSILFDDISLVEIDDNNQFNRTAMLPENSIIHRFPRLKILFESISSLPLIEQWIKGKYFIVNVEGEINAKDLTAQKNSFPIMEHSNNFYRQDLEHSPLPRFSPPIFQQFPGQVFAQTTAGIRFVPQPFNHMNSFHRISPSNSFNLQPRSYAPQTWMHQINGSCYFNNITSPMQFRASPNPSFRM

>C. meleagridis_UKMEL1 GST3(CmeUKMEL1_05845)

MKSISLLASVFAFLALFSTSVESVKAKARIIPITFYSTKELDSNHLIRTVLVYSGLAFAETRFKKDSESQAKLFKEITKSGFLQPSIPMISDTGKNVQYLSTDEAVLNYIILSYNKELFSKNLLLHTISIQLSSIARSYIKKTTKILDSSKTLTCSKLLTNENIHQTLKVLNDTFASTEHKFLIGNKVSFNDLIAYNLILFIENVASGCVISNFKGLRELAFNISSIPQIAKFESSSYFMSLLVPGTHTFAQRINFAHSSPMFLSLTS

>C. baileyi strain TAMU-09Q1 GST3(JIBL01000138.1)

PMYLYTTKELDNTQILRSLMVVSSLPFYEVRFTKDSEAKKFFFDKIKSLGYLTPSIPVLSDPETFNSYISTEEAISQYILLSYYKELYPSTISEYIYSIQAASLMTSYMKKLTNILSESITLPCTKILTLNDIKHLLNVLEKKRSESKSKYFYGEKYTYIDVSLYNLILFIENVSPGCVIRRYPSLTKLAFEFSQIPQVLAYERSPHFLSLTIPGTRAFAKPINFVLMSKAFDTLS

>C. ryanae isolate 45019 GST3(VHLK01000056.1)

MFYTSKTIDNSHLIRTLLVLSGIPFNEFRFKKNSPSLEEMFNSVVESGFLVPSIPMITDNEYSVKNISQEEAIIHYLILSYYPDLFPKVISDYAISLQIGSAVRSYIQKVHKIIELSQKLVCEKLLTIDNINITLKLLDDKFIETGSRFYFGGRYSYFDASVYTLILFVENISSGCITSNYEGLKAFSKEFSSISQISKFEKSSYFLSLIVPGTTRFVKPIDFVSQAHES

>*C.* bovis isolate 42482 GST3(PRJNA545579)

MFYTSKTLDSTHLIRTLLILSSLPFNEFRFKKNSSSMEEMFSSIIESGFLNPTIPMISDNEYSVRNLSQDEAIVHYLVLSYYGELFQKSISDHAISLQIGSTVRSYISKVSGLLELSESLKCEKLLEIENVNVTLRLVNDRFTDTEYKFFYGGKYSYIDTVVYTLILFIENISNGCIISNFDGLRSFSKEFSSIPQISKFEKSSYFLSLLIPGTKEFVKPIDFVTQS

**Alpha Class GSTs**

>Bos Taurus(Q28035)

MAGKPTLHYFNGRGRMECIRWLLAAAGVEFEEKFIEKPEDLDKLKNDGSLMFQQVPMVEIDGMKLVQTRAILNYIATKYNLYGKDMKERALIDMYSEGVADLGEMIMHFPLCPPAEKDAKLTLIREKTTNRYLPAFENVLKSHGQDYLVGNKLSRADIHLVELLYYVEELDPSLLANFPLLKALKARVSNIPAVKKFLQPGSQRKPPTDEKKIEEARKVFKF

>Gallus gallus(P26697)

AKPVLYYFNGRGKMESIRWLLAAAGVEFEEVFLETREQYEKLLQSGILMFQQVPMVEIDGMKLVQTRAILNYIAGKYNLYGKDLKERALIDMYVGGTDDLMGFLLSFPFLSAEDKVKQCAFVVEKATSRYFPAYEKVLKDHGQDFLVGNRLSWADIHLLEAILMVEEKKSDALSGFPLLQAFKKRISSIPTIKKFLAPGSKRKPI

>Homo sapiens(P08263)

EKPKLHYFNARGRMESTRWLLAAAGVEFEEKFIKSAEDLDKLRNDGYLMFQQVPMVEIDGMKLVQTRAILNYIASKYNLYGKDIKERALIDMYIEGIADLGEMILLLPVCPPEEKDAKLALIKEKIKNRYFPAFEKVLKSHGQDYLVGNKLSRADIHLVELLYYVEELDSSLISSFPLLKALKTRISNLPTVKKFLQPGSPRKPP

>Rattus norvegicus(P00502)

GKPVLHYFNARGRMECIRWLLAAAGVEFDEKFIQSPEDLEKLKKDGNLMFDQVPMVEIDGMKLAQTRAILNYIATKYDLYGKDMKERALIDMYTEGILDLTEMIMQLVICPPDQKEAKTALAKDRTKNRYLPAFEKVLKSHGQDYLVGNRLTRVDIHLLELLLYVEEFDASLLTSFPLLKAFKSRISSLPNVKKFLQPGSQRKLPV

>Sus scrofa(P51781)

MAGKPILHYFNGRGRMECIRWLLAAAGVEFEEKFIKTPEDLDKLTNDGSLLFQQVPMVEIDGMKLVQTRAILNYIATKYNLYGKDAKERALIDMYTEGVADLGEMILLLPLCPPNEKDAKVASIKEKSTNRYLPAFEKVLKSHGQDYLVGNKLSRADIQLVELLYYVEELDPSLLANFPLLKALKTRVSNLPTVKKFLQPGSQRKPPMDAKKIRRSQEYFPD

>Gallus gallus(Q9W6J2)

MSGKPRLTYVNGRGRMESIRWLLSAAGVEFEEIFLETREQLLKLCQDGSLLFHQLPLVEIDGMKLVQCRAILSYIAGKYNLYGKDLKERALIDMYVEGISDLMQLILVFPFSPPEAKEKNLATIAEKATERYFPVFEKVLKQHGQDFLVGNRFSWADVQLMEAILAVEEKVPSVLSGFPQLQAFKTKMSNMPTIKKFLQPGSPRKPPPDEHYVATVKKIFKLN

>Homo sapiens(O15217)

MAARPKLHYPNGRGRMESVRWVLAAAGVEFDEEFLETKEQLYKLQDGNHLLFQQVPMVEIDGMKLVQTRSILHYIADKHNLFGKNLKERTLIDMYVEGTLDLLELLIMHPFLKPDDQQKEVVNMAQKAIIRYFPVFEKILRGHGQSFLVGNQLSLADVILLQTILALEEKIPNILSAFPFLQEYTVKLSNIPTIKRFLEPGSKKKPPPDEIYVRTVYNIFRP

>Homo sapiens(P09210)

MAEKPKLHYSNIRGRMESIRWLLAAAGVEFEEKFIKSAEDLDKLRNDGYLMFQQVPMVEIDGMKLVQTRAILNYIASKYNLYGKDIKEKALIDMYIEGIADLGEMILLLPFSQPEEQDAKLALIQEKTKNRYFPAFEKVLKSHGQDYLVGNKLSRADIHLVELLYYVEELDSSLISSFPLLKALKTRISNLPTVKKFLQPGSPRKPPMDEKSLEESRKIFRF

>Homo sapiens(Q7RTV2)

MAEKPKLHYSNARGSMESIRWLLAAAGVELEEKFLESAEDLDKLRNDGSLLFQQVPMVEIDGMKLVQTRAILNYIASKYNLYGKDMKERALIDMYTEGIVDLTEMILLLLICQPEERDAKTALVKEKIKNRYFPAFEKVLKSHRQDYLVGNKLSWADIHLVELFYYVEELDSSLISSFPLLKALKTRISNLPTVKKFLQPGSQRKPPMDEKSLEEARKIFRF

>Rattus norvegicus(P04903)

MSGKPVLHYFNARGRMECIRWLLAAAGVEFEEKLIQSPEDLEKLKKDGNLMFDQVPMVEIDGMKLAQTRAILNYIATKYDLYGKDMKERALIDMYSEGILDLTEMIIQLVICPPDQREAKTALAKDRTKNRYLPAFEKVLKSHGQDYLVGNRLTRVDIHLLELLLYVEEFDASLLTSFPLLKAFKSRISSLPNVKKFLQPGSQRKPAMDAKQIEEARKVFKF

>Rattus norvegicus(P04904)

MPGKPVLHYFDGRGRMEPIRWLLAAAGVEFEEQFLKTRDDLARLRNDGSLMFQQVPMVEIDGMKLVQTRAILNYIATKYNLYGKDMKERALIDMYAEGVADLDEIVLHYPYIPPGEKEASLAKIKDKARNRYFPAFEKVLKSHGQDYLVGNRLSRADVYLVQVLYHVEELDPSALANFPLLKALRTRVSNLPTVKKFLQPGSQRKPLEDEKCVESAVKIFS

>Mus musculus(P10648)

MAGKPVLHYFNARGRMECIRWLLAAAGVEFEEKFIQSPEDLEKLKKDGNLMFDQVPMVEIDGMKLVQTRAILNYIATKYDLYGKDMKERALIDMYTEGILDLTEMIGQLVLCPPDQREAKTALAKDRTKNRYLPAFEKVLKSHGQDYLVGNRLTRVDVHLLELLLYVEELDASLLTPFPLLKAFKSRISSLPNVKKFLHPGSQRKPPLDAKQIEEARKVFKF

>Mus musculus(P13745)

MAGKPVLHYFNARGRMECIRWLLAAAGVEFEEKFIQSPEDLEKLKKDGNLMFDQVPMVEIDGMKLAQTRAILNYIATKYDLYGKDMKERALIDMYSEGILDLTEMIGQLVLCPPDQREAKTALAKDRTKNRYLPAFEKVLKSHGQDYLVGNRLTRVDIHLLEVLLYVEEFDASLLTPFPLLKAFKSRISSLPNVKKFLQPGSQRKPPMDAKQIQEARKAFKIQ

>Rattus norvegicus(P14942)

MEVKPKLYYFQGRGRMESIRWLLATAGVEFEEEFLETREQYEKLQKDGCLLFGQVPLVEIDGMLLTQTRAILSYLAAKYNLYGKDLKERVRIDMYADGTQDLMMMIIGAPFKAPQEKEESLALAVKRAKNRYFPVFEKILKDHGEAFLVGNQLSWADIQLLEAILMVEEVSAPVLSDFPLLQAFKTRISNIPTIKKFLQPGSQRKPPPDGHYVDVVRTVLKF

>Antechinus stuartii(P80894)

MAGEQNIKYFNIKGRMEAIRWLLAVAGVEFEEKFFETKEQLQKLKETVLLFQQVPMVEIDGMKLVQTRAILHYIAEKYNLLGKDMKEHAQIIMYSEGTMDLMELIMIYPFLKGEEKKQRLVEIANKAKGRYFPAFENVLKTHGQNFLVGNQLSMADVQLFEAILMVEEKVPDALSGFPLLQAFKTRISNIPTVKTFLAPGSKRKPVPDAKYVEDIIKIFYF

>Cavia porcellus(P81706)

SGKPVLHYFNVQGRMESIRWLLAAAGVEFEEKLIMCQEDLDKLKNDGLLMFQQVPMVEMDGMKMVQSRAILNYIATKYNLYGKDTKERLLIDMYTEGMTDLYELFFKVILAPPEEKDAAKSLIKDRAKNRFLPAFEKVLKSHGQGYLVGNKLSKADILLTELLYMVEEFDASLLANFTLLQALKTRVSNLPNVKKFLQPGSQRKPFPTQEMFEEMRKF

>Gallus gallus(Q08393)

MAGKPKLHYTRGRGKMESIRWLLAAAGVEFEEEFIEKKEDLEKLRNDGSLLFQQVPMVEIDGMKMVQSRAILCYIAGKYNLYGKDLKERAWIDMYVEGTTDLMGMIMALPFQAADVKEKNIALITERATTRYFPVYEKALKDHGQDYLVGNKLSWADIHLLEAILMTEELKSDILSAFPLLQAFKGRMSNVPTIKKFLQPGSQRKPPLDEKSIANVRKIFSF

>Oryctolagus cuniculus(Q08863)

MARKPLLHYFNGRGRMESIRWLLAAAGEEFDEKFMETAEDLDKLRNDGSLMYQQVPMVEIDGMKLVQTRAILNYVANKHNLYGKDMKERALIDMYTEGVADLYELVLLLPLCPPEQKDAKVDFIKEKIRTRYFPAFEKVLKSHGQDYLVGNRLSKADILLVELLYNVEELDPSAIASFPLLKALKTRISSLPTVKKFLQPGSQRKPPMDEKNLEKAKKIFKIP

>Homo sapiens(Q16772)

MAGKPKLHYFNGRGRMEPIRWLLAAAGVEFEEKFIGSAEDLGKLRNDGSLMFQQVPMVEIDGMKLVQTRAILNYIASKYNLYGKDIKERALIDMYTEGMADLNEMILLLPLCRPEEKDAKIALIKEKTKSRYFPAFEKVLQSHGQDYLVGNKLSRADISLVELLYYVEELDSSLISNFPLLKALKTRISNLPTVKKFLQPGSPRKPPADAKALEEARKIFRF

>Bos Taurus(O18879)

GKPKLHYFNGRGRMECIRWLLAAAGVEFEEKFIEQPEDLDKLRNDGSLMFQQVPMVEIDGMKLVQTRAILNYIATKYNLYGKDMKERALIDMYSEGVEDLGEMIMHLPLCPPDQKDAKIAQIKERTTNRYFPAFEKVLKNHGQDYLVGNKLSKADIHLVELLYYVEELDPSLLANFPLLKGLKARVSSLPAVKKFLQPGSQRKPPM

>Rattus norvegicus(P46418)

GKPVLHYFDGRGRMEPIRWLLAAAGVEFEENFLKTRDDLARLRSDGSLMFEQVPMVEIDGMKLVQTKAILNYIATKYNLYGKDMKERALIDMYAEGVADLELMVLYYPYMPPGEKEASLAKIKDKARNRYFPAYEKVLKSHGQDYLVGNKLSRADVSLVELLYHVEEMDPGIVDNFPLLKALRTRVSNLPTVKKFLQPGSQRKPF

>Mus musculus(P24472)

AKPKLYYFNGRGRMESIRWLLAAAGVEFEEEFLETREQYEKMQKDGHLLFGQVPLVEIDGMMLTQTRAILSYLAAKYNLYGKDLKERVRIDMYADGTQDLMMMIAVAPFKTPKEKEESYDLILSRAKTRYFPVFEKILKDHGEAFLVGNQLSWADIQLLEAILMVEELSAPVLSDFPLLQAFKTRISNIPTIKKFLQPGSQRKPPP

>Rattus norvegicus(Q6AXY0)

MAEKPLFHYDEARGRMESVRWLLAAAGVEYEEKFIHTNEDLEKLRSDGVLMFQQVPMVEVDGMKLVQTRAIMNYFSSKYNLYGKDMKERALIDMYSEGLADLNEMFILYPFDPPGVKEANIALMKEKATNRYFPAFEKVFESHGQDYLVGNKLSKADVHLVEMIYNMEELDTNILANFPLLQALKTRISDMPTIKKFLQPGSQRQPPVDEKSIQKTRKIFKF

>Macaca mulatta(A0A023JCQ7)

MAGKPKLHYFNGRGRMEPIRWLLAAAGVEFEEKFIESAEDLGKLRNDGSLMFQQVPMVEIDGMKLVQTRAILNYIASKYNLYGKDIKERALIDMYTEGMADLNEMILLLPLCRPEEKDAKIALIKEKTKNRYFPAFEKVLQSHRQDYLVGNKLSRADISLVELLYYVEELDSSLISSFPLLKALKTRISNMPTVKKFLQPGSPRKPPPDAKALEEARKIFRF

>Canis lupus familiaris(A0A059V712)

MAVKPMLHYFNGRGRMESIRWLLASAGVEFEEKFINTPEDLDKLKNDGSLMFQQVPMVEIDGMKLVQTRAILNYIATKYNLYGKDIKERALIDMYTEGIVDLNEMIMVLPLCPPDQKDAKITLIRERTTDRYLPVFEKVLKSHGQDYLVGNKLSRADIHLVELLYYVEELDSSLLANFPLLKALKTRVSNLPTVKKFLQPGSPRKPPLDEKSLEQAKKIFRIN

>Aptenodytes forsteri(A0A087RJW0)

MAGKLKLYYFDGRGKMESIRWLLAAAGVEFEEEFLETREQYEKLLQGGSLLFQQLPMVEMDGMKMVQPRAILSYIAAKYNLYGKDLKERALIDMYVGGTDDLMGFILMFPFLSDEDKEKQRAVIVQKATSRYFPAYEKVLKDHGQDFLVGNNFSWADVHLLEAILMVEEKKSDVLSGFPQLQAFKARISSIPTIKKFLEPGSQRKPVPDDKYVETVKRVLRVYYDIKA

>Aptenodytes forsteri(A0A087RJW2)

MSGKPKLYYFNARGRMESIRWLLAAAGVEFEECFLETKDDLTKLRKDGSLLFQQVPMVEIDGMKMVQSRAIGNYIAMKYNLYGKDLKERALIDMYVEAVIDLNELLMTHPFQPADKKEQHFATIVDKATNRYFPVYEKVLKDHGQDFLVGNQFSRADVQLLETLLMAEECKPDILAKFPLLKSFKARISNIPTIKKFLQPGSQRKPPLQEKDVAKLMKIFH

>Poecilia formosa(A0A087XSY0)

MSGKVILHYFNGRGKMESIRWLLTVAEAEFDEYYLTDREQYLKLLNEGSLMFQQVPLVEIDGLKLVQTKAILHYIAEKYNLYGKDIKERAMINMYAEGLIDHMEMIMVLPFVTDTKPKLDNIQSKAKERYLPVFEKALTGPVYLVGGKLSLADVLLVECTLMLEEKFPDILKEFPNIKSFQGRMIRIPAISRFLQPGSKRKPQPDEDYVKTVKEVFNITGPFP

>Corvus brachyrhynchos(A0A091F0Y3)

MSGKPRLTYLNGRGRMEPIRWLLAAAGVEFEEVYLETKEQYDKLIKDGFLLFQQVPLVEIDGMKMVQTRAILSYIAGKYNLYGKDLKERALIDMYVEGIADLMQMILMFPFSPPDAKEKNLDSVKERATNRYFPVFEKVLKQHGQDFLVGNKFSWADVQLMEAIAFKTKMSNMPTIKKFLQPGSPRKPPPDAHYVETVLKVFKK

>Cuculus canorus(A0A091GFV5)

MAGKPKLYYFNGRGKMESVRWLLAAAGVEFEEEFLETQEQYEKLLQGGSLLFQQVPMVEIDGMKMVQTRAILSYIAAKYNLYGKDLKERALIDMYVGGTDDLMGFIMMFPFLSAEDKEKQRATIVQKATSRYFPAYEKILKDHGQNFLVGGSFSWADVHLLEAILMVEEKKSDVLSGFPQLQAFKARISSIPTIKKFLEPGSQRKPLPDDKYVETVRRVLRMYYDVKAN

>Cuculus canorus(A0A091GJB1)

MAAKPKLHYPKGRGKMESIRWLLAAAGVEFDEQFIEKKEDLEKIRNDGSLLFQQVPMVEIDGMKMVQTRAILSYIAAKYNLYGKDLKERAWIDMYVEGTTDLMGMIMSLPLQTAETKDKHLALIIERATTRYFPVYEKAFKTHGQDYLVGNKLSWADIQLLEAILMAEECKADILSAFPQLQAFKGRISNVPTIKKFLQPGSKRKPRPDEKYIAEMRKIFNF

>Buceros rhinoceros silvestris(A0A091H7X1)

MSGKPKLHYFNGRGRMESIRWLLAAAGVEFEECFLETKDDLIKLQKGGSLLFQQVPMVEIDGMKMVQTRAILNYIAAKYNLYGKDLKERALIDMYVEAIADLNELLMSHLFQPADKKEEHFATVVDKATNRYFPVYEKVLKDHGQDFLVGNQFSRADVQLLETLLMAEECKPDILARFPLLKSFKARISNIPTIKKFLQPGSQRKPPLQEEDIPKVMKIFH

>Calypte anna(A0A091I458)

MSGKPRLTYLNGRGRMEPVRWLLAAAGVEFEEVFLETREQYEKLIKDGVLMFQQVPLVEIDGMKMVQTRAILSYIAGKYNLYGKDLKERALIDMYVEGISDLMHMILMFHFSPPDAKEKNIDSVKDRATNRYFPVFEKVLKQHGQDFLVGNKFSWADVQLTEAILAVEEKIPAVLSEFPQLQAFKVRMTNMPTIKKFLQPGSPRKPPPDDHYVETVIKIFK

>Egretta garzetta(A0A091J816)

MSGKPKLHYFNGRGRMESIRWLLAAAGVEFEECFLETKDDLVKLQKDGSLLFQQVPMVEIDGMKMVQTRAISNYIATKYNLYGKDLKERALIDMYVEAVLDLNELLMTHAFQPADKKEQHFATIVDKATNRYFPVYEKVLKDHGQDFLVGNRFSRADVQLLETLLMAEECKPDILANFPLLQSFKARISNMPTIKKFLQPGSQRKPPLQEKDVPKLMKIFH

>Nestor notabilis(A0A091S0F7)

MSGKPRLTYLNGRGRMESIRWLLAAAGVEFEEIFLETKEQYEKIIKDGILMFQQVPLVEIDGMKMVQTRAILSYVAGKYNLYGKDLKERALIDMYVEGIIDLMQMILMFPFSPPEAKEKNLDSIKERATNRYFPVFEKVLKQHGQDFLVGNKFSWADVQLIEAILAVEEKVPAVLSGFPQLQVILLLSINMPTIKKFLQPGSPRKPPPDEHYVETVLKIFSK

>Mesitornis unicolor(A0A091SHE9)

MAGKPKLHYTKGRGKMESIRWLLAAAGVEFEEEFIEKKEDLDKLLKGGVLMFQQVPMVEIDGMKMVQTRAILSYIAGKYNLYGKDLKERAWIDMYVEGTTDLMGMIMVIPLQAADAKEKQLALIIERATTRYFPVYEKALKDHGHDYLVGNKLSWADIQLLEAILMTEECKPDILSAFPLLQAFKGRISNIPTIKKFLQPGSQRKPPPDDKYIASVRKIF

>Pelecanus crispus(A0A091SJF8)

MSGKPRLTYCNGRGRMEPVRWLLAAAGVEFEEIFLETREQYEKLIKDGVLMFQQVPLVEIDGMKMVQTRAILSYIAGKYNLYGKDLKERALIDMYVEGITDLMQMILMFPFSPPEAKEKNLDSIKERATNRYFPVFEKVLKQHGQEFLVGNKFSWADVQLIEAILAVEEKIPAVLSEFPQLQVI

**Beta Class GSTs**

>Escherichia coli(strain K12)(P0A9D2)

MKLFYKPGACSLASHITLRESGKDFTLVSVDLMKKRLENGDDYFAVNPKGQVPALLLDDGTLLTEGVAIMQYLADSVPDRQLLAPVNSISRYKTIEWLNYIATELHKGFTPLFRPDTPEEYKPTVRAQLEKKLQYVNEALKDEHWICGQRFTIADAYLFTVLRWAYAVKLNLEGLEHIAAFMQRMAERPEVQDALSAEGLK

>Proteus mirabilis(P15214)

MKLYYTPGSCSLSPHIVLRETGLDFSIERIDLRTKKTESGKDFLAINPKGQVPVLQLDNGDILTEGVAIVQYLADLKPDRNLIAPPKALERYHQIEWLNFLASEVHKGYSPLFSSDTPESYLPVVKNKLKSKFVYINDVLSKQKCVCGDHFTVADAYLFTLSQWAPHVALDLTDLSHLQDYLARIAQRPNVHSALVTEGLIKE

>Haemophilus influenzae(P44521)

MKLYGLIGACSFVPHVALEWVKIRENADYEFEPVTRELIKSPEFLSLNPRGAVPVLVDGDLVLSQNQAILHYLDELYPNSKLFGSKTVRDKAKAARWLAFFNSDVHKSFVPLFRLPNYAKDNETLAHTIRQQAVEQILDQLAVANEHLESHIYFGENISVADAYLYIMLNWCKAVKIDFSHLTQLSAFMQRVETDQAVENVRKSEELKV

>Xylella fastidiosa(Q9PE18)

MKLYIMPGACSLADHILLRWSGSSFDLQFLDHQSMKAPEYLALNPSGAVPALQVGDWVLTQNAAILNYITDIAPAERGLSGDGSLKARAEINRWIAFSNSDVHPMYWALFGGTAYLQDPQMIARSQDNARQKLRVLYQRADAHLKHHNWLANGQRSGADAYLYVTLRWAKKVGVDLSSLDALSAFFERMEADPGVQAALQAEGLI

>Xanthomonas campestris pv campestris(P45875)

MKLYTKPGACSLADHIVLRWSCLPFELTVVDAATMKSPDYLRLNPAGAVPLLVVDQWALTQNAAILNYIADTAPLTGLGGDGTARSRAEINRWIAFVNADLHPTFKPLFGSTAYLQEDALIQRSHEDARTKLRTLYTRVDAHLQGRNWLAGDTHTGADAYLFVTLRWAHKAGVDLSGLSALDAFFQRMLADADVQAALQAEGLN

>Ochrobactrum anthropi(P81065)

MKLYYKVGACSLAPHIILSEAGLPYELEAVDLKAKKTADGGDYFAVNPRGAVPALEVKPGTVITQNAAILQYIGDHSDVAAFKPAYGSIERARLQEALGFCSDLHAAFSGLFAPNLSEEARAGVIANINRRLGQLEAMLSDKNAYWLGDDFTQPDAYASVIIGWGVGQKLDLSAYPKALKLRERVLARPNVQKAFKEEGLN

**CLIC GSTs**

>Homo sapiens(O00299)

MAEEQPQVELFVKAGSDGAKIGNCPFSQRLFMVLWLKGVTFNVTTVDTKRRTETVQKLCPGGQLPFLLYGTEVHTDTNKIEEFLEAVLCPPRYPKLAALNPESNTAGLDIFAKFSAYIKNSNPALNDNLEKGLLKALKVLDNYLTSPLPEEVDETSAEDEGVSQRKFLDGNELTLADCNLLPKLHIVQVVCKKYRGFTIPEAFRGVHRYLSNAYAREEFASTCPDDEEIELAYEQVAKALK

>Homo sapiens(Q9NZA1)

CPFSQRLFMILWLKGVVFNVTTVDLKRKPADLHNLAPGTHPPFLTFNGDVKTDVNKIEEFLEETLTPEKYPKLAAKHRESNTAGIDIFSKFSAYIKNTKQQNNAALERGLTKALKKLDDYLNTPLPEEIDANTCGEDKGSRRKFLDGDELTLADCNLLPKLHVVKIVAKKYRNYDIPAEMTGLWRYLKNAYARDEFTNTCAADSEIELAYADVAKRL

>Rattus norvegicus(Q9EPT8)

CPFSQRLFMILWLKGVVFNVTTVDLKRKPADLHNLAPGTHPPFLTFNGDVKTDVNKIEEFLEETLTPEKYPKLAARHRESNTAGIDIFSKFSAYIKNTKQQNNAALERGLTKALRKLDDYLNTPLPEEIDTNTHGDEKGSQRKFLDGDELTLADCNLLPKLHVVKIVAKKYRNYDIPAEMTGLWRYLKNAYARDEFTNTCAADSEIELAYADVARRL

>Bos Taurus(P35526)

QRLFMILWLKGVVFNVTTVDLKRKPADLHNLAPGTHPPFLTFNGDVKTDVNKIEEFLEETLTPEKYPRLAAKHRESNTAGIDIFVKFSAYIKNTKQQSNAALERGLTKALKKLDDYLNTPLPEEIDADTRGDDEKGSRRKFLDGDELTLADCNLLPKLHVVKIVAKKYRNYDFPAEMTGLWRYLKNAYARDEFTNTCAADSEIELAYADVAKRL

>Homo sapiens(O15247)

MSGLRPGTQVDPEIELFVKAGSDGESIGNCPFCQRLFMILWLKGVKFNVTTVDMTRKPEELKDLAPGTNPPFLVYNKELKTDFIKIEEFLEQTLAPPRYPHLSPKYKESFDVGCNLFAKFSAYIKNTQKEANKNFEKSLLKEFKRLDDYLNTPLLDEIDPDSAEEPPVSRRLFLDGDQLTLADCSLLPKLNIIKVAAKKYRDFDIPAEFSGVWRYLHNAYAREEFTHTCPEDKEIENTYANVAKQKS

>Homo sapiens(O95833)

MAETKLQLFVKASEDGESVGHCPSCQRLFMVLLLKGVPFTLTTVDTRRSPDVLKDFAPGSQLPILLYDSDAKTDTLQIEDFLEETLGPPDFPSLAPRYRESNTAGNDVFHKFSAFIKNPVPAQDEALYQQLLRALARLDSYLRAPLEHELAGEPQLRESRRRFLDGDRLTLADCSLLPKLHIVDTVCAHFRQAPIPAELRGVRRYLDSAMQEKEFKYTCPHSAEILAAYRPAVHPR

>Rattus norvegicus(Q5M883)

MASLALNTQADPEIELFVKAGSDGESIGNCPFCQRLFMILWLKGVKFNVTTIDTARKPEELKDLAPGTNPPFLIYNKELKTDFIKIEEFLEKTLAPPRYPHLSPKYKESFDVGCNLFAKFSAYIKNTQKEANKNFEKSLLREFKRLDDYLNTPLLDEIDPDSTEERTLSRRLFLDGDQLTLADCSLLPKLNIIKVAAKKYRDFDIPAEFSGVWRYLHNAYAREEFAHTCPEDKEIENTYASVAKQ

>Bos taurus(Q5E9B7)

MAEEQPQVELFVKAGSDGAKIGNCPFSQRLFMVLWLKGVTFNVTTVDTKRRTETVQKLCPGGQLPFLLYGTEVHTDTNKIEEFLEAVLCPPRYPKLAALNPESNTAGLDIFAKFSAYIKNSNPALNDNLEKGLLKALKVLDNYLTSPLPDEVDETSAEDEGISQRKFLDGNELTLADCNLLPKLHIVQVVCKKYRGFSIPDVFRGVHRYLRNAYAREEFASTCPDDEEIELAYEQVAKALK

>Rattus norvegicus(Q6MG61)

MAEEQPQVELFVKAGSDGAKIGNCPFSQRLFMVLWLKGVTFNVTTVDTKRRTETVQKLCPGGQLPFLLYGTEVHTDTNKIEEFLEAVLCPPRYPKLAALNPESNTSGLDIFAKFSAYIKNSNPALNDNLEKGLLKALKVLDNYLTSPLPEEVDETSAEDEGISQRKFLDGNELTLADCNLLPKLHIVQVVCKKYRGFTIPEAFRGVHRYLSNAYAREEFASTCPDDEEIELAYEQVARALK

>Mus musculus(Q8BXK9)

MTDSATTNGDDRDPEIELFVKAGIDGESIGNCPFSQRLFMILWLKGVVFNVTTVDLKRKPADLHNLAPGTHPPFLTFNGDVKTDVNKIEEFLEETLTPEKYPKLAAKHRESNTAGIDIFSKFSAYIKNTKQQNNAALERGLTKALRKLDDYLNSPLPEEIDTNTHGDEKGSQRKFLDGDELTLADCNLLPKLHVVKIVAKKYRNYDIPAEMTGLWRYLKNAYARDEFTNTCAADSEIELAYADVARRLSRS

>Oryctolagus cuniculus(Q95MF9)

MAEEQPQVELFVKAGSDGAKIGNCPFSQRLFMVLWLKGVTFNVTTVDTKRRTETVHKLCPGGQLPFLLYGTEVHTDTNKIEEFLEAVLCPPRYPKLAALNPESNTAGVDIFAKFSAYIKNSNPALNDNLEKGLLKALKILDNYLTSPLPEEVDETSAEDEGISQRKFLDGNELTLADCNLLPKLHIVQVVCKKNRGFTIPEVFRGVHRYLSNAYAREEFASTCPDDEEIELAYEQVAKALK

>Mus musculus(Q9D7P7)

MAETTKLQLFVKASEDGESVGHCPSCQRLFMVLLLKGVPFTLTTVDTRRALDVLKDFAPGSQLPILLYDGDVKTDTLQIEEFLEETLGPPDFPSLAPRYRESNTAGNDIFHKFSAFIKNPVPTQDNALYQQLLRALTRLDSYLRAPLDHELAQEPHLRESHRRFLDGDQFTLADCSLLPKLHIVDTVCAHFRQLPIPAELSCVRRYLDSALQKKEFKYTCPHSAEILAAYQPAVHPR

>Mus musculus(Q9Z1Q5)

MAEEQPQVELFVKAGSDGAKIGNCPFSQRLFMVLWLKGVTFNVTTVDTKRRTETVQKLCPGGQLPFLLYGTEVHTDTNKIEEFLEAMLCPPRYPKLAALNPESNTSGLDIFAKFSAYIKNSNPALNDNLEKGLLKALKVLDNYLTSPLPEEVDETSAEDEGISQRKFLDGNELTLADCNLLPKLHIVQVVCKKYRGFTIPEAFRGVHRYLSNAYAREEFASTCPDDEEIELAYEQVARALK

>Oncorhynchus mykiss(A0A060WAT0)

MSLSVPQNGIKADNEPVIELFVKAGSDGESIGNCPFSQRLFMILWLKGVVFNVTTVDLKRKPADLQNLAPGTHPPFITFNGEVKTDVNKIEEFLEDVLSPPKFTKLGTRHPESNTAGMDIFAKFSAFIKNSKPDANEGLERGLLKTLQKLDEYLRSPLPDEIDHNSIEDIKISTRKFLDGDEMTLADCNLLPKLHIVKVVTKKYRGFDIPKDMTGIWQYLQNVYTREEFTNTCPSDKEIEIAYQDVAKRLVK

>Oncorhynchus mykiss(A0A060XCT7)

MSLSVPHNGIKADNEPVIELFVKAGSDGESIGNCPFSQRLFMILWLKGVVFNVTTVDLKRKPADLQNLAPGTHPPFITFNGEVKTDVNKIEEFLEDVLSPPKFTKLSARHPESNTAGMDIFAKFSAFIKNSKPNANEGLERGLLKTLQKLDEYLRSPLPDEIDHNSIEDVKNSTRKFLDGDNMTLADCNLLPKLHIVKVVTKKYRGFDIPKDMIGIWQYLQNAYTHEEFTNTCPSDKEIEIAYQDVAKRLIK

>Oncorhynchus mykiss(A0A060XHL8)

MAQRQNSDKDPTIELFIKAGHDGENMGNCPFCQRLFMVLWLKGVKFTVTTVDMRKKPAELKDLAPGTNPPFLLYNGTLKTDFIKIEEFLEQTLAPPRYPHLSPLSKESFDVGADIFAKFSAFIKNRPANSTFHEKALLREFKRLDLYLTSPIPEEINQNSRENILVSKRKFLDGNHLTLADCNLLPKLHVIKIAAKKYCDFDIPVQFTGVWRYLNNAYEREEFRQTCPANIEIEKAYLDVANKRL

>Aptenodytes forsteri(A0A087R950)

AGSDGESIGNCPFSQRLFMILWLKGVIFNVTTVDLKRKPADLQNLAPGTNPPFMTFDGEVKTDVNKIEEFLEEKLAPPRYPKLAPNHPESNSAGNDVFAKFSAFIKNPRKDANENLEKSLLKALRKLDNYLNSPLPDEIDAYSTEEITVSSRKFLDGDELTLADCNLLPKLHIIKVVAKKYRNFDFPPEMTGISRYLNNAYARDEFTNTCPADQEIEYAYLDVAKRMK

>Aptenodytes forsteri(A0A087QW98)

LLQAGSDGESIGNCPFSQRLFMILWLKGVVFSVTTVDLKRKPADLQNLAPGTHPPFITYNGEVKTDVNKIEEFLEDVLAPPKYLKLSPKHPESNTAGMDIFAKFSAFIKNSRPEANEALERGLLKTLQKLDEYLNSPLPDEIDENSMEDITISTRKFLDGNEMTLADCNLLPKLHIVKVVAKKYRDFEIPKEMTGIWRYLTNAYSRDEFTNTCPGDKEIEIAYSDVAKRLTK

>Balearica regulorum gibbericeps(A0A087V2T6)

QAGIDGESIGNCPFSQRLFMILWLKGVVFNVTTVDLKRKPADLHNLAPGTHPPFLTFNGEVKTDVNKIEEFLEEILAPPKYPTLAAKHRESNTAGIDIFSKFSAYIKNTKQQDNAALERGLVKALKKLDDYLRTPLPEEIDANSTEEEKVSKRKFLDGDDLTLADCNLLPKLHVVKIVAKKYRNFEFPTEMTGLWRYLKNAYARDEFTNTCAADKEIEQAYADVAKRLSKS

>Balearica regulorum gibbericeps(A0A087VFB6)

AGSDGESIGNCPFSQRLFMILWLKGVIFNVTTVDLKRKPADLQNLAPGTNPPFMTFDGEVKTDVNKIEEFLEEKLAPPRYPKLAPNHPESNSAGNDVFAKFSAFIKNPRKDANENLEKSLLKALRKLDNYLNSPLPDEIDAYSTEEITVSSRKFLDGDELTLADCNLLPKLHIIKVVAKKYRNFDFPPEMTGISRYLNNAYARDEFTNTCPADQEIEYAYLDVAKRMK

>Poecilia formosa(A0A087X883)

MSDANEPKIELFVKAGSDGLCIGNCPFSQRLFMVLWLKGVVFDVTTVDMRKKPDILNDLAPGAQPPFLQYGSEVKTDTNKIEEFIESTLCPPKYPRLAARNPESNTAGVDIFSKFSAYIKNSNPQMNDNLEKGLMKALQKLDNYLGSPLPDEIDQDAAEEATSSSRPFLDGQQLTLADCNLLPKLHILKVVSLKYRNFTIPESLTNVWRYLNAAYAREEFSATCPVDTEILMAYSTVAKALK

>Poecilia formosa(A0A087XDI2)

MSLSVPQNGVKADNEPVIELFVKAGSDGESIGNCPFSQRLFMILWLKGVVFNVTTVDLKRKPADLQNLAPGTHPPFITFNGEVKTDVNKIEEFLEDVLSPPKYVKLGAKHPESNTAGMDIFAKFSAYIKNSKPDANEALERGLLKTLQKLDDYLRSPLPDEIDHNSIEDIKFSSRKFLDGDEMTLADCNLLPKLHIVKVVTKKYRGFDIPKEMTSIWKYLNNAYTREEFTNTCPSDKEIEIAYGDVAKRLVK

>Fukomys damarensis(A0A091CT16)

MVLWLKGVTFNVTTVDTKRRTETVQKLCPGGQLPFLLYGTEVHTDTNKIEEFLEAMLCPPRYPKLAALNPESNTAGLDVFAKFSAYIKNSNPALNDNLEKGLLKALKVLDNYLISPLPEEVDETSAEDEGISRRKFLDGNELTLADCNLLPKLHIVQVVCKKYRGFTIPEAFQGVHRYLSNAYAREEFASTCPDDEEIELAYEQVAKALK

>Fukomys damarensis(A0A091EKZ0)

MTDTVTANGDDRDPEIELFVKAGIDGESIGNCPFSQRLFMILWLKGVVFNVTTVDLKRKPADLHNLAPGTHPPFLTFNGEVKTDVNKIEEFLEETLTPEKYPKLAAKHRESNTAGIDIFSKFSAYIKNTKQQNNAALERGLTKTLRKLDDYLSNPLPEEINTDTCGDSNRGSRRKFLDGDELTLADCNLLPKLHVVKIVAKKYRNYDFPAEMTGLWRYLKNAYARDEFTNTCAADSEIELAYADVAKRLSRS

>Corvus brachyrhynchos(A0A091F2P6)

AGSDGESIGNCPFSQRLFMILWLKGVIFNVTTVDLKRKPADLQNLAPGTNPPFMTFDGEVKTDVNKIEEFLEEKLAPPRYPKLAPKHPESNSAGNDVFAKFSAFIKNPRKDANENLEKSLLKALKKLDNYLNSPLPDEIDAYSTEEITVSSRKFLDGDELTLADCNLLPKLHIIKVVAKKYRNFHFPPEMTGISRYLKNAYARDEFTNTCPADQEIEYAYLDVAKRMK

>Corvus brachyrhynchos(A0A091F5A3)

QAGIDGESIGNCPFSQRLFMILWLKGVVFNVTTVDLKRKPADLHNLAPGTHPPFLTFNGEVKTDVNKIEEFLEEILAPPKYPKLAAKHRESNTAGIDIFSKFSAYIKNTKQQDNAALERGLVKALKKLDDYLRTPLPEEIDADSTEEEKVSKRKFLDGDDLTLADCNLLPKLHVVKIVAKKYRNFEFPAEMTGLWRYLKNAYARDEFTNTCAADKEIEQAYADVAKRLSKS

>Cuculus canorus(A0A091FUZ4)

QAGIDGESIGNCPFSQRLFMILWLKGVVFNVTTVDLKRKPADLHNLAPGTHPPFLTFNGEVKTDVNKIEEFLEETLAPPKYPKLAAKHRESNTAGIDIFSKFSAYIKNTKQQDNAALERGLVKALKKLDDYLRTPLPEEIDADSTEEEKVSKRKFLDGDDLTLADCNLLPKLHVVKIVTKKYRNFEFPTEMTGLWRYLKNAYTRDEFTNTCAADKEIEQAYADVAKRLSKS

>Buceros rhinoceros silvestris(A0A091GQ71)

QAGLDGENIGNCPFCQRLFMVLWLKGVKFNVTTVDMTRKPEELKDLAPGTNPPFLLFNKELKTDFIKIEEFLEQTLGPPMYPHLSPKYKESFDVGSDIFAKFSAYIKNPRKEANINFEKALLREFQRLDDSMEDITVSKRKFLDGDHLTLADCNLLPKLHIIKIAAKKYRDFEIPADMTGVWRYLNNAYACDEFSHTCPADEEI

>Egretta garzetta(A0A091JA73)

QAGIDGESIGNCPFSQRLFMILWLKGVVFNVTTVDLKRKPADLHNLAPGTHPPFLTFNGEVKTDVNKIEEFLEEILAPPKYPKLAAKHRESNTAGIDIFSKFSAYIKNTKQQDNAALERGLVKALKKLDDYLRTPLPEEIDADSTEEEKVSKRKFLDGDDLTLADCNLLPKLHVVKIVAKKYRNFEFPTEMTGLWRYLKNAYARDEFTNTCAADKEIEQAYADVAKRLSKS

>Calypte anna(A0A091J0Y9)

QAGIDGESIGNCPFSQRLFMILWLKGVVFNVTTVDLKRKPADLHNLAPGTHPPFLTFNGEVKTDVNKIEEFLEETLAPPKYPKLAAKHREANTAGIDIFSKFSAYIKNTKQQDNAALERGLVKALKKLDDYLRTPLPEEIDADSTEEEKVSKRKFLDGDDLTLADCNLLPKLHVVKIVTKKYRNFEFPTEMTGLWRYLRNAYARDEFTNTCAADKEIEQAYADVAKRLSKS

**Delta Class GSTs**

>Sarcoptes scabiei(Q5ISV1)

MASEKPTIYWMPESAPCRSVYLVAKILGIDCEWKVFNLFKKEQMNPEFLAINPLHCVPTLVESDGFTLWESRVICSYLIESRDPDSALYPKDLKKRAIIDRCLHFDLGTLYRALADVVYDILFFGKPNLTKLPRLEEVLQLMEDGFAKIDSDYLAGGDGPTLADIVSYFTLQMLDILQELDLTKYSKLYAWRERMEEFVKSNDDGSLAKGLQNFVGFAQQMQQQHSS

>Drosophila melanogaster(P20432)

MVDFYYLPGSSPCRSVIMTAKAVGVELNKKLLNLQAGEHLKPEFLKINPQHTIPTLVDNGFALWESRAIQVYLVEKYGKTDSLYPKCPKKRAVINQRLYFDMGTLYQSFANYYYPQVFAKAPADPEAFKKIEAAFEFLNTFLEGQDYAAGDSLTVADIALVATVSTFEVAKFEISKYANVNRWYENAKKVTPGWEENWAGCLEFKKYF

>Blattella germanica(A9QUN5)

MTIDFYYLPGSAPCRSVLLAAKAFGVNLNLKVTNLMAGEHLTPEFLKMNPQHTIPTLNDNGFCLWESRAILSYLADQYGKDDSLYPKDPKKRALVDQRLYFDLGTLYQRFGDYYYPIMFAKASPDAEKMKKLEEAYQFLDKFLEGQKFVAGNSLTIADIATIASVSTAAILGFDITRYPNVNKWFENAKKVIPGYDELNHSGCLEFRKMWDNLTQK

>Agrotis ipsilon(A0A2S0RQT2)

MPIDLYYVPGSAPCRAVLLTAKALNLNLNLKLVDLHHGEHLKPEYLKLNPQHTVPTLVDDGLSIWESRAIITYLVNKYGKGSSLYPEEPRARALVDQRLYFDIGTLYQRFADYFYPQVFGGAPADKEKLAKIEDALQLLNTFLEGQKFCAGPNLTVADLSLIAGVSSFEASDIDFKKYPNIKRWYETVKTTAPGYQEANEKGLDAFKGL

>Aedes aegypti(J9HHL7)

MDFYYLPGSAPCRAVQMTAAAVGVELNLKLTNLMAGEHMKPEFLKLNPQHCIPTLVDNGFSLWESRAIIAYLVEKYGKDDKLYPKDPQKRAVVNQRLYFDQGTLYQRFADYFYPQVFAKQAPVPDNEKKMLDALDFLNTFLKDSKYVAGDELTIADLSILATVSTFDVAKVDLSKYPNVASWYERLRKEAPGADINEAGCKEFAKYF

>Sarcoptes scabiei(Q2YFE6)

MAKPTFYYMPESPPCRTVMAVARMIGLDMEMKKLNLRNKEHLTPEFLKINPMHKVPTLVEPDGFALGESRAISTYIIQKYKPSSPLYPVDDLRRRAHIDGWLQYDCSTLGPALRAVIMDRMYGGGLNENRLNQTKETLKTLNEVLKALEGRYLLDDQITVADISMYFSCNMIEVLPDLEMSDYEHLCKWYKNMTEAMNAVDHDGLFAEAIQSAKKYIAEKL

>Danaus plexippus(A0A212EIY8)

MPSQPIKLYYLPPSPPCRAVMMTARALGLDLDLVLTNIMEGQHMTPEFLKMNPQHTIPTMDDSGFILWESRAIMTYLANAYGRDDSLYPKNPRSRALVDQRLNFDLGTLFNRFFNLYGRMLFQGEKYDDEAAKKLKEAIGWMNTMLDGRAFVAGDNMTLADISIIVTFSNLE

>Aedes aegypti(Q17MB7)

MDLYYHIIPPPSRAVLVLAKKLNITLNLISIDTRDANEMAILTEVNPLQSLPTLIDDGQVIGESHTVLIHLTSLFDKEGMLYPADLKIRSAINELLFFDTNMYKCFVLFAMPTVIKRQDPNHDMLEKLLVCVKALDNYLRARVYAAGDHFTLADLSLAHTISSLDVIKVKLSDYPNVERWMTKVLPEMPQFEEFQVRAEEALSTFLAKQYGAKCI

>Aedes aegypti(Q17MB8)

MDLYYMPISPPCWSILLLGRQLDLTFNLKEIDFKAEEHKKPEFLKINPAHTVPTLAVGDGYALSESRAILVYLVESLKTEGQENSLYPRDAKTRGLIHNRLDFDLGTLYQRIIAYCSPQWKSGSMGTEENRTKVQDAFELLEVFLSKTKYVAADQLTIADISLFVSVSLLDLCYFDRSGYGKVAAWHDVLKKELVGYEDVIAKGFPEWRKH

>Aedes aegypti(Q17MB1)

MELYYSHASAPCRAVQMTALALGVQLNLKEIHLMNGKDHQRPDYGRITPQHSIPTLKDKDLILWESRAIQMYLVQQYGKDDSLYPKDPSKQAKVNERLFFDACILYHRFTEYYHEQVYGGLEGDDKKLAALEDAVKMLDLFLEGQPYVTGEAMTIVDLSMLATVATMNCLGFELKPYHNVFEWYKHMKDVAPGSKFNETGAKEFAAFK

>Aedes aegypti(Q16SH6)

MPLDLYCHIVAPFCRSVILLADALEVELNFIEVNVLKKEQFKPEFIAMNPQHCIPTLVDGDVVVWESNAILIYLAEKYGKVSKRFYPTDIAERAKVNRILFFQLGTLHRALSTYYYPILAGLGEGKPEDFRKIQDAVGVMDKLLDGNKWLAGEDLSIADFSVVISVASLEGVIKFDLTVYKNVYRWYQQCKKEFRKFEELTQEANDKSQEMIAALRQYKLEEINSAKEPCCSAPPGAKTPPKPPCPDSS

>Aedes aegypti(Q17MA8)

MTPVLYFLPASPPCRAVMLLAKMIGVDLEYKTLNVMEGEQLRPEFVELNPQHTIPTLDDHGLVLWESRVILSYLVSAYGKDESLYPKDFRSRAMVDQRLHFDLGTLYQRVVDYYFPTIMVGAHLDQTKKAKLAEALGWFDAMLKQYQWAAANHFTIADVTLCVTVSQIEAFEFDLHPYPKVRAWLAKCKEELEPHGYKDINQTGAEALAGLFRAKLKQ

>Plutella xylostella(219AA)

MAIDLYLTPGSAPCRLVLLTAAALNIQLNLNHVDLRAGEQFSPDFLKLNPQHTIPTIVDQGFALWESRAISRYLVNKYGHGSTLYPEDPQSRALVDQRLDFDLGTLYPKFADYFYPQVFGSAPADPEKLKKLHEVLGFLDIFLGDEKYAAGSDLTIADLSLVATVSTIDAAGISLDDFPNIHRWFELVKSTAPDYENANGKGIKAFKELVAQLNAKTEL

>Plutella xylostella(223AA)

MPAIELYEMQGSAPCRAVRLTARALGKPLTVHHLDLMAGEHLKPEFVKINPQHTIPTIVDDGFALWESRTIMRYLVNKYGKGSSLYPEEPKARALVDQRLDFDLGTLYAKYAVYFYPQVFGTAPENAEDLKKLNEALAHLNTFLGESKYAAGSNLTIADFSLVATISTIDVSDIVDLKQYPNIVKWYEHLQSSVEGYEEENLAGLENFRSFIKEFKAKKAAAK

>Drosophila melanogaster(Q9VG98)

MDFYYMPGGGGCRTVIMVAKALGLELNKKLLNTMEGEQLKPEFVKLNPQHTIPTLVDNGFSIWESRAIAVYLVEKYGKDDYLLPNDPKKRAVINQRLYFDMGTLYESFAKYYYPLFRTGKPGSDEDLKRIETAFGFLDTFLEGQEYVAGDQLTVADIAILSTVSTFEVSEFDFSKYSNVSRWYDNAKKVTPGWDENWEGLMAMKALFDARKLAAK

>Drosophila melanogaster(Q9VG97)

MVGKALGLEFNKKIINTLKGEQMNPDFIKINPQHSIPTLVDNGFTIWESRAILVYLVEKYGKDDALYPKDIQKQAVINQRLYFDMALMYPTLANYYYKAFTTGQFGSEEDYKKVQETFDFLNTFLEGQDYVAGDQYTVADIAILANVSNFDVVGFDISKYPNVARWYDHVKKITPGWEENWAGALDVKKRIEEKQNAAK

>Drosophila melanogaster(Q9VG96)

MDFYYSPRSSGSRTIIMVAKALGLELNKKQLRITEGEHLKPEFLKLNPQHTIPTLVDNGFAIWESRAIAVYLVEKYGKDDSLFPNDPQKRALINQRLYFDMGTLHDSFMKYYYPFIRTGQLGNAENYKKVEAAFEFLDIFLEGQDYVAGSQLTVADIAILSSVSTFEVVEFDISKYPNVARWYANAKKITPGWDENWKGLLQMKTMYEAQKASLK

>Drosophila melanogaster(Q9VG95)

MDFYYSPRGSGCRTVIMVAKALGVKLNMKLLNTLEKDQLKPEFVKLNPQHTIPTLVDNGFSIWESRAIAVYLVEKYGKDDTLFPKDPKKQALVNQRLYFDMGTLYDSFAKYYYPLFHTGKPGSDEDFKKIESSFEYLNIFLEGQNYVAGDHLTVADIAILSTVSTFEIFDFDLNKYPNVARWYANAKKVTPGWEENWKGAVELKGVFDARQAAAKQ

>Drosophila melanogaster(Q9VG94)

MDLYNMSGSPSTRAVMMTAKAVGVEFNSIQVNTFVGEQLEPWFVKINPQHTIPTLVDNLFVIWETRAIVVYLVEQYGKDDSLYPKDPQKQALINQRLYFDMGTLYDGIAKYFFPLLRTGKPGTQENLEKLNAAFDLLNNFLDGQDYVAGNQLSVADIVILATVSTTEMVDFDLKKFPNVDRWYKNAQKVTPGWDENLARIQSAKKFLAENLIEKL

>Drosophila melanogaster(Q9VG92)

MDFYYHPCSAPCRSVIMTAKALGVDLNMKLLKVMDGEQLKPEFVKLNPQHCIPTLVDDGFSIWESRAILIYLVEKYGADDSLYPSDPQKKAVVNQRLYFDMGTLFQSFVEAIYPQIRNNHPADPEAMQKVDSAFGHLDTFLEDQEYVAGDCLTIADIALLASVSTFEVVDFDIAQYPNVARWYENAKEVTPGWEENWDGVQLIKKLVQERNE

>Drosophila melanogaster(Q9VGA0)

MLDFYYMLYSAPCRSILMTARALGLELNKKQVDLDAGEHLKPEFVKINPQHTIPTLVDDGFAIWESRAILIYLAEKYDKDGSLYPKDPQQRAVINQRLFFDLSTLYQSYVYYYYPQLFEDVKKPADPDNLKKIDDAFAMFNTLLKGQQYAALNKLTLADFALLATVSTFEISEYDFGKYPEVVRWYDNAKKVIPGWEENWEGCEYYKKLYLGAILNKQ

>Anopheles gambiae(Q8MUS3)

MDYYCNFVSPPSQSVILVAKKLGIKLNLRKINIYDPVAMDTLSKLNPHHILPMLVDNGTVVFEPCAIVLYLVEMYAKNDALYPKDALVRCVVNQRLFFDVSTLFKQIYENVHVQMRNSQPSEKQVQRLQKAVDVLESFLYERSYTAADQLTVADICLLVTVNALTLWLGYELAPYPRIRDWLGRVVAEIPGCAEFQREVEDATRAYVVNRKI

>Anopheles gambiae(Q8MUS4)

MELYSDIVSPSCQNVLLVAKKLGIALNIKKTNIMDATDVAELTKVNPQHLIPTFVEDDGHVIWESYAIAIYLVEKYGQDDALYPKDPKVRSIVNQRLFFDIGTLYKNILANVDVLIEKQQPSAELRGKLEQALDLTEKFVTECRFVAADHLTLADIFMLGSITALEWFRYDLERYPGIRGWVERVTAQFPDYSNFHKEIREATKQYVATHCPHLEY

>Anopheles gambiae(O76483)

MTPVLYYLPPSPPCRSVLLLAKMIGVELELKALNVMEGEQLKPDFVELNPQHCIPTLDDHGLVLWESRVILAYLVSAYGKDENLYPKDFRSRAIVDQRLHFDLGTLYQRVVDYYFPTIQLGAHLDQTKKAKLAEALGWFEAMLKQYQWSAANHFTIADIALCVTVSQIEAFQFDLHPYPRVRAWLQKCKDELQGHGYKEINETGAETLAGLFRSKLKQ

>Anopheles gambiae(9GPL7)

MDFYYHPASPYCRSVMLVAKALKLSLNLQFVDLMKDEQLRPTFTVLNPFHCVPTLVDNDLTMWESRAILVYLVDKYGRTNSRLYPKDAKTRAIINQRLFFDHGTLGTRLEDYYYPLYFEGATPGGEKLEKLEEALAVLNGYLINNPYAAGPNITLADYSLVSTVTSLEVVQHDLSKYPAISAWYEGCKATMADFQEINESGMQQYRLTSSLVPHLQLLHMPFAE

>Anopheles gambiae(Q86D84)

MDLYYNILSPPSRAILLLGEALQLKFNLISLDVHRKDYVNPAFKKINPQHTVPTLVVDGVAICEPGAILIYLAEQYAPAGTTYYPPDPLRRAIVNQRLLFECGTLYKCIFVYYSPVVLERATPVETDRQKLIEAVAVLDGILQHSAFVAGDCLTVADYSLVCTVSMLVVLKFELAPYAAVRRWYERCKEVIAGYTDLTQRAVTMFQKWMEQENSKG

>Bombyx mori(Q60GK5)

MTIDLYYVPGSAPCRAVLLTAKALNLNLNLKLVDLHHGEQLKPEYLKLNPQHTVPTLVDDGLSIWESRAIITYLVNKYAKGSSLYPEDPKARALVDQRLYFDIGTLYQRFSDYFYPQVFAGAPADKAKNEKVQEALQLLDKFLEGQKYVAGPNLTVADLSLIASVSSLEASDIDFKKYANVKRWYETVKSTAPGYQEANEKGLEAFKGLVNSMLKK

>Bombyx mori(Q2I0J5)

MAIDLYFTAGSAPCRVVLLVAAALDLQLNLKPLNLWEREQLQADFLKLNPQHTVPTIVDEGFPLWESRAISRYLVNKYGGDSSSLYPKDLMARALVDQRLDFDIGTLYPRFAQYFYPQVFGGAKPDAAALKKLEEALVFLNAFLEGQKYVTGDVLTIADLSLVATISTIDAAEISLKSYPNVEKWFELMKTTAPDYQNANQKGIDEFKKLIAQMKAKTEL

>Bombyx mori(H9JAJ3)

MLTASVLGVELELIAVNILDNEHKTPEYLKMNPQHTIPTMDDNGFILWESRAIQAYLVNAYGKNDALYPKNPRLRAIIDQRLNFDLGTLSRRWIDLYVPMLIKGEPFDDEKGEKLNEALELLNIFLEGHAFVAGENMSIADLSIVVTISNLDAVEYDLSSYDNVRKWFERMKIALKPYDYEDIDQTGAEILASFINKDDE

**Epsilon Class GSTs**

>Aedes aegypti(Q170C6)

MSPITLYTTRRTPAGRAVEITAKLIGLELDVKFIDLSKKEHLTEEFLKLNPQHTVPTIVDNGVALYDSHAIIVYLVQKYGKDDALYPKDLVTQARVNALLHFESGILFARLRGTLEPIFYHGFPEVPQEKLTAIHGAYDLLEATLKSDYLVGDSLTLADVSCSTSLSTLNALFPIDAEKCPKLVAYLQRLEANMPNYKELNSDRAAEAVAFFKVKLEENKKK

>Danaus plexippus(A0A212F3G0)

MMVAEILGVQYSNCEINPVLREQDTPEMTAKNPLRTIPYIEDDGFCLGDSHAIILYLFDKYAKPEHDHLYPSNVKIRAKINQILFFDCGVLFARLRSVMAPTYMGRLSELSQSMKRNIEDAYRIIEAYLSNTLYIADNNVTLADYSVLATMSSLHGLHPIDSNKYPKLLNWYKRMSSLPVCKTINDPGAELHVTGLKKLMEQRKLSKL

>Danaus plexippus(A0A212FES8)

MSKGRLNEVTKLPRLLLYKRNASPPSSAVMILGDMLGLNFDYREPDLIKLEHRSPEFKKINPMATILVLQDGDVTICESHAIMKYLVNKYGGERCERLYPADLSVRANIDQLMFYDAGVLFVRLKVVALPTMLQGLTGPTKEQVADIDEGYTVLEAYLNKHSYIATDHLTIADLSVGTTTTALQSVHKLDKNRFPLSAEWLERLKGEKSFKKFNEPSVKELSTILNVFWKKNKERIR

>Aedes aegypti(Q5PY78)

MGKVQLYTAKLSPPGRAVELTAKAIGLDLDVHPINLIAGDHLKPEFVKMNPQHTIPLIVDEDGTIVYDSHAIIIYLVSKYAKDDSLYPKDIATRAKINAALHFDSGVLFARLRFYLEPILYYGSPDTPQDKIDYACKAYQLLNDTLVDEYIVGNRMTLADLSCIASIASYHAIFPIDAAKYPKLAAWVQRLEKLPYYKGTNQEGAEELAAVYRDRLAQNRAGKK

>Aedes aegypti(Q170C9)

MTKPIVYTLYLSPPSRAVDLCAVALGIELERKVMNLLEREHLDPKFLKMNPQHTIPVLDDGGIIVRDSHAIMIYLVSKYGKDDSLYPKDLAEQAKVNAALYFDCGVLFARLRFITEQILMGGSEIPAEKAAYVESAYQLLEDALTDDFIAGNSLTIADLSCGSTVSTAMGLIPMDRDKYPKIYAWLNRLKALPYFEELNDQGAVELPAIMKNLMETNARKA

>Aedes aegypti(Q170C7)

MATNSPRIKLYTNPISPPGRSVELTAKAIDLDIEVIAIDLLGNDTLKPDYLLKNPQHTIPMIDDGGKFIWDSQAINVYLTTVYSRNEDLYPNDPFVRAKVNAGLHFNSGVLFSRLKLLISPVIRGFKQDLDPEKVEYFNIGLQLLEDTLHADYYIGNRMTLADLSCVSSVSSFDAVLPLDNSRFPKTVDWLRRMEQLPYYGEANGEGAKKLAKVVQSFLK

>Aedes aegypti(Q170C8)

MSSKIVLHTTRRTPGGRAVQILSHILGLDLDLKFVDLSKKEQMSEEFLKLNPFHTIPTIDDDGVPVYDSHAILVYLVSKYAKDRDLFPEDPVIQARINAWFHFDSGVLFPRLRGAVEPVFYFGLKKIPRDRMEAIEAAYDLFEGALKGDFLVGDSLTLADISVTTCLVSLNGVCPMEESKYPKSCAFLKRMEQSMPCYKEFNAEVLEETKVFLKQKLDENNKK

>Plutella xylostella(220AA)

MGLTVYKIDWSPPARAVIMTLEALNITDAELVDVSLLDGKHMSEEYLKMNPQHTVPVIKDGDFVLWDSHAICAYLVDKYGKDDSLYPKDLQKRAVVDQRLHFDTGILFPSVRGAAEPVLFDWEPTFNPEKLKVIQSGYDFLEKFLDHSYLAGDHLTIADICAGATVSSMNVIVPIAANRYPKISAWLDRLNSIEYFSRINGNGIKIITALFESKLNKSKK

>Plutella xylostella(216AA)

MVLTLYKLDASPPARAVMMTLEALGIRDVEMVDVNLFEGAQFTEEYVKMNPQHTIPALKDDDFAIWDSHAICPYLVSKYGQDDSLYPQDPQRRAVIDQRLHFDSGILFPSLRATVAPVLFLGERSFKPEGLQAIKAGYDFLEKFLDKPYCAGDQLSIADICTSATVSSMSAALPIDADTYPNITAWLDRLSKEEFYTKVNLPGLQQFSGALKSKLL

>Drosophila melanogaster(A1ZB68)

MGKLTLYGIDGSPPVRSVLLTLRALNLDFDYKIVNLMEKEHLKPEFLKINPLHTVPALDDNGFYLADSHAINSYLVSKYGRNDSLYPKDLKKRAIVDQRLHYDSSVVTSTGRAITFPLFWENKTEIPQARIDALEGVYKSLNLFLENGNYLAGDNLTIADFHVIAGLTGFFVFLPVDATKYPELAAWIKRIKELPYYEEANGSRAAQIIEFIKSKKFTIV

>Drosophila melanogaster(A1ZB69)

MGKISLYGLDASPPTRACLLTLKALDLPFEFVFVNLFEKENFSEDFSKKNPQHTVPLLQDDDACIWDSHAIMAYLVEKYAPSDELYPKDLLQRAKVDQLMHFESGVIFESALRRLTRPVLFFGEPTLPRNQVDHILQVYDFVETFLDDHDFVAGDQLTIADFSIVSTITSIGVFLELDPAKYPKIAAWLERLKELPYYEEANGKGAAQFVELLRSKNFTIVS

>Drosophila melanogaster(A1ZB70)

MVKLTLYGVNPSPPVRAVKLTLAALQLPYEFVNVNISGQEQLSEEYLKKNPEHTVPTLEDDGNYIWDSHAIIAYLVSKYADSDALYPRDLLQRAVVDQRLHFETGVVFANGIKAITKPLFFNGLNRIPKERYDAIVEIYDFVETFLAGHDYIAGDQLTIADFSLISSITSLVAFVEIDRLKYPRIIEWVRRLEKLPYYEEANAKGARELETILKSTNFTFAT

>Drosophila melanogaster(A1ZB71)

MVKLTLYGLDPSPPVRAVKLTLAALNLTYEYVNVDIVARAQLSPEYLEKNPQHTVPTLEDDGHYIWDSHAIIAYLVSKYADSDALYPKDPLKRAVVDQRLHFESGVVFANGIRSISKSVLFQGQTKVPKERYDAIIEIYDFVETFLKGQDYIAGNQLTIADFSLVSSVASLEAFVALDTTKYPRIGAWIKKLEQLPYYEEANGKGVRQLVAIFKKTNFTFEA

>Anopheles gambiae(Q8WQJ9)

MAPIVLYSTRRTPAGRAVELTAKMIGIELDVQYIDLAKKENMTEEYLKMNPMHTVPTVNDNGVPLYDSHAIINYLVQKYAKDDTLYPAKDLVKQANINALLHFESGVLFARLRWILEPVFYWGQTEVPQEKIDSVHKAYDLLEATLKTSGTDYLVGGTITLADISVSTSLCTLNALFPADASKYPLVLAYLKRLEQTMPHYQEINTDRANDALQLYNQKLGKV

>Anopheles gambiae(Q8WQJ8)

MPNIKLYTAKLSPPGRSVELTAKALGLELDIVPINLLAQEHLTEAFRKLNPQHTIPLIDDNGTIVWDSHAINVYLVSKYGKPEGDSLYPSDVVQRAKVNAALHFDSGVLFARFRFYLEPILYYGATETPQEKIDNLYRAYELLNDTLVDEYIVGNEMTLADLSCIASIASMHAIFPIDAGKYPRLAGWVKRLAKLPYYEATNRAGAEELAQLYRAKLEQNRTNAK

>Anopheles gambiae(Q8WQJ7)

MATNPIIKLYTAKLSPPGRAVELTAKLLGLSLDIVPINLLAGDHRTDEFLRLNPQHTIPVIDDGGVIVRDSHAIIIYLVQKYGKDGQTLYPEDPIARAKVNAGLHFDSGVLFSRLRFYFEPILYEGSAEVPQDKIDYMKKGYELLNDALVEDYIAGSSLTLADVSCIATIATMEEFFPMDRSRYPALVAWIERLSRTLPEYDQLNQEGAVEFAEICESLRLKNGASVAAK

>Anopheles gambiae(Q8MUQ7)

MEPSRLVLYTNRKSPPCRAVKLTARALGIELVEKEMTLLRGDKLMEEFLKVNPQQTIPVLDDGGIVITASHAITIYLVCKYGRDDGLYPSELVRRARVHTALHLEAGVIFSRLSFLFEPVIYSGKSYFHSDRIEHIRKAYRLLEDSLVDQYMVGESLTIADFSCISSIATLVGVVPLDESKFPKSTAWMRRMQELPYYEEANGTGALELAEFVLGKKEANASQFL

>Anopheles gambiae(Q8WQJ5)

MILYYDEVSPPVRGVLLAIAALGVKDRIKLEYIDLFKGGHLSSDYLKINPLHTVPVLRHGELTLTDSHAILVYLCDTFAPPGHTLALPDALTRAKVFNMLCFNNGCLFQRDAEVMRKIFSGAITDPTQHLKPIEAAIDALEQFLQRSRYTAHDQLSVADFAIVATLSTVAIFVPLPADRWPRVCEWFAVMEALPYYNDQNRVGLDMLRKHLAGKIKL

>Bombyx mori(B0LB15)

MSLMLYKLNASPPARTAMMVCELFKVPVKMVDVNLSKGEHFSPEYLKRNPLHTVPTLEDGDLIITDSHAIAMYLADKYGKDDSLYPKDLKSRAIVNQRLFFDSTVLFSRMRSVTFPVIIEGCKTVTEKQINDIIEAYGYVETYLSNTKFIATNNLTIADISAYAVVSSLLFIVPLDGAKFPKTQTWLNEMEKKPFAQKYNVNGVAELGALLKEKLGS

>Bombyx mori(B0LB16)

MVFILYKKDTSPPCRSVQMVLHELGIYDVELIEVNLPERDHLKEEFLRMNPQHTVPTLIDGDFIIWDSHAIVTYLVNRYAKNDTLYPKEPKQRAIVDQRLHFDTGVLFAILRATAEPVLYNNEKSFKQENLEKMEAAYEFVEKFLTSDWLAGDQVTLADICCVSTISSMNVIVPIDKKKYPKIISWLQRCSEQEFYKKANEPGLKKFIEMFKNKIGN

>Bombyx mori(B0LKP4)

MVLTLYKLDASPPVRSVYMVIEALKIRDVEYVDVNLLEGSHLKEEFLKMNPQHTIPLLKDDDFLIWDSHAISGYLISVYGADDSLYPNEPKKRALIDQRLHFDSGILFPALRGVAVIIFFNLLCLGQDELIIFRGEKEIRPENLAKIKSAYDFTEKILSSDWIAGDEFSLADICCVTSISTLNEMVPIDGSLYPKLASWLDRSSQLPIYKKANEPGLLQFREIFKNKTS

>Bombyx mori(B0LKP5)

MTPILYKTDASPPARAVMMIVDILGLKVDEQELNPILRQQDTPEFKKKNPMRTIPILEEGDFYLADSHAIMLYLIDKYGKPEHAHLYPSEKRKRATINQRLFFDCGVLFPRLRAVMAPTYAGKLAELNRNMIKNIEDAYSIMESYLTENLYLADEVVTVADISAITTISSLNGLYPVDEKSKWINRMNDKEYCRKINTPGSELHVAGLIALMDNTKHNQQSKL

**Kappa Class GSTs**

>Macaca fascicularis(A0A023JCQ9)

MGPLLRTVELFYDVLSPYSWLGFEVLCRYQNIWNINLQLRPSLIGGIMKDSGNKPPGLLPRKGQYMANDIKLLRHHFQIPIQFPKDFFSVIIEKGSLSAMRFLTAVSLEHPEMLEKVSRELWMRVWSRDEDITQPQSILAAAEKAGMSAEQAQGLLEKISTPKVKNQLKETTEAACRYGAFGLPITVAHVDGQTHMIFGSDRMELLAFLLGEKWMGPVPPAVNARL

>Rattus norvegicus(P24473)

MGPAPRVLELFYDVLSPYSWLGFEVLCRYQHLWNIKLKLRPALLAGIMKDSGNQPPAMVPHKGQYILKEIPLLKQLFQVPMSVPKDFFGEHVKKGTVNAMRFLTAVSMEQPEMLEKVSRELWMRIWSRDEDITESQNILSAAEKAGMATAQAQHLLNKISTELVKSKLRETTGAACKYGAFGLPTTVAHVDGKTYMLFGSDRMELLAYLLGEKWMGPVPPTLNARL

>Homo sapiens(Q9Y2Q3)

GPLPRTVELFYDVLSPYSWLGFEILCRYQNIWNINLQLRPSLITGIMKDSGNKPPGLLPRKGLYMANDLKLLRHHLQIPIHFPKDFLSVMLEKGSLSAMRFLTAVNLEHPEMLEKASRELWMRVWSRNEDITEPQSILAAAEKAGMSAEQAQGLLEKIATPKVKNQLKETTEAACRYGAFGLPITVAHVDGQTHMLFGSDRMELLAHLLGEKWMGPIPPAV

>Mus musculus(Q9DCM2)

GPAPRILELFYDVLSPYSWLGFEVLCRYQHLWNIKLQLRPTLIAGIMKDSGNQPPAMVPRKGQYIFKEIPLLKQFFQVPLNIPKDFFGETVKKGSINAMRFLTTVSMEQPEMLEKVSREIWMRVWSRDEDITEYQSILAAAVKAGMSTAQAQHFLEKISTQQVKNKLIENTDAACKYGAFGLPTTVAHVDGKTYMLFGSDRLELLAYLLGEKWMGPVPPTA

>Xenopus tropicalis(Q5M8U8)

MSNRRVLECFYDVVSPYSWLGFEVVCRYKNIWNVDALLRPGFLGGIMQASGNSPPAMVPKKGVYMAQDIARLSDFFQVPLRQPSNFFQSVIKKGSLQAMRFVTAVEMQHPEFLEPVSRELWRRIWSEDKDITEPESILEAAKKAGMSADQAKKLIESTALPEVKNKLKQNTDEALKYGAFGMPIIVAHVDGKPHMYFGSDRFELLAHQLGEKWMGPVPQKPRL

**Lambda Class GSTs**

>Sorghum bicolor(A0A1B6P694)

GTTRLYISYICPYVQRVWIARNFKGLQEKIQLVAIDLQDKPAWFLEKVYPPGKVPVLEHNGNIIAESLDLLSYLDANFEGPKLFPGDQDPAKQAFADELIANSDSIIIALFRAGRAYAEGQGDDDISKLLAPALDKVEESLGRFSDGPFLLGQSMSAVDMVYAPFIERFKDFFAAVKHYDMTQERPKLKEWIEELNKIDAYTATWGDRRLQLAALMNKFGIQSPV

>Glycine hispida(I1L8Q0)

GTTRLYISYSCPYAQRVWIARNFKGLKDKINLVPINLQDRPAWYKEKVYPENKVPSLEHNGKVLGESLDLIKYVDENFEGTPLFPRDPAKKEFGEQLISHVDTFSRDLFVSLKGDAVQQASPAFEYLENALGKFDDGPFLLGQFSLVDIAYIPFAERFQIVFAEVFKHDITEGRPKLATWFEELNKLNAYTETRVDPQEIVDLFKKRF

>Pinus tabuliformis(L7S317)

GTTRLYISVACPYAQRVWSARNIKGLSQIQLVPIDLQDRPAWYKEKVYPPNKVPAIEHNGKVTGESLDLLEYLENNFEGPKLFPTDPAKKEAANELLKYTDTFTKNSFIALTKPDSETAQEAGPALDYLENALGKFSDGPFLLGQFSVVDIAYGPFVERFHVAFPALKNYDITAGRPKLSKWIQELHKIEGYAKTVSDPESIVETYKRI

>Arabidopsis thaliana(Q6NLB0)

TTRLYISYTCPFAQRVWITRNLKGLQDEIKLVPIDLPNRPAWLKEKVNPANKVPALEHNGKITGESLDLIKYVDSNFDGPSLYPEDSAKREFGEELLKYVDETFVKTVFGSFKGDPVKETASAFDHVENALKKFDDGPFFLGELSLVDIAYIPFIERFQVFLDEVFKYEIIIGRPNLAAWIEQMNKMVAYTQTKTDSEYVVNY>

>Arabidopsis thaliana(Q9M2W2)

MSVGLKVSAFLHPTLALSSRDVSLSSSSSSLYLDRKILRPGSGRRWCKSRRTEPILAVVESSRVPELDSSSEPVQVFDGSTRLYISYTCPFAQRAWIARNYKGLQNKIELVPIDLKNRPAWYKEKVYSANKVPALEHNNRVLGESLDLIKYIDTNFEGPSLTPDGLEKQVVADELLSYTDSFSKAVRSTLNGTDTNAADVAFDYIEQALSKFNEGPFFLGQFSLVDVAYAPFIERFRLILSDVMNVDITSGRPNLALWIQEMNKIEAYTETRQDPQELVERYKRRVQAEARL

>Arabidopsis thaliana(Q9LZ06)

MAPSFIFVEDRPAPLDATSDPPSLFDGTTRLYTSYVCPFAQRVWITRNFKGLQEKIKLVPLDLGNRPAWYKEKVYPENKVPALEHNGKIIGESLDLIKYLDNTFEGPSLYPEDHAKREFGDELLKYTDTFVKTMYVSLKGDPSKETAPVLDYLENALYKFDDGPFFLGQLSLVDIAYIPFIERFQTVLNELFKCDITAERPKLSAWIEEINKSDGYAQTKMDPKEIVEVFKKKFM

>Glycine max(I1LYZ1)

MLMATLGLQAVRPPPLTSTSDPPPLFDGTTRLYISYSCPYAQRVWITRNYKGLQDKIKLVPIDLQDRPAWYKEKVYPENKVPSLEHNGKVLGESLDLIKYVDVNFEGTPLVPSDPAKKEFGEHLISHVDTFNKDLNSSLKGDPVQQASPSFEYLENALGKFDDGPFLLGQFSLVDIAYIPFIERYQIVFAELFKQDIAEGRPKLAAWIEEVNKIDAYTQTKNDPQEIADKYKKRLLSQQ

>Pinus tabuliformis(L7S6H2)

MAALYAVPPPLTSKSEPPPLFDGTIRLYINVLCPYAQRAWSARNIKGLSEIQIVSIDLQDRPAWYKEKVYPPNKVPALEHNGKVTGESIALLEYLENNFEGPKLFPTDPAKTEAATELLKHTDTFTQNLFGALTKPEPKAAQEAGPALDYLENALGKFADGPFFLGQLSVVDIAYGPFFERFQVVFPALKNSAGRPKLLKWIQELHKIEGYAKTVADPEIIVEIFKTRQANSAK

>Glycine max(I1NA39)

MATAGVQEVRVPPLTSTSEPPSLFDGTTRLYISYICPYAQRVWITRNYKGLQDKIKLVPIDLQNRPAWYKEKVYPENKVPSLEHNGKVLGESLDLVKYIDDNFEGPSLVPSDPAKKEFGEELISHVDTFTKELYSALKGDPIHQAGPAFDYLENALGKFGDGPFFLGQFSWVDIAYVPFVERFQLVFADVFKHDITEGRPKLATWIEEVNKISAYTQTRADPKEIVDLFKKRFLAQQ

>Pinus tabuliformis(L7S0Z7)

MAALYSIPPALTSKSEPPPLFDGTTRLYICVKCPYAQRAWSARNIKGLSQIQIVPIDLQDRPVWYKEKVYPPNKVPALEHNGKVTGESMDLLEYLDNNFEGPKLFPTDPAEKEAANELLKHTDAFTKTVFVALTKPDSEAAQEAGPALDYLENALGKFSDGPFLLGQFSVADIAYGPFVERFQVAFPALKNYDITAGRPKLLKWIQELHKIEGYAKTTVADPEIILETYKRILAIFTKRL

>Capsella rubella(A0A140EH50)

MSVGVNVSVCSYSSLALSTKDFSSISSSSLSLDRNILRPVSGRLRCKSSGKRRIEPILAVKESSRVPELDSSSEPVQVFDGSTRLYISYSCPFAQRAWIARNYKGLQNKIELVPIDLKNRPAWYKEKVYAANKVPALEHNNRVIGESLDLIKYIDTNFEGPSLTPDGLEKQAAADELLSYTDSFSKAVRSTLNGTDSNAADATFDYIEQALSKFNEGPFFLGQFTLVDVAYAPFIERFQLILRDVTNVDITSGRPNLTLWIQEMYKIEAFTETRQDPKELVERYKKRVQAEARL

>Populus trichocarpa(D2WL72)

MELPRLYTCYTCPFAHRVWITRNFKGLQDEIKLVPLILQNRPAWYSEKVYPPNKVPSLEHNGKITGESLDLIKYLESNFQGPSLLPEDPAKKEFAEELFSYTDTFNRTVFTSFKGDPAKEAGPAFDHLENALHKFGDGPFFLGQEFSLVDIAYIPFVERFCIFLSEVFKYDITAGRPKLAAWIEELNKIEAYKQTKTDPKEMVEVYKKRFMA

>Physcomitrella patens subsp. patens(K9Y419)

MATLVTSYLHACNATFATTLPRHSRLIQAPSVQFSQVCGKNLGQSFSSPSARILRRNYDFRRELVSVTRSMASSFENREVLDSKSASPAIFDGTTRLYFSSRCPYAQRVWVAVKYKGLDEIECVEISLSDKPTWYKEKVYPVGKVPALEHNGTVTGESMDLLTYLDDHFGGPKLAPTEESKKQAAAELLQYADTFNKLGFTGLSMKSSTPDEIAAAVAPAFDFLENALAKFSSEGPLFLGNFGLVDIVYAPFIERFEIAFGGIRNYDIRAGRPRLAKWIEAMDNVEAYSSTKVPRATLLELYKKMLENDYFIRVGVAANQNNSSGSSVAVN

>Larix kaempferi(V5L6L6)

MAALDLIPPVLNSKSEPPPLFDGTTRLYISVTCPYAQRVWSARNIKGLSEIQIVPIDLQDRPAWYKEKVYPPNKVPSLEHNGKIIGESLDLLEYLENNFEGPKLFPTDPAKKEAANELLKYTDTFTKSLFIGLTKPEPEVAQEAGPALDYLENALGKFADGPFFLGEFSVVDIAYGPFVERFQIVYPALKNNDITSDRPKLLKWIQELHKIDAYAKTKVDPETIVETYKRILAKKGDPNH

>Larix kaempferi(V5L7R3)

MAAIDLIPPVLNSKSEPPPLFDGTTRLYISVICPYAQRVWSARNIKGLSQIQIVPIDLQDRPAWYKEKVYPPNKVPSLEHNGKVIGESLDLLEYLENNFEGPKLFPTDSEKIEAANVLLKYTDTFTKDLFIGLTKPEPEAAQEAGPALDYLENALGKFADGPFLLGEFSLVDIAYGPFVERFQIVYPVLKNYDITADRPKLLKWIQELHKIDAYAKTVTDSETTVEIYKRLLANYAK

>Populus trichocarpa(D2X9U2)

MGSLRLYTCYTCPFAQRVWITRNFKGLQDEIKLVPLILQNRPAWYPEKVYPPNKVPSLEHNGKITGESLDLIKYLESNFEGPSLLPQDPAKKEFAEELFSYTDKFNGTVYTAFKGDLAKSGPAFDYLENALHKFDDGPFFLGKCCQVDIAYIPFVERLNIFLLEVFKYDIAAGRQKLAAWIEEVNKIEAYKQTKTDPKELVEFYKKRFVSHS

>Populus trichocarpa(D2WL71)

MEQQRLYISYTCPYAQRVWITRNCKGLQDKIKLVPIDLQDRPAWYKEKVYPPNKVPSLEHNNEVKGESLDLIKYIDSHFDGPSLFPDDPAKKEFAEDLFSYTGSFSKANNSTFKGEADEAGAAFDYIETALSKFDDGPFFLGQFSLVDIAYAPFIERFQPALLEFKKYDITAGRPKLAAWIEEMNKIEAYNQTRREPKQHVGTYKKRFEAHL

**MAPEG GSTs**

>Homo sapiens(O14880)

MAVLSKEYGFVLLTGAASFIMVAHLAINVSKARKKYKVEYPIMYSTDPENGHIFNCIQRAHQNTLEVYPPFLFFLAVGGVYHPRIASGLGLAWIVGRVLYAYGYYTGEPSKRSRGALGSIALLGLVGTTVCSAFQHLGWVKSGLGSGPKCCH

>Homo sapiens(Q99735)

MAGNSILLAAVSILSACQQSYFALQVGKARLKYKVTPPAVTGSPEFERVFRAQQNCVEFYPIFIITLWMAGWYFNQVFATCLGLVYIYGRHLYFWGYSEAAKKRITGFRLSLGILALLTLLGALGIANSFLDEYLDLNIAKKLRRQF

>Mus musculus(Q9CPU4)

MAVLSKEYGFVLLTGAASFVMVLHLAINVGKARKKYKVEYPVMYSTDPENGHMFNCIQRAHQNTLEVYPPFLFFLTVGGVYHPRIASGLGLAWIIGRVLYAYGYYTGDPSKRYRGAVGSLALFALMGTTVCSAFQHLGWIRPGLGYGSRSCHH

>Bos taurus(Q3T100)

MAVLSKEYGFVILTGAASFLMVTHLAINVSKARKKYKVEYPTMYSTDPENGHIFNCIQRAHQNTLEVYPPFLFFLAVGGVYHPRIVSGLGLAWIVGRVLYAYGYYTGEPRKRQRGALSFIALIGLMGTTVCSAFQHLGWVRTGLNSGCKSCH

>Bos taurus(Q64L89)

MANLSQLMENEVFMAFASYTTIVLSKMNFMSTATAFYRLTKKVFANPEDCAGFGKGENAKKYLRTDDRVERVRRAHLNDLENIVPFLGIGLLYSLSGPDLSTAILHFRLFVRARIYHTIAYLTPLPQPNRALAFFIGYGVTLSMAYRLLKSKLYL

>Bos taurus(Q2KJG4)

MAGNSILLAALSVLSACQQSYFAMQVGKARSKYKVTPPSVSGSPDFERIFRAQQNCVEFYPIFIITLWMAGWYFNQVFATCLGLVYIYSRHQYFWGYAEAAKKRVTGFRLSLGVLALLTVLGAVGILNSFLDEYLDIDIAKKLRHF

>Rattus norvegicus(A0A0G2JU12)

GYFALQVGRVRLKYKIAPPAVTGSLEFERIFRAQQNSLEFYSVFIISLWMAGWYFNQVFATCLGLLYIYARHKYFWGYAEAAEKRIIGFRLSLGILALLTVLAVLGVASRFLDEYLDFHVAKKLKRPF

>Drosophila melanogaster(Q8SY19)

MASPVELLSLSNPVFKSFTFWVGVLVIKMLLMSLLTAIQRFKTKTFANPEDLMSPKLKVKFDDPNVERVRRAHRNDLENILPFFAIGLLYVLTDPAAFLAINLFRAVGIARIVHTLVYAVVVVPQPSRALAFFVALGATVYMALQVIASAAF

>Macaca mulatta(F6RJ20)

MAGNSILLAAVSILSACQQSYFALQVGKARLKYKVTPPAVTGSPEFERVFRAQQNCVEFYPIFIITLWMAGWYFNQVFATCLGLMYIYGRHLYFWGYSEAAKKRITGFRLSLGILALLTLLGALGIANSFLDEYLDLNIAKKLRRQF

>Danio rerio(Q5XJJ0)

MAEVVHMIDSEVFLAFSTYATIVILKMMLMSLMTSYLRLTKQVFSNLEDTAMAIAEDKKKLVRTDPDVERVRRCHLNDLESIVPFVVIGLLYALTGPVLSTALLHFRVFVVSRFIHTVAYIMALPQPTRGVAFGVGLLTTLSMAYRVLTTALFL

>Danio rerio(B0R1F0)

MAEVVHMIDSEVFLAFSTYATIVILKMMLMSLMTSYLRLTKQVFSNLEDTAMAIAEDKKKLVRTDPDVERVRRCHLNDLESIVPFVVIGLLYALTGPVLSTALLHFRVFV

>Pongo abelii(A0A2J8XP37)

MAGNSILLAAVSILSACQQSYFALQVGKARLKYKVTPPAVTGSPEFERVFRAQQNCVEFYPIFIITLWMAGWYFNQVFATCLGLVYIYGRHLYFWGYSEAAKKRITGFRLSLGILALLTLLGALGIANSFLDEYLDLNIAKKLRRQF

>Canis lupus familiaris(F1PFR5)

IFSCRQNCVEFYPIFLVTLWMAGWYFNQVFATCLGLVYIYARHQYFWGYSEAAKKRITGFRLSLGCLALLTVLGALGIANSFLDEYLDLNVIKKLR

>Equus caballus(F7AUV5)

MAGNSILLAAVSLLSACQQSYFAFQVGRARLKYKIMPPAVSGSPEFDRIFRAQQNSVEFYPAFMITLWMAGWYFNQVFATCLGLLYIYARHQYFWGYSEAANKRMTGFRLGLGILALLAILGALGIANSFLDEYLDLNVAKKLRHF

>Canis lupus familiari(E2RHK1)

MVDLTELMENEVFMAFASYTTIILSKMMFMSTATAFFRLTRKVFANPEDCASFGKGENAKKYLRTDDRVERVRRAHLNDLENIVPFLGIGLLYSLSGPDLSTALLHFRLFVGARIYHTIAYLTPLPQPNRALAFFIGYGVTFSMAYRLLKSRLYL

>Ornithorhynchus anatinus(F6QVY1)

MADDLILLAVVSVLSACQQTYFAWQVGKARFKYKIMPPAVSGSPEFERIYRAHQNCVECYPVFLTTFWIAGWYFNQELVAILGLGYMYARHQYFYGYSEAVKRRIKGFRLTVGILTLLVVLSAVGIANRFLDEYVDFSLSKKIRRLF

>Homo sapiens(P10620)

MVDLTQVMDDEVFMAFASYATIILSKMMLMSTATAFYRLTRKVFANPEDCVAFGKGENAKKYLRTDDRVERVRRAHLNDLENIIPFLGIGLLYSLSGPDPSTAILHFRLFVGARIYHTIAYLTPLPQPNRALSFFVGYGVTLSMAYRLLKSKLYL

>Rattus norvegicus(P08011)

MADLKQLMDNEVLMAFTSYATIILAKMMFLSSATAFQRLTNKVFANPEDCAGFGKGENAKKFLRTDEKVERVRRAHLNDLENIVPFLGIGLLYSLSGPDLSTALIHFRIFVGARIYHTIAYLTPLPQPNRGLAFFVGYGVTLSMAYRLLRSRLYL

>Mus musculus(Q91VS7)

MADLRQLMDNEVLMAFTSYATIILTKMMFMSSATAFQRITNKVFANPEDCAGFGKGENAKKFVRTDEKVERVRRAHLNDLENIVPFLGIGLLYSLSGPDLSTALMHFRIFVGARIYHTIAYLTPLPQPNRGLAFFVGYGVTLSMAYRLLRSRLYL

>Sus scrofa(P79382)

MADLTELMKNEVFMAFASYATIVLSKMMFMSTATAFYRLTRKVFANPEDCSSFGKGENAKKYLRTDERVERVRRAHLNDLENIVPFLGIGLLYSLSGPDLSTAILHFRLFVGARIYHTIAYLTPLPQPNRGLAFFLGYGVTLSMAYRLLKSRLYL

**Mu Class GSTs**

>Chlamys islandica(Q0P7I5)

AQQLRLMLQYGGVEYEDKRYELQKGTDGSYKCPEWFEQDKKTLKLDLPNLPYLIDGSTELTETDAIALYLAEKLKLTGSSEKEKHLAHMTNLRIHDFRLAIIKVVYSPEHEALKGELFASFPERLALFSDFLGPKKKWLVGDSITFADFNFYDLLDILEVYVPTCLDEFPPLQRFIERFEALPKIKKYLASEQHQAVKNQPNNKSAYMGNSYVK

>Gallus gallus(P20136)

MVVTLGYWDIRGLAHAIRLLLEYTETPYQERRYKAGPAPDFDPSDWTNEKEKLGLDFPNLPYLIDGDVKLTQSNAILRYIARKHNMCGETEVEKQRVDVLENHLMDLRMAFARLCYSPDFEKLKPAYLEQLPGKLRQLSRFLGSRSWFVGDKLTFVDFLAYDVLDQQRMFVPDCPELQGNLSQFLQRFEALEKISAYMRSGRFMKAPI

>Mus musculus(O35660)

MPVTLGYWDIRGLGHAIRLLLEYTETGYEEKRYAMGDAPDYDRSQWLNDKFKLDLDFPNLPYLIDGSHKVTQSNAILRYLGRKHNLCGETEEERIRVDILEKQVMDTRIQMGMLCYSADFEKRKPEFLKGLPDQLKLYSEFLGKQPWFAGDKITFADFLVYDVLDQHRMFEPTCLDAFPNLKDFMARFEGLRKISAYMKTSRFLPSPVYLKQATWGNE

>Rattus norvegicus(P08010)

MPMTLGYWDIRGLAHAIRLFLEYTDTSYEDKKYSMGDAPDYDRSQWLSEKFKLGLDFPNLPYLIDGSHKITQSNAILRYLGRKHNLCGETEEERIRVDVLENQAMDTRLQLAMVCYSPDFERKKPEYLEGLPEKMKLYSEFLGKQPWFAGNKITYVDFLVYDVLDQHRIFEPKCLDAFPNLKDFVARFEGLKKISDYMKSGRFLSKPIFAKMAFWNPK

>Mesocricetus auratus(P86214)

SMVLGYWDIRRMLLEFTDTSYEEKRYICGEAPDYDRSQWLDVKFKLDLDFPNLPYLMDGKNKITQSNAILRIRVDIMENQIMDFRQFSLFLGKKLTFVDFLTYDVLDQNRMFEPKCLDEFPNLKAFMCRCFKMPINNK

>Pongo abelii(Q5R8E8)

MPIILGYWNIRGLAHSIRLLLEYTDSSYEEKKYMMGDAPDYDRSQWLNEKFKLGLDFPNLPYLIDGTHKITQSNAILRYIARKHNLCGETEKEKIQEDILENQLMDNRMQLARLCYNPDFEKLKPEYLEGLPEMLKLYSQFLGKQPWFLGDKITFVDFIAYDVLERNQVFEPSCLDAFPNLKDFISRFEGLEKISAYMKSSRFLPRPVFTKMAVWGNK

>Macaca fuscata fuscata(Q9BEB0)

MPMTLGYWNIRGLAHSIRLLLEYTGSSYEEKKYTMGDAPDYDRSQWLNEKFKLGLDFPNLPYLIDGTHKITQSNAILRYIARKHNLCGETEKEKIREDILENQLMDNRMQLARLCYDPDFEKLKPEYLEGLPEMLKLYSQFLGKQPWFLGDKITFVDFIAYDVLERNQVFEPSCLDAFPNLKDFISRFEGLEKISAYMKSSRFLPRPVFTKMAVWGNK

>Macaca fascicularis(Q9TSM5)

MPMTLGYWDIRGLAHAIRLLLEYTDSSYEEKKYTMGDAPDYDRSQWLNEKFKLGLDFPNLPYLIDGTHKITQSNAILRYIARKHNLCGETEEEKIRVDILENQAMDVSNQLARVCYSPDFEKLKPEYLEGLPTMMQHFSQFLGKRPWFVGDKITFVDFLAYDVLDLHRIFEPKCLDAFPNLKDFISHFEGLEKISAYMKSSRFLPKPLYTRVAVWGNK

>Schistosoma mansoni(P15964)

MAPKFGYWKVKGLVQPTRLLLEHLEETYEERAYDRNEIDAWSNDKFKLGLEFPNLPYYIDGDFKLTQSMAIIRYIADKHNMLGACPKERAEISMLEGAVLDIRMGVLRIAYNKEYETLKVDFLNKLPGRLKMFEDRLSNKTYLNGNCVTHPDFMLYDALDVVLYMDSQCLNEFPKLVSFKKCIEDLPQIKNYLNSSRYIKWPLQGWDATFGGGDTPPK

>Schistosoma japonicum(P26624)

VKLIYFNGRGRAEPIRMILVAAGVEFEDERIEFQDWPKIKPTIPGGRLPIVKITDKRGDVKTMSESLAIARFIARKHNMMGDTDDEYYIIEKMIGQVEDVESEYHKTLIKPPEEKEKISKEILNGKVPILLQAICETLKESTGNLTVGDKVTLADVVLIASIDHITDLDKEFLTGKYPEIHKHRKHLLATSPKLAKYLSERHATAF

>Schistosoma haematobium(P30114)

MTGDHIKVIYFNGRGRAESIRMTLVAAGVNYEDERISFQDWPKIKPTIPGGRLPAVKITDNHGHVKWMLESLAIARYMAKKHHMMGETDEEYYNVEKLIGQVEDLEHEYHKTLMKPEEEKQKITKEILNGKVPVLLDIICESLKASTGKLAVGDKVTLADLVLIAVIDHVTDLDKEFLTGKYPEIHKHRENLLASSPRLAKYLSDRAATPF

>Schistosoma japonicum(P08515)

MSPILGYWKIKGLVQPTRLLLEYLEEKYEEHLYERDEGDKWRNKKFELGLEFPNLPYYIDGDVKLTQSMAIIRYIADKHNMLGGCPKERAEISMLEGAVLDIRYGVSRIAYSKDFETLKVDFLSKLPEMLKMFEDRLCHKTYLNGDHVTHPDFMLYDALDVVLYMDPMCLDAFPKLVCFKKRIEAIPQIDKYLKSSKYIAWPLQGWQATFGGGDHPPK

>Sarcoptes scabiei(Q2YFE9)

MATRTTDSNSNDSKLPILAYWNIRGNAQPIRLLLRYTKTPYKEKSYNFGKYEQDKAIWRADKPHLGLDFPNLPYYIDGDLRLTQSLTILRYLAKKHHLAGINETERIRIDLMEQQLRDFRNQFIDATNDANFEKARVIYLARLPEKLQSLSNFLKDRPFFAGNSISYVDFMAYEFIDQHYYLNPDLFGQNQQWRNLIDFLHRIESFPTIKEYQYSEDYIRHPSGLLIAWYEAKFFSTFNRSLGDQPSEQLRKEFIRSEMVSN

>Rattus norvegicus(P04905)

MPMILGYWNVRGLTHPIRLLLEYTDSSYEEKRYAMGDAPDYDRSQWLNEKFKLGLDFPNLPYLIDGSRKITQSNAIMRYLARKHHLCGETEEERIRADIVENQVMDNRMQLIMLCYNPDFEKQKPEFLKTIPEKMKLYSEFLGKRPWFAGDKVTYVDFLAYDILDQYHIFEPKCLDAFPNLKDFLARFEGLKKISAYMKSSRYLSTPIFSKLAQWSNK

>Conorchis sinensis(Q25595)

MAPVLGYWKIRGLAQPIRLLLEYVGDSYEEHSYGRCDGEKWQNDKHNLGLELPNLPYYKDGNFSLTQSLAILRYIADKHNMIGNTPVERAKISMIEGGLVDLRAGVSRIAYQETFEQLKVPYLQQLPSTLRMWSQFLGNNSYLHGSTPTHLDFMFYEALDVIRYLDPTSVEAFPNLMQFIHRIEALPNIKAFMESDRFIKWPLNGWSAYFGGGDAPPK

>Cyphoma gibbosum(A7LFK1)

MPTLAYWKIRGLAQPMRLLLNYAGEKFEDKQYEQGDAPGFSVEEWTKEKFSLGLDFPNLPYYIDGDIKITQSNAILRYIANKHNLMGKTPKEKAQVDMMLENAMDLRNGVVKMAYNKDYEKMIKEYEPKCKEILAGYEKWLSSHKWFGCDTVTVADFPMYELLDQHRLMIKDVLVPYPNITKFMAAFEALPAIKAYMASDKFMKRPINNKIASFK

>Cyphoma gibbosum(A7LFK0)

MPTLGYWKTRGIGQPIRLLLNYVKEEFDDVYYEMGDAPDYSRDAWLNVKYTLGMNFPNLPYYIDGDVKVSQSNAILRYIARKHDLLGKTDEEKTNVDMMLDIAMDMRNGVIKMVYNKDYEKLIKEYEPKCKEILAGYEKWLSSHKWFGGDDVTVADFHMYELLDQHRLMIKDVLVPYPNITKFMAAFEALPAIKAYMASDKFMKQPINNKFASFI

>Dermatophagoides pteronyssinus(P46419)

MSQPILGYWDIRGYAQPIRLLLTYSGVDFVDKRYQIGPAPDFDRSEWLNEKFNLGLDFPNLPYYIDGDMKMTQTFAILRYLGRKYKLNGSNDHEEIRISMAEQQTEDMMAAMIRVCYDANCDKLKPDYLKSLPDCLKLMSKFVGEHAFIAGANISYVDFNLYEYLCHVKVMVPEVFGQFENLKRYVERMESLPRVSDYIKKQQPKTFNAPTSKWNASYA

>Dermatophagoides pteronyssinus(Q2YFE4)

MNKPTLGYWDLRGLGQPIRLMLAYAGVDYVDKRYTLGPDMDRSEWLKDKFNLGLDFPNLPYYIDGDVKMTQSMAILRYLARKYNMDGSNEQERVRISMAEQQVYDMFMAMVRVCYDPNMEKLRVDYLKTLPDSLKLMSKFMANHDFIAGSKISYADFYLYEYMCRIKVMVPEVYGQFENLKKFVERFESLPRVSDYIKKQTPKTFNAAMAKWNGSYP

>Dermatophagoides pteronyssinus(Q2YFE5)

MSQPILGYWDIRGYAQPIRLLLTYSGVDFVDKRYQIGPAPDFDRSQWLNEKFNLGLDFPNLPYYIDGDMKMTQTFAILRYLGRKYKLNGSNDHEEIRISMAEQQTKDMMAAMIRVCYDANCDKLKPDYLKSLPDCLKLMSKFVGEHPFVAGANISYVDFYLYEYLCRVKVMVPEVFGQFENLKRYVERMESLPRVSDYIKKQQPKTFNAPTSKWNASYA

>Echinococcus granulosus(O16058)

MAPTLAYWDIRGLAEQSRLLLKYLEVEYDDKRYKIGSTPTFDRSAWLSEKFSLGLDFPNLPYYIDGDFKLTQSGAILEYIADRHGMIPDCKKRRAVLHMLQCEVVDLRMAFTRTCYSPDFEKLKPGLFETLAQKLPNFEAYLGEKEWLTGDKINYPDFSLCELLNQLMKFEPTCLEKYPRLKAYLSRFENLPALRDYMASKEFKTRPCNGASAKWRGDC

>Fasciola hepatica(P56598)

MPAKLGYWKIRGLQQPVRLLLEYGEKYEEQIYERDDGEKWFSKKFELGLDLPNLPYYIDDKCKLTQSLAILRYIADKHGMIGSTPEERARVSMIEGAAVDLRQGLSRISYDPKFEQLKEGYLKDLPTTMKMWSDFLGKNPYLRGTSVSHVDFMVYEALDAIRYLEPHCLDHFPNLQQFMSRIEALPSIKAYMESNRFIKWPLNGWHAQFGGGDAPPSHEKK

>Homo sapiens(P09488)

MPMILGYWDIRGLAHAIRLLLEYTDSSYEEKKYTMGDAPDYDRSQWLNEKFKLGLDFPNLPYLIDGAHKITQSNAILCYIARKHNLCGETEEEKIRVDILENQTMDNHMQLGMICYNPEFEKLKPKYLEELPEKLKLYSEFLGKRPWFAGNKITFVDFLVYDVLDLHRIFEPKCLDAFPNLKDFISRFEGLEKISAYMKSSRFLPRPVFSKMAVWGNK

>Homo sapiens(P28161)

MPMTLGYWNIRGLAHSIRLLLEYTDSSYEEKKYTMGDAPDYDRSQWLNEKFKLGLDFPNLPYLIDGTHKITQSNAILRYIARKHNLCGESEKEQIREDILENQFMDSRMQLAKLCYDPDFEKLKPEYLQALPEMLKLYSQFLGKQPWFLGDKITFVDFIAYDVLERNQVFEPSCLDAFPNLKDFISRFEGLEKISAYMKSSRFLPRPVFTKMAVWGNK

>Homo sapiens(P46439)

MPMTLGYWDIRGLAHAIRLLLEYTDSSYVEKKYTLGDAPDYDRSQWLNEKFKLGLDFPNLPYLIDGAHKITQSNAILRYIARKHNLCGETEEEKIRVDILENQVMDNHMELVRLCYDPDFEKLKPKYLEELPEKLKLYSEFLGKRPWFAGDKITFVDFLAYDVLDMKRIFEPKCLDAFLNLKDFISRFEGLKKISAYMKSSQFLRGLLFGKSATWNSK

>Homo sapiens(Q03013)

MSMTLGYWDIRGLAHAIRLLLEYTDSSYEEKKYTMGDAPDYDRSQWLNEKFKLGLDFPNLPYLIDGAHKITQSNAILCYIARKHNLCGETEEEKIRVDILENQAMDVSNQLARVCYSPDFEKLKPEYLEELPTMMQHFSQFLGKRPWFVGDKITFVDFLAYDVLDLHRIFEPNCLDAFPNLKDFISRFEGLEKISAYMKSSRFLPKPLYTRVAVWGNK

>Homo sapiens(P21266)

MSCESSMVLGYWDIRGLAHAIRLLLEFTDTSYEEKRYTCGEAPDYDRSQWLDVKFKLDLDFPNLPYLLDGKNKITQSNAILRYIARKHNMCGETEEEKIRVDIIENQVMDFRTQLIRLCYSSDHEKLKPQYLEELPGQLKQFSMFLGKFSWFAGEKLTFVDFLTYDILDQNRIFDPKCLDEFPNLKAFMCRFEALEKIAAYLQSDQFCKMPINNKMAQWGNKPVC

**Omega Class GSTs**

>Bombyx mori(A8R5V3)

MSAIKDSRNINFNTKHLRKGDPLPPFNGKLRVYNMRYCPYAQRTILALNAKQIDYEVVNIDLIDKPEWLTTKSAFAKVPAIEIAEDVTIYESLVTVEYLDEVYPKRPLLPQDPLKKALDKIIVEASAPIQSLFIKILKFSDTVNEEHVAAYHKALDFIQEQLKNRGTVFLDGSEPGYADYMIWPWFERLRAFAHDERVRLEPSKYSLLLEYIDNMLKDSAVSQYLIPLEILAKFHEAYTKKERPNYELLNECLKSF

>Saccharomyces cerevisiae(P48239)

CPFTHRAILARSLKKLEPVLGLVLSHWQLDSKGARFLPAPHRPEKYKERFFTATGGIASAKLDESEELGDVNNDSARLFVDGAFDPVENISRLSELYYLNDPKYPGTKFTVPVLWDSKTRKIVNNESGDIIRILNSGVFDEFIQSEETNVIDLVPHDLIDEIDKNIKWVHPKINLGVYKVGLAENGKIYETEVKTLFENLQKMECVLKENYKRLEEQFSGNKQKILAKYFVLGQRLTEADIRLYPSIIRFDVVYVQHFKCNLKTIRDGFPYLHLWLINLYWNYAEFRFTTDF

>Schizosaccharomyces pombe(O94524)

DRYHLYVSYACPWAHRTLIVRKLKGLENVIPVHVVGWLMGPNGWNFDKENDSTGDPLYNSPYLRNLYFRADPNYNMRFTVPVLWDSKYNTIVNNESAEIIRMFNDAFNEVIEDEEKRVVDLYPSSLRTKIDELNDYFYDTVNNGVYKTGFATTAEAYEKNVRVVFQGLDRLEQVLKESKGPFLLGDHLTETDVRLYTTIVRFDPVYVQHFKCNIGTIRHNYPHINQWLKRLYWKHPAFHETTDFKHIKCHYT

>Aspergillus ruber(A0A017SRP4)

MPPPDADLYPEASGAAKALVEEHSVEQPLKLYAGWFCPFVQRVWLALEEKQIPYQYIEVNPYHKSQSLLSLNPRGLVPTLSVSHSGISKPLYESTVILEYLEEAYPDHKPCLLPEDPYERARVRIWVDYVTSRIIPAFHRFLQYQEGQSSSSIDTLRNEFLNHLKEWTKEAHPDGPFFLGKDVSIPDLVLSPWAIRLWIFDEFKDGGLGIPFEGQGGEDESVWSRWRKWLAAIEARPSIQQTTSEKEFYIPIYKRYADNTAESELAKATRTGRGVP

>Aedes albopictus(A0A023EL34)

MSNGKHLAKGSTPPVLGNDGKLRLYSMRFCPYAQRVHLILDAKNIAYHTIYINLSEKPEWYFDKNPLGKVPALEVPGKENITLYESLVVADYIEEAFPDKQRKLYPSDPFKKAQDRILIERFNGAVISPYYRILFSSDGIPPGAITEFGTGLDIFETELKNRGTSYYGGDKPGMLDYMIWPWCERVDLLKFALGDKYELDKQRFGKLLQWRDLMEKDDAVQKSFLSTENHTKFLQSRKSGENNYDILS

>Amblyomma triste(A0A023GGV7)

MSAWALETGSKLPPLVPGKLRLYSMRFCPYAQRALLMLKAKGVDHEVVNVSLRNRPEWYNEVLPAGTVPVLYQDEKVISGSMPIAEYLEEAYPEPHLLPTDPYLKALDRSFLDVALPCAGLISSISLNKGVKEEHWANFLKKIEAFDKELAKRKTKFFGGEKPGLVDYVVWPAFPAALAYSKLYPDLKMPAAEQFPHFSRWLQAMREQPVVKAVVNEDHVLLYAKSGIDGDRDFNAGLK

>Amblyomma triste(A0A023GK27)

MSAWALETGSKLPPLVPGKLRLYSMRFCPYAQRALLMLKAKGVDHEVVNVSLRNRPEWYNEVLPAGTVPVLYQDEKVISGSMPIAEYLEEAYPEPHLLPTDPYLKALDRSFLDVALPCAALISSISLNKGVKEEHWANFLKKIEAFDKELANRKTTFFGGEKPGLVDYVVWPAFPAALAYSKLYPDLKMPAAEQFPHFSRWLQAMREQPVVKAVVNEDHVLLYAKSGIDGDRDFNAGLQ

>Macaca fascicularis(A0A023JBX8)

MSQDATRTLGKGSQPPGPVPEGLIRIYSMRFCPYSHRTRLVLKAKDIRHEVVNINLRNKPEWYYTKHPFGHIPVLETSQCQLIYESVIACEYLDDAYPGRKLFPHDPYERARQKMLLELFCKVPHLTKECLVALRCGRECTDLKASLRQEFCNLEEILEYQNTTFFGGTCTSMIDYLLWPWFERLDVYGIADCVSHTPALRLWISAMKWDPTVCALLTDKSIFQGFLNLYFQNNPNAFDFGLC

>Rattus norvegicus(Q9Z339)

MSGASARSLGKGSAPPGPVPEGQIRVYSMRFCPFAQRTLMVLKAKGIRHEIININLKNKPEWFFEKNPFGLVPVLENTQGHLITESVITCEYLDEAYPEKKLFPDDPYEKACQKMTFELFSKVPSLVTSFIRAKRKEDHPGIKEELKKEFSKLEEAMANKRTAFFGGNSLSMIDYLIWPWFQRLEALELNECIDHTPKLKLWMATMQEDPVASSHFIDAKTYRDYLSLYLQDSPEACDYGL

>Sus scrofa(Q9N1F5)

MSGGSARSLGKGSAPPGPVPEGLIRVYSMRFCPFAQRTLLVLNAKGIRHQVININLKNKPEWFFQKNPSGLVPVLENSQGQLIYESAITCEYLDEAYPGKKLLPDDPYEKACQKMVFELSSKVPPLLIRFIRRENEADCSGLKEELRKEFSKLEEVLTKKKTTYFGGSSLSMIDYLIWPWFERLEALELNECIDHTPKLKLWMAAMMKDPAVSALHIEPRDLRAFNDLYLQNSPEACDYGL

>Phanerochaete chrysosporium(J7JYU5)

MAQKEQITFYTHFYSPYCDRVHLALEEVKADYTVYTVDVMNKPKWYTEQINPVGKIPAITYGGPKVKPEEPSPESCKLRESLVILEFLADLFPEAGLLPTDPVLRAKARLFASDVDAHVFEGFKAYFFMREPASKLLDALDRFQQQLPARGFAVGDKWTLADMAAAPFLVRTYLLLEHDLGVYPAGEGPKTLALLRGERFARLNQYLADLRAQPSFKATWDEAAQVAIWKSNPMFKRE

>Oncorhynchus kisutch(L7QHV5)

MASEKCFAKGSSAPGLVAKGQIRLYSMRFCPFAHRTRLVLHAKGIKHDTVNINLKDKPEWFLKKNPLGLVPTLETSSGQVIYESPITCDYLDEVYTDKKLLPADPFQKAQQKMMLENFSKVTPYFYKIPMGKQNGEDISVLEGELKEKFVKLNEDLVNKKSKFFGGNAITMIDYMMWPWFERLEIFELKHCLDGTPELKKWTEHMSEDQTVKATMFPTETYKAFYKTYADGKPNYDYGL

>Apis mellifera(A0A088A0S0)

MSSKHLTIGSVAPPIVPGKIRLYSMRFCPYAQRIHLVLDAKHIPHDVVYVNLTHKPDWLLEKSPLGKVPCIELEGGEILYESLVIAEYLDDTYPQNKLYPNDPLARAKDKLLIGRFNSVINTMCKLFINTSIDQDIFDEALSELELFERELASRGTPFFHGNSPGMLDFMIWPWWERSNTIKMLRGDQFTIPHDRFKRLLEWRSAMKENPAIRSNYLDTEIHAKYMQSRRAGTPQYDLITD

>Drosophila melanogaster(Q9VSL5)

MALPQKHFKRGSTKPELPEDGVPRFFSMAFCPFSHRVRLMLAAKHIEHHKIYVDLIEKPEWYKDFSPLGKVPALQLTGVKDQPTLVESLIIAEYLDQQYPQTRLFPTDPLQKALDKILIERFAPVVSAIYPVLTCNPNAPKDAIPNFENALDVFEVELGKRGTPYFAGQHIGIVDYMIWPWFERFPSMKINTEQKYELDTKRFEKLLKWRDLMTQDEVVQKTALDVQLHAEFQKSKTLGNPQYDIAFKGTP

>Homo sapiens(P78417)

MSGESARSLGKGSAPPGPVPEGSIRIYSMRFCPFAERTRLVLKAKGIRHEVININLKNKPEWFFKKNPFGLVPVLENSQGQLIYESAITCEYLDEAYPGKKLLPDDPYEKACQKMILELFSKVPSLVGSFIRSQNKEDYAGLKEEFRKEFTKLEEVLTNKKTTFFGGNSISMIDYLIWPWFERLEAMKLNECVDHTPKLKLWMAAMKEDPTVSALLTSEKDWQGFLELYLQNSPEACDYGL

>Homo sapiens(Q9H4Y5)

MSGDATRTLGKGSQPPGPVPEGLIRIYSMRFCPYSHRTRLVLKAKDIRHEVVNINLRNKPEWYYTKHPFGHIPVLETSQCQLIYESVIACEYLDDAYPGRKLFPYDPYERARQKMLLELFCKVPHLTKECLVALRCGRECTNLKAALRQEFSNLEEILEYQNTTFFGGTCISMIDYLLWPWFERLDVYGILDCVSHTPALRLWISAMKWDPTVCALLMDKSIFQGFLNLYFQNNPNAFDFGLC

>Danaus plexippus(A0A212ENR8)

MSEKHLQSGDSLPPFTGKLRLFAMRFCPYAERSILCLNAKKLQYDLVFINLDHKPEWIFQFNPKGAVPALEYEEGKAIFDSNVINVYLDEKYPEIPLQNSDPLRRAQDKLLVEMFAGAQSAYYTAAFNPQAVEPSMVENFHKGLDLLQKEIESRGTKFLNGDEPGLVDYTIWPFLERFEALPILGQQEFAIDKSKYEILITYMAAMRDSPAVKAYALAPDTHAKFTESRIKGDANYNMLDTSAVCCMRPRKKKE

>Danaus plexippus(A0A212ENT1)

MSSKAITGKINFNTKHLKRGDPLPPYNGKLRVYNMRYCPFAQRTILALNAKQMDYEVVNINLMDKPEWLTRKSAFGKVPAIEINEDVCIFESLVTVEYLDEAYPQRPLLPKDPLRKALDKILIEASGPIHTMMFKTVKMPDSITEDNLKAYESSLQYIQNELINRKTKFLSGNEPGYVDYMIWPWFERIGALKKFDERAGIDSSKFGLLLEYCSNMAKDPAVSDYLLPDDILFKYFEGYKAGAPNYELITEE

>Danaus plexippus(A0A212ENC3)

MAYYPHRTAGPTPPPPLTDKLRLYHVDMNPYGHRVLLILEAKRAKYEVYRLDPLNLPQWFKTKNPRLKIPVLEIPTDQGDKYLFESVVICDYLDEKYPRNPLHSRDPYVKAQDRLLIERFNELIKGSLECFDTNFAFGGEQIFQTLDIFEKELASRGTNYFGGHSPGMLDYMIWPWVERLYLLRCVNERKFDEKRTLFPNFADWGDQMQLDDVVKRHSNSPSEYFAYYRNARTHSMGYYL

>Plutella xylostella(255AA)

MSEKHLQTGDALPPFGGKLRLFAMRFCPYAERSVLVLNAKNIPYDLVFINLDQKPEWIFNFSPRGAVPALEYEQGKGIFDSNVINVYLDEKYPEVPLQAADPLRRAQDKLIVENFSAAQSAYYTAAFNAQALQPSHLENYHKGLELLQKELETRGTKFLHGDQAGLVDYTLWPFLERFEALPLLGKSEYAIDKSKYDILLTYMESMKQVPAVKTYYLSADMHAKFTESRVKGDPNYNMLDSSAEVCCFRPRKKKE

>Plutella xylostella(249AA)

MSTRGIKFNTKHLRKGDPLPPYNGKLRLYNMRYCPFAQRTVLALNAKDIDYEVVNINLFEKPEWLTSKSAFGKVPSLEIKEGLSIYESLVTVEYLDEVYPQRPLLPKDPVQRALDKIIVEACTPIQGLFIKLIKFPESISEDTVAAYHKALHFLQEQLQSRGTRFFGGDQPGFVDYMIWPWFERVLPYQKVESRVQIDAGKFKLLLEYLQNLKQDPVVKQYLIEDEVLFKFLEPYKTGGEPNYDLLLEA

>Plutella xylostella(241AA)

MSFYYQERPAGPTPPGPLSNKLRLYHVDMNPYGHRVLLILDAKKVPYEVCKLDPLRLPEWFREKNPRLKIPVLEIPTDQGDKYLFESIVICDYLDERYTRNPLHSRDPFVKAQDRLLIERFNELIKGSLECFDTNFAFGNEQIIQTVNIFEKELESRGTVYFGGDRPGMLDYMIWPWIERLYMLRCLNPTKFDEKRHIFPNFADWGDQMQLDEVVKKHASSPEDNFEYYKNARAHSMGYYL

>Drosophila melanogaster(Q9VSL4)

MALPQKHFKRGSPKPEIPEDGVLRYYSMRFCPYSQRAGLVLAAKKIPHHTVYIDLSEKPEWYIDYSPLGKVPAIQLPNLPGQPALVESLVIAEYLDEQYPGEGSLFPKDPLQKALDRILIERLSPAVSAIYPVLFTKNPPADAIKNFETALDVFEQEITKRGTPYFGGNKIGIADYMIWPWFERFPALKYTLDEPYELDKTRYQNLLKWRDLVAQDEAVKATALDARIHAKFMKTRHENKPDYDVAFQPL

>Drosophila melanogaster(Q9VSL6)

MSNTQHLTIGSPKPVFPDDGILKLYSMRFCPYAHRVHLVLDAKKIPYHAIYINLRDKPEWFSLVSSSTKVPALELVKEQGNPVLIESLIICDYLDEKYPEVPLYPKDLLKKAQEKILIERFGQFINAFYYLLLHDNPEQLVDTDHYAGLVVYEEELKRRCTKFFGGDSPGMLDYMMWPWCERFDSLKYTFEQKFELSPERFPTLIKWRDLMIQDRAVKCFYLDGQTHAKYMNSRRSGQADYNMLYNEAKRVKLG

>Bombyx mori(Q2F689)

MSEKHLQTGDVLPPYSGKLRVFAMRFCPYAERTVLTLNAKNIPYDLVFINLDQKPEWIFNFSPKGTVPALEYEPGKALFDSNIINVYLDEKYPEIPLQASDPLRRAQDKILVESFAPAQSAYYTAAFNAQALEPSMVETYHKGLEGLQKELETRSTKYLHGDEPGWVDYTLWPFLERFEALPLIGKAEFAIDQTKYERLVTYIEAMKNVPAVKSYFLAAETHAKFIESRAQGDANYNMLDTSAVCCMRPRKKKE

>Bombyx mori(Q2I0J6)

MSAIKDSRNINFNIKHLRKGDPLPPFNGKLRVYNMRYCPYAQRTILALNAKQIDYEVVNIDLIDKPEWLTTKSAFAKVPAIEIAEDVTIYESLVTVEYLDEVYPKRPLLPQDPLKKALDKIIVEASAPIQSLFIKILKFSDTVNEEHVAAYHKALDFIQEQLKNRGTVFLDGSEPGYADYMIWPWFERLRAFAHDERVRLEPSKYSLLLEYIDNMLKDSAVSQYLIPLEILAKFHEAYTKKERPNYELLNECLKSF

>Bombyx mori(Q1HPV9)

MTYFHSVNAGVIPPPALTDKLRLYHVDMNPYGHRVLLVLEAKRIKYEVYRLDPLRLPEWFRAKNPRLKIPVLEIPTDQGDRFLFESVVICDYLDEKYTRHTLHSHDPYVKAQDRLLIERFNELIKGSLECFDTNFAFGSEQIIQTLEIFEKELTNRGTNYFGGNRPGMLDYMVWPWVERLYLLRCVNDRKFVEKKSLFPNFADWGDQMQLDDIVKKHAHSPQEYFDYYKNARAHSMGYYL

>Bombyx mori(B0LKP6)

MVSPKINFNTKHLGKGDPLPPWSGKLRVYNMRLCPFAQRTILTLNAKQIDYEVINIDLVNKPEWLPTKSIFGKVPTIEVEDGVCICESLIIAEYLEEVYPEIPLISKDPIKKAYEKIIIEASEPIFVMYFKVMRTPDTINDETLMSYHKALTFFEGQLRNRGTRFLGGEKPGFADYMIWPWFERIQSMNDEKLKIKSAKFDLLVAYIENMYKDPAVSQYLLPKDVMDKLHAEYKTGKFEVQSIEDLL

>Caenorhabditis briggsae(A8XT16)

MPVLAGINSKVLKNGDSEPSPPPAGIYRIYNMRFCPWAQRALIYASVKNVPSEVINIHLKEKPDWYFSKHYKGQVPALELDEGKKHVIESAHIPEYLDDLFPESRILPSDPYEKVQQKLLLERLAAVAPAFYAAAQAANNPEGRDEKYAALVKAFEDAEKLLTGDFFSGKAKPGFADYLIFPNYQRVFWLSHILPNSPFSSESFPGPNFPKLAKWYRTLDSIPEVAAASQPTEMGVGFFNDYLKGTPNYDYGL

>Mus musculus(O09131)

MSGESSRSLGKGSAPPGPVPEGQIRVYSMRFCPFAQRTLMVLKAKGIRHEVININLKNKPEWFFEKNPLGLVPVLENSQGHLVTESVITCEYLDEAYPEKKLFPDDPYKKARQKMTLESFSKVPPLIASFVRSKRKEDSPNLREALENEFKKLEEGMDNYKSFLGGDSPSMVDYLTWPWFQRLEALELKECLAHTPKLKLWMAAMQQDPVASSHKIDAKTYREYLNLYLQDSPEACDYGL

>Caenorhabditis elegans(P34345)

MVLTGVTSKAIRKGDAEPPLSKGSFRVYNMRFCPWAERAMLYVAAKGIEAEVVNLNVTDKLEWYWTKHYQGKAPAVEHNGKVVIESGFIPEYLDDAFPETRILPTDPYEKVQQKLLADRLTAVAHAVPLLFAVMRDRTLKDEKQRKVFEVLKQAENLLANDFYAGSQPGYPDYLSFPFFEKIWWSASLDGVVDLPTIEFPGEEEYPKLTKWFQKMISSDVVQSVTQSLEHGAAFMNAYATHQELNYDLGL

>Rattus norvegicus(Q6AXV9)

MSGDLTRCLGKGSCPPGPVPEGVIRIYSMRFCPYSHRTRLVLKAKSIRHEIININLKNKPDWYYTKHPFGQVPVLENSQCQLIYESVIACEYLDDVFPGRKLFPYDPYERARQKMLLELFCKVPQLSKECLVALRCGRDCTDLKVALRQELCNLEEILEYQNTTFFGGDSISMIDYLVWPWFERLDVYGLADCVNHTPMLRLWISSMKQDPAVCALHIDKNIFLGFLNLYFQNNPCAFDFGLCGPIVR

**Pi Class GSTs**

>Homo sapiens(P09211)

MPPYTVVYFPVRGRCAALRMLLADQGQSWKEEVVTVETWQEGSLKASCLYGQLPKFQDGDLTLYQSNTILRHLGRTLGLYGKDQQEAALVDMVNDGVEDLRCKYISLIYTNYEAGKDDYVKALPGQLKPFETLLSQNQGGKTFIVGDQISFADYNLLDLLLIHEVLAPGCLDAFPLLSAYVGRLSARPKLKAFLASPEYVNLPINGNGKQ

>Mus musculus(P19157)

PPYTIVYFPVRGRCEAMRMLLADQGQSWKEEVVTIDTWMQGLLKPTCLYGQLPKFEDGDLTLYQSNAILRHLGRSLGLYGKNQREAAQMDMVNDGVEDLRGKYVTLIYTNYENGKNDYVKALPGHLKPFETLLSQNQGGKAFIVGDQISFADYNLLDLLLIHQVLAPGCLDNFPLLSAYVARLSARPKIKAFLSSPEHVNRPI

>Dirofilaria immitis(P46426)

MSYKLTYFPIRGLAEPIRLLLVDQGIKFTDEHIPKDDFVSIKSQFQFGQLPCFYDGDQQIVQSGAILRHLARKFNLNGENNAETSYVDMFYEGIRDLHSKYTRMIYEAYETQKDPFIKNILPQELAKLEKLLATRDNGKNFILGDKISFADYVLFEELDVQQILDPHCLEKFPLLKAFHQRLGDKPKIKEYCAKRNASKM

>Rattus norvegicus(P04906)

PPYTIVYFPVRGRCEATRMLLADQGQSWKEEVVTIDVWLQGSLKSTCLYGQLPKFEDGDLTLYQSNAILRHLGRSLGLYGKDQKEAALVDMVNDGVEDLRCKYGTLIYTNYENGKDDYVKALPGHLKPFETLLSQNQGGKAFIVGNQISFADYNLLDLLLVHQVLAPGCLDNFPLLSAYVARLSARPKIKAFLSSPDHLNRPI

>Wuchereria bancrofti(Q86LL8)

MSYKLTYFPIRGLAEPIRLVLVDQGIKFTDDRINASDWPSMKSHFHFGQLPCLYDGDHQIVQSGAILRHLARKHNLNGGNELETTHIDMFCEGIRDLHTKYAKMIYQAYDTEKDSYIKDILPVELAKFEKLLATRDDGKNFILGEKISYVDFVLFEELDIHQILDPHCLDKFPLLKAYHQRMEDRPGLKEYCKQRNRAKIPV

>Caenorhabditis elegans(P10299)

MTLKLTYFDIHGLAEPIRLLLADKQVAYEDHRVTYEQWADIKPKMIFGQVPCLLSGDEEIVQSGAIIRHLARLNGLNGSNETETTFIDMFYEGLRDLHTKYTTMIYRNYEDGKAPYIKDVLPGELARLEKLFHTYKNGEHYVIGDKESYADYVLFEELDIHLILTPNALDGVPALKKFHERFAERPNIKAYLNKRAAINPPVNGNGKQ

>Bos Taurus(P28801)

MPPYTIVYFPVQGRCEAMRMLLADQGQSWKEEVVAMQSWLQGPLKASCLYGQLPKFQDGDLTLYQSNAILRHLGRTLGLYGKDQQEAALVDMVNDGVEDLRCKYVSLIYTNYEAGKEDYVKALPQHLKPFETLLSQNKGGQAFIVGDQISFADYNLLDLLRIHQVLAPSCLDSFPLLSAYVARLNSRPKLKAFLASPEHMNRPINGNGKQ

>Cricetulus longicaudatus(P46424)

MPPYTIVYFPVRGRCEAMRILLADQGQSWKEEVVTVETWRKGSLKSTCLYGQLPKFEDGDLTLYQSNAILRHLGRSLGLYGKDQREAALVDMVNDGVEDLRCKYITLIYTKYEEGKDDYVKALPGHLKPFETLLSQNQGGKAFIVGDQISFVDYNLLDLLLIHQVLAPGCLDNFPLLSAYVARLSARPKIKAFLSSPDHVNRPINGNGKQ

>Mus musculus(P46425)

MPPYTIVYFPSPGRCEAMRMLLADQGQSWKEEVVTIDTWMQGLLKPTCLYGQLPKFEDGDLTLYQSNAILRHLGRSLGLYGKNQREAAQVDMVNDGVEDLRGKYGTMIYRNYENGKNDYVKALPGHLKPFETLLSQNQGGKAFIVGDQISFADYNLLDLLLIHQVLAPGCLDNFPLLSAYVARLSARPKIKAFLSSPEHVNRPINGNGKQ

>Cricetulus migratorius(P47954)

MPPYTIVYFPVRGRCEAMRILLADQGQSWKEEVITGETWGKGSLKSTCLYGQLPKFEDGDLTLYQSNAILRHLGRSLGLYGKDQREAALVDMVNDGVEDLRCKYVTLIYTKYEEGKDDYVKALPGHLKPFETLLSKNQGGKAFIVGDQISFADYNLLDLLLIHQVLAPGCLDNFPLLSAYVARLSARPKIKAFLSSPDHVNRPINGNGKQ

>Bufo bufo(P81942)

PEYTIIYFNARGRCEAMRMLMADQGAQWKEEVVTSDDWQKGDLKKAAVYGQLPGFKDGDFTLYQSNAMLRLLARNHDLYGKNPREASLIDMVNDGVEDLRLKYLKMIYQNYENGKDDYVKALPTNLGHFERLLASNNEGKGFVVGAHISFADYNLVDLLHNHLVLAPDCLSGFPLLCAYVKRISSRPKLEAYLSSDAHKKRPINGNGKQQ

>Bufo bufo(P83325)

SGYTLTYFPLRGRAEAMRLLLGDQGVSWTDDEVQMQDWAAGIRDLKKNAVFGQIPRFQEGDFVLYQSQTILRLLARYGLSGSNEREIAINEMMNDGVEDLRLKYYKFIFWDNEANKEKFLEELATQLGYFERILTNNAGKTFVLVGDKISYADYNLLDTLFCVLDLSPTCLSGFPLLSDYVERLGKRPKLQQYLKSEGRKRRPINGNGKQ

>Macaca mulatta(Q28514)

MPPYTVVYFPVRGRCAALRMLLADQGQSWKEEVVTMETWQEGSLKASCLYGQLPKFQDGDLTLYQSNTFLRHLGRTLGLYGKDQREAALVDMVNDGVEDLRCKYLSLIYTNYEAGKDDYVKALPGQLKPFETLLSQNQGGKTFIVGDQISFADYNLLDLLLIHEVLAPGCLDAFPLLSAYVARLSARPKLKAFLASPEHVNLPINGNGKQ

>Pongo abelii(Q5R8R5)

MPPYTVVYFPVRGRCAALRMLLADQGQSWKEEVVSMETWQEGSLKASCLYGQLPKFQDGDLTLYQSNTILRHLGRTLGLYGKDQREAALVDMVNDGVEDLRCKYLSLIYTNYEAGKDDYVKALPGQLKPFETLLSQNQGGKTFIVGDQISFADYNLLDLLLIHEVLAPGCLDAFPLLSAYVARLSARPKLKAFLASPEHVNLPINGNGKQ

>Mesocricetus auratus(Q60550)

MPPYTIVYFPVRGRCEAMRLLLADQGQSWKEEVVTGDSWVKGSLKSTCLYGQLPKFEDGDLILYQSNAILRHLGRSLGLYGKDQKEAALVDMANDGVEDLRCKYVTLIYTKYEEGKDDYVKALPGHLKPFETLLSQNQGGKAFIVGDQISFADYNLLDLLLIHQVLAPGCLDNFPLLSAYVARLSARPKIKAFLSSPDHVNRPINGNGKQ

>Xenopus laevis(Q8JFZ2)

MPGYVLTYFPVRGRAEPIRLLLADQGISWKEDEVQIPDWFSGKDARKKEAVFGQLPQFQDGDYVLYQSNSILRYLGNKHGLTGANDEERGHIDMVNDGVEDLRQKYGRLIFFEYETGKDKYLKELPSQLDFFERILSKNANGSKFVVGQKISFADYNLLDILQCHLDLCSKSLSAYPLLTAYVERLVARPKISEYLKSDARNKRPITPKHKK

>Capra hircus(Q9TTY8)

MASYTIVYFPVQGRCEAMRMLLADQDQSWKEEVVAMQSWLQGPLKASCLYGQLPKFQDGDLTLYQSNAILRHLGRTLGLYGKDQREAALVDMVNDGVEDLRCKYVSLIYTNYQAGKEDYVKALPQHLKPFETLLSQNKGGQAFIVGDQISFADYNLLDLLRIHQVLAPSCLDSFPLLSAYVARLNSRPKLKAFLASPEHVNRPINGNGKQ

>Macaca fascicularis(A0A023JCR5)

MPPYTVVYFPVRGRCAALRMLLADQGQSWKEEVVTMETWQEGSLKASCLYGQLPKFQDGDLTLYQSNTFLRHLGRTLGLYGKDQREAALVDMVNDGVEDLRCKYLSLIYTNYEAGKDDYVKALPGQLKPFETLLSQNQGGKTFIVGDQISFADYNLLDLLLIHEVLAPGCLDAFPLLSAYVARLSARPKLKAFLASPEHVNLPINGNGKQ

>Fukomys damarensis(A0A091DCX2)

MTSELQSGVSASLGPSPLPLWTRALEKTDLERRVPQTLKIEVRAKGAAGGGGPRNDSALGAERGGIRFIRSRGLQACTRVPPPTEVHATRCSIQAAGYAASMPPYTIVYFPVRGRGEAMRMLLTDQGQSWKEEVVTVESWTQGPLKASCLYGQLPKFQDGDLTLYQSNAILRHLGRSLGLYGKDQREAALVDMVNDGVEDLRGKYVTLIYTNYEAGKNDYVKALPGHLKPFETLLSKNQGGKAFIVGDQISFADYNLLDLLLNHQVLAPSCLDAFPLLSAYVARLSARPKLKAFLASPDHVNRPINGNGKQ

>Toxocara canis(A0A0B2VSH0)

MKPIYKLTYFDIRGLGEQIRLLLIDNKIPFEEVRIKSEDEWKGIKDSFVFGQLPCLKDDDVKIVQSGAIMRHLARRHDLYGRTEMDRTFADMFYEGIRDIQQRYIRMIYNEYEKKNEFIVDYLHDALNKLDALLESHEEGNGFILGENICFADYSLFELLDVLLILSPTCLLQCPKLKSFHQRFNERPSLQNYLMKRASANVRVNWNGKE

>Toxocara canis(A0A0B2VSM5)

MGYKVTYFAIRGLAEPIRLLLTDQSIPFEDSRIADKNEWQTMKHQFQFGQVPCLHDDDEQIVQSGAILRHLARKHNLNGANEKETTYADMFYEGIRDLHNKYTRMIYFEYETEKDNFIKDVLPVELAKFEKLLQTRGGGTGFVLGDKICFADYVLFEELDIMQILDAHALDKFPTLKAYHQRVHDRPLIKAYYKKREDAKVPVNGNGKQ

>Chlorocebus sabaeus(A0A0D9R8K4)

MWSEVRVLKHSRKSRLPAPAETVPSGNCWVFFWESFLAMKSRLNSRPPRWKTLDRVRVGPLQFQMGFSAANKVRNMALDDADAPLGTWGRRKGFPGQLRGDSGSSEAPLCGRRPGCSGRRAWGRREPAGPSRGAAGAVTQHWGGAGRDHPYKARRPQGLRWSFVVVAAAACAIMPPYTVVYFPVRGRCAALRMLLADQGQSWKEEVVTVETWQEGSLKASCLYGQLPKFQDGDLTLYQSNTFLRHLGRTLGLYGKDQREAALVDMVNDGVEDLRCKYLSLIYTNYEAGKDDYVKALPGQLKPFETLLSQNQGGKTFIVGDQISFADYNLLDLLLIHEVLAPGCLDAFPLLSAYVARLSARPKLKAFLASPEHVNLPINGNGKQ

>Heterocephalus glaber(A0A0N8ESH7)

MAPYTIVYFPVRGRCEAIRMLLADQGQSWKEEVVTMESWMQGQLRASCLYGQLPKFQDGDLTLYQSNAILRHLGRSLGLYGKDQREAALVDMVNDGVEDLRCKYVTFIYTNYEAGKNEYVKALPGHLKPFETLLSKNQGGKAFIVGDQISFADYNLLGLLLNHQVLAPGCLDAFPLLSAYVARLSARPKLKAFLASPDHVNRPINGNGKQ

>Fundulus heteroclitus(A0A146NIX1)

MGYKLTYFAIRGLAEPIRLLFTDQGITFEDSRIKDKSEWPAMKQQFQFGQVPCLYDDDEQIVQSGAILRHLARKHNLNGANESETTYADMFYEGIRDLHSKYTHMIYGAYETEKDGFIKDILPVELAKFEKLLQTRGGGAAFILGDKICFADYVLFEELDIMQILDPHALEKFPTLKAYHRRMRDRPHLKTYCQKRDESKVPVNGNGKQ

>Macaca mulatta(A0A1D5RCG2)

MGPGEGRLRAPRLGVLPRAPANLSLSAAVPPYTVVYFPVRGRCAALRMLLADQGQSWKEEVVTMETWQEGSLKASCLYGQLPKFQDGDLTLYQSNTFLRHLGRTLGLYGKDQREAALVDMVNDGVEDLRCKYLSLIYTNYISFADYNLLDLLLIHEVLAPGCLDAFPLLSAYVARLSARPKLKAFLASPEHVNLPINGNGKQ

>Dipodomys ordii(A0A1S3FJI9)

MPPYTIVYFPVRGRCEAMRMLLADQGQSWKEEVVTGEIWQKGPLKASCLYGQLPKFQDGDLTLYQSNAILRHLARTFGLYGKNQEEAALVDVVNDGVEDLRCKYVTLIYTNYEAGKNDYVKALPTHLKPFETLLSQNQGGQAFIVGDQISFADYNLLDLLLIHQVLDPGCLENFPLLTGYTKRLVARPKIKAFLDSPEHVKRPINGNGKQ

>Erinaceus europaeus(A0A1S3W606)

MPPYTIVYFPVQGRCEAMRTLLADQGQSWQEEVVTKDSWLQGPLKATCLYGQLPKFQDGDLTLYQSNAILRHLGRTLGLYGKDQREAALIDMANDGVEDLRSKYAALIYTNYEAGKEAYVKALPGHLKPFETLLSQNQGGKAFLVGDQISFADYNLLDLLRIHQTLAPDCLGAFPLLSAYLERLSTRPKIKAFLASPEHLNRPINGNGKQ

>Mesocricetus auratus(A0A1U7Q833)

MPPYTIVYFPVRGRCEAMRLLLADQGQSWKEEVVTGDSWVKGSLKSTCLYGQLPKFEDGDLILYQSNAILRHLGRSLGLYGKDQKEAALVDMANDGVEDLRCKYVTLIYTKYEEGKDDYVKALPGHLKPFETLLSQNQGGKAFIVGDQISFADYNLLDLLLIHQVLAPGCLDNFPLLSAYVARLSARPKIKAFLSSPDHVNRPINGNGKQ

>Scleropages formosus(A0A1W5AH81)

MPPYIITYFPVRGRCGAMRILMADQGVEWKEKVVVFDEWMKGDLKATCVFGQLPKFEDGDLVLYQSNAILRHLGRKHEAAGSDDKEAALIDMMNDGVEDLRLKYIKLIYQDYDTGKDQYIKDLPGHLSKFEAVLAKNKSGFLIGGKISFADYSLFEVLLNHLVLCSSCLDTFPALKSFVESMSARPGIKAFLDSDAYKKLPINGNGKQ

>Mizuhopecten yessoensis(A0A210Q711)

MELIYFPVKGRAEVIRLMLIDNGTSYTETSCANDWDSKWKPKMAFGQTPCLKDGDLTLVQSNSIIRHLARKFSLYGANEAEACRADIINDSVEDLRSAYVNLIYNNYDAGKEEYINKLPAKLQYFEKYIEGKSPYVLGDHICFADYSLFELLDIHLVLAPSCLDKFPALKALHTTVGSRQKVKAHRDSDAVKAMPINGNGKQ

>Castor canadensis(A0A250YCG2)

MPPYTIVYFPVQGRCEAMRMLLADQDQSWKEEVVMLDSWYQSPLKASCLYGQLPKFQDGDLTLYQSNAILRHLGRSFGLYGKDQREAALVDMVNDGVEDIRSKYIALIYTNYEAGKNDYVKALPTHLKPFETLLSQNKGGQAFIVGDQISFADYNLLDLLLTHKVLAPSCLDAFPLLSAYVSRLSGRPKVKAFLASPDHVNRPINANGKQ

>Onchocerca volvulus(P46427)

MSYKLTYFSIRGLAEPIRLFLVDQDIKFIDDRIAKDDFSSIKSQFQFGQLPCLYDGDQQIVQSGAILRHLARKYNLNGENEMETTYIDMFCEGVRDLHVKYTRMIYMAYETEKDPYIKSILPGELAKFEKLLATRGNGRNLILGDKISYADYALFEELDVHQILDPHCLDKFPLLKAFHQRMKDRPKLKEYCEKRDAAKVPVNGNGKQ

>Sus scrofa(P80031)

PPYTITYFPVRGRCEAMRMLLADQDQSWKEEVVTMETWPPLKPSCLFRQLPKFQDGDLTLYQSNAILRHLGRSFGLYGKDQKEAALVDMVNDGVEDLRCKYATLIYTNYEAGKEKYVKELPEHLKPFETLLSQNQGGQAFVVGSQISFADYNLLDLLRIHQVLNPSCLDAFPLLSAYVARLSARPKIKAFLASPEHVNRPINGNGKN

**Phi Class GSTs**

>Triticum aestivum(P30110)

MSPVKVFGHPMLTNVARVLLFLEEVGAEYELVPMDFVAGEHKRPQHVQLNPFAKMPGFQDGDLVLFESRAIAKYILRKYGGTAGLDLLGENSGIEELAMVDVWTEVEAQQYYPAISPVVFECIIIPFIIPGGGAAPNQTVVDESLERLRGVLGIYEARLEKSRYLAGDSITFADLNHIPFTFYFMTTPYAKVFDDYPKVKAWWEMLMARPAVQRVCKHMPTEFKLGAQY

>Populus trichocarpa(B9GQ64)

TPVKVYGPPLSTAVSRVLVTLLEKDVPFQIIPVDMSKGEHKKPDYLKIQPFGQVPAFQDESISLFESRSICRYVCEKYADRGDKGLYGTNPLERASIDQWVEAEGQSFGPSSGALVFQLAFAPRMNIPQDQGVIKQNEEKLGKVLDIYEQRLGESRFLAGDEFTFADLSHLPNGDYLVNATDKGHLFTSRENVGRWWNEISDRESWKKVIEMRKSG

>Arabidopsis thaliana(O80852)

MVLKVYGPHFASPKRALVTLIEKGVAFETIPVDLMKGEHKQPAYLALQPFGTVPAVVDGDYKIFESRAVMRYVAEKYRSQGPDLLGKTVEDRGQVEQWLDVEATTYHPPLLNLTLHIMFASVMGFPSDEKLIKESEEKLAGVLDVYEAHLSKSKYLAGDFVSLADLAHLPFTDYLVGPIGKAYMIKDRKHVSAWWDDISSRPAWKETVAKYSFPA

>Zea mays(P04907)

MAPLKLYGMPLSPNVVRVATVLNEKGLDFEIVPVDLTTGAHKQPDFLALNPFGQIPALVDGDEVLFESRAINRYIASKYASEGTDLLPATASAAKLEVWLEVESHHFHPNASPLVFQLLVRPLLGGAPDAAVVEKHAEQLAKVLDVYEAHLARNKYLAGDEFTLADANHALLPALTSARPPRPGCVAARPHVKAWWEAIAARPAFQKTVAAIPLPPPPSSSA

>Zea mays(P12653)

MAPMKLYGAVMSWNLTRCATALEEAGSDYEIVPINFATAEHKSPEHLVRNPFGQVPALQDGDLYLFESRAICKYAARKNKPELLREGNLEEAAMVDVWIEVEANQYTAALNPILFQVLISPMLGGTTDQKVVDENLEKLKKVLEVYEARLTKCKYLAGDFLSLADLNHVSVTLCLFATPYASVLDAYPHVKAWWSGLMERPSVQKVAALMKPSA

>Triticum aestivum(P30111)

MSPVKVFGHPMLTNVARVLLFLEEVGAEYELVPVDFVAGEHKRPQHVQLNPFAKMPGFQDGESLHIKSRAIAKYILRKYGGTAGLDLLGENSGIEELAMVDVWTEVEAQQYYPAISPVVFECIIIPFIIPGGGAAPNQTVVDESLERLRGVLGIYEARLEKSRYLAGDSISFADLNHIPFTFYFMTTPYAKVFDEYPKVKAWWEMLMARPAVQRVCKHMPTEFKLRARTRCLCTPRGCVPCRAGDDPTQEKRPPSRGWVIICPSTSSIPFQFQQHEESACASARASPDSLL

>Arabidopsis thaliana(P42760)

MAGIKVFGHPASTATRRVLIALHEKNVDFEFVHVELKDGEHKKEPFILRNPFGKVPAFEDGDFKIFESRAITQYIAHEFSDKGNNLLSTGKDMAIIAMGIEIESHEFDPVGSKLVWEQVLKPLYGMTTDKTVVEEEEAKLAKVLDVYEHRLGESKYLASDHFTLVDLHTIPVIQYLLGTPTKKLFDERPHVSAWVADITSRPSAQKVL

>Arabidopsis thaliana(P42761)

MVLTIYAPLFASSKRAVVTLVEKGVSFETVNVDLMKGEQRQPEYLAIQPFGKIPVLVDGDYKIFESRAIMRYIAEKYRSQGPDLLGKTIEERGQVEQWLDVEATSYHPPLLALTLNIVFAPLMGFPADEKVIKESEEKLAEVLDVYEAQLSKNEYLAGDFVSLADLAHLPFTEYLVGPIGKAHLIKDRKHVSAWWDKISSRAAWKEVSAKYSLPV

>Zea mays(P46420)

MATPAVKVYGWAISPFVSRALLALEEAGVDYELVPMSRQDGDHRRPEHLARNPFGKVPVLEDGDLTLFESRAIARHVLRKHKPELLGGGRLEQTAMVDVWLEVEAHQLSPPAIAIVVECVFAPFLGRERNQAVVDENVEKLKKVLEVYEARLATCTYLAGDFLSLADLSPFTIMHCLMATEYAALVHALPHVSAWWQGLAARPAANKVAQFMPVGAGAPKEQE

>Hyoscyamus muticus(P46423)

MGMKLHGPAMSPAVMRVIATLKEKDLDFELVPVNMQAGDHKKEPFITLNPFGQVPAFEDGDLKLFESRAITQYIAHTYADKGNQLLANDPKKMAIMSVWMEVESQKFDPVASKLTFEIVIKPMLGMVTDDAAVAENEEKLGKVLDVYESRLKDSKYLGGDSFTLADLHHAPAMNYLMGTKVKSLFDSRPHVSAWCADILARPAWSKAIEYKQ

>Silene vulgaris(Q04522)

MTIKVHGNPRSTATQRVLVALYEKHLEFEFVPIDMGAGGHKQPSYLALNPFGQVPALEDGEIKLFESRAITKYLAYTHDHQNEGTSLIHKEKHEMAAQLVWEEVEAHQFDPVASKLAWELVFKGIFGMQTDTTVVEENEAKLAKVLDVYEARLTESEYLGANDSFTLVDLHHLPLLGYLMGTQVKKLFEERAHVSAWCKKILARPSWEKTLALQKQA

>Arabidopsis thaliana(Q84TK0)

MDCLQMVFKLFPNWKREAEVKKLVAGYKVHGDPFSTNTRRVLAVLHEKRLSYEPITVKLQTGEHKTEPFLSLNPFGQVPVFEDGSVKLYESRAITQYIAYVHSSRGTQLLNLRSHETMATLTMWMEIEAHQFDPPASKLTWEQVIKPIYGLETDQTIVKENEAILEKVLNIYEKRLEESRFLACNSFTLVDLHHLPNIQYLLGTPTKKLFEKRSKVRKWVDEITSREAWKMACDQEKSWFNKPRN

>Arabidopsis thaliana(Q96324)

MVVKVYGQIKAANPQRVLLCFLEKDIEFEVIHVDLDKLEQKKPQHLLRQPFGQVPAIEDGYLKLFESRAIARYYATKYADQGTDLLGKTLEGRAIVDQWVEVENNYFYAVALPLVMNVVFKPKSGKPCDVALVEELKVKFDKVLDVYENRLATNRYLGGDEFTLADLSHMPGMRYIMNETSLSGLVTSRENLNRWWNEISARPAWKKLMELAAY

>Arabidopsis thaliana(Q9SRY6)

MGINASHVPETCYHHCNQTFESSRQCFKWCQELARKDEYKIYGYPYSTNTRRVLAVLHEKGLSYDPITVNLIAGDQKKPSFLAINPFGQVPVFLDGGLKLTESRAISEYIATVHKSRGTQLLNYKSYKTMGTQRMWMAIESFEFDPLTSTLTWEQSIKPMYGLKTDYKVVNETEAKLEKVLDIYEERLKNSSFLASNSFTMADLYHLPNIQYLMDTHTKRMFVNRPSVRRWVAEITARPAWKRACDVKAWYHKKKN

>Chondromyces apiculatus(A0A017SXI2)

MKIYGHPMSTCARKVLTVLAEKGHEAEMVLVDLMKGEQKKPEFLKLQPFGVIPVLDDDGFILYESRAIIRYLDQKLSGTSLTPSDPKERALMEQWISVETSYLSPPAMKIVAQKLFVPMRGGQTDEAIVEVGRKETVRTLDIMEQTLSKQEFLAGNSFSLADVSCMPYLGYLFPAGAGDLVTSRPGVAAWWERISSRPSWRKVAG

>Elaeis guineensis var tenera(A0A060IGG8)

MGVKVYGPTMSTCTARVLLCLEEVGAEYELVPINLSTGEHKQPAHLARNPIGQVPAFEDGALMLHESRAIARYVSRKYKSSGADLLKEGGLEESAMVDVWLEVESHQFDPAIGPIFFQSFIVPMIGGVPDQTVINTNLEKLCKVLDIYEARLSKTKYLAGDFFSLADLSHVPLLYYFMGSPHASVVNSRPHVKAWWEAVSSRPACKKVTSAMPGSA

>Theobroma cacao(A0A061DK50)

MVVKVYGPAYATPKRVLVCLIEKEVEFETVPVDLLKGEHKDPEYLKLQPFGTVPVTQDGDYILYESRAILRYYAEKYKSQGTDLLGKTVEERGLVENWLEVEAQSYHPPIYTLTVQILFSSKLGFPRDENLIKESEEKLAKVLDIYEERLSKSKYLGGDFFSLADLSHLPFTQYLVDQMGKEYMIRSRKHVSAWWDDISSRPSWQKVLQLYAAPFKN

>Theobroma cacao(A0A061DKZ7)

MVVKVYGPAYASPKRVLVCLIEKEVEFETVPVDLLKGEHKDPEYLKLQPFGTVPVTQDGDYTLYESRAIMRYYAEKYKSQGTDLLGKTVEERGLVENWLEVEAQSYNPPMFTLTVQIMLSSKLGLPRDENLIKESEEKLGKVLDIYEERLSKSKYLAGNFFSLADLSHLPFTQYLVDHMGKEYMIKSRKHVSAWWDDISSRPSWQKVLQLYAAPFKN

>Theobroma cacao(A0A061EET2)

MVVKVHGSVRAACPQRVLACLLEKDVEFEIFHVDLDAGEHKRPEFLLRQPFGQIPAVEDGDLKLFESRAIIRYYAAKYVDRGPNLLGNSLEERAVVDQWLEVEAHNFNDLVKTLVFQIVILPRMGEHGDLALAHKCEQQLEKVFDIYEQHLSKSSYLAGDSFTLADLSHLPAIRYLVNDAGMGHLVAERKHVNAWWEDISNRSAWKKLMELAKY

>Theobroma cacao(A0A061DSD1)

MKNKKVYGSLNSAATLKVLACLFEHDLDFDFVPIDLEAGEHKKKPFLSMNPFGQVPVFEDGDVKQFESRAIIRSMGHQYGKKGEELIYWDSREQAVVANWIDVEDHHFEPPALKLISELVIKPKKGLTPDEETVAEAEAKLAKVLDVYEARLSKFKYLASDKYTIADMLHLPNIQALIGTQAKKLFDSRPRLSEWCTAILARPAWIKVVEMQQKAQA

>Rhizoctonia solani(A0A066VYX0)

MVTVKLHGMPYSACAHRVWATAQEIGVTVELVPVDLAKVEHKTPEYVENYHPFGVIPVLVDEDGTKLFESRAICRYLVAKYGKGSPLLPDPSDAKAYGLFEQAASIEYSNFDPSAASLTYERIVAPMRKEKPNEELIKKCIDTLISKMDGYERILSKQKYLAGDTFTLADLFHLPYGQIISVIEPRILASKPHVKAWWDDISARESWKATLKFSGH

>Medicago truncatula(A0A072UH22)

MFSSICAESRAIMRYYAEKYRSQGVELLGKTIEEKGLVEQWLEVEAHNFHPSAYNLTCHVLCPTLLGGSSPDPKVIEESEAKLVKVFNIYEERLSKNKYLAGDFFSLADISHLPFMDYVVNNMGKDYLIKDKKHVSAWWNDISSRPSWNKVLELYKPPI

>Medicago truncatula(A0A072VNL4)

MALKIYGLAMSTNTTRAMICLHEKEVDFELIPVNVFTSEHKQPPFLNKNPFGLIPVLEDDDLTLFESRAITSYVAEKYKEVGPDLIRHNDTKEASLVKMWTEVESHYYDPAVTPIIYEYFVAPFQGQEPNKSVIESNIEKLKIVLDVYETKLSTTKYLAGDFYSLADLSHISSTHYFMQTPCASMINERIHVKAWWEDISSRTAFQKVVGGMTFGQSDQK

**Sigma Class GSTs**

>Ascaris suum(P46436)

MPQYKLTYFDIRGLGEGARLIFHQAGVKFEDNRLKREDWPALKPKTPFGQLPLLEVDGEVLAQSAAIYRYLGRQFGLAGKTPMEEAQVDSIFDQFKDFMAELRPCFRVLAGFEEGDKEKVLKEVAVPARDKHLPLLEKFLAKSGSEYMVGKSVTWADLVITDSLASWESLIPDFLSGHLQLKKYIEHVRELPNIKKWIAERPKTPY

>Homo sapiens(O60760)

PNYKLTYFNMRGRAEIIRYIFAYLDIQYEDHRIEQADWPEIKSTLPFGKIPILEVDGLTLHQSLAIARYLTKNTDLAGNTEMEQCHVDAIVDTLDDFMSCFPWAEKKQDVKEQMFNELLTYNAPHLMQDLDTYLGGREWLIGNSVTWADFYWEICSTTLLVFKPDLLDNHPRLVTLRKKVQAIPAVANWIKRRPQTKL

>Hyriopsis cumingii(A0A023I760)

MRTYRLTYSDIRGRAELARLVFVAAGESFEDRRVSREEWAELKKETPFGQIPVLEVDGKPLAQSYAIARFLGREFGLAGSSNWESAQIDQVMDLVEDLRRELLKVIFEKEPDKKKDLEQKLQDEVFPKFIVFFQKLLENTGGQYFVGSSLSLADLAVLDLFDTPLQMYPSLLDKSPALQAHRKLLESSPKLDEYLKSRKKTDI

>Danaus plexippus(A0A212FJP1)

MENVKVIYFPLKGMAEGIRLILAHNGQEFEDVRIPHEEWPAMKPNTPFGQLPILEINGKQYAQTSAIVRYLGRKYGLGGNNVDEDFEIDQNMEFFNDLRSKGSALFYEKDEKRKAALREDLQKNYYPTALAKLNDIIAQNNGHLALGRLTWADFMFAGIYDSMKYIMQIPDIDEKYPMLVELQQKVLSLPRVKEFCDRAPKSDF

>Bombyx mori(Q5CCJ4)

MPNVKFYYFPVKALGESQRLLLAYGGQEFEDNRISSENWPEFKPKTPFGQMPVLEIDGKQYAQSTAICRYLGRKYGLAGANDEEAFEIDQNVEFLNDIRASAASVHYEKDEAVKAKKKAELEETKYPFFFEKLNEILTKNNGHIALGKLTWGDFVYAGMYDYLKAMLQKPDLEQKYPAFRKPIEAVLAIPKVKAYVDAAPRTEL

>Haliotis discus discus(B6RB00)

MPSYKLIYTDNKGRAEVSRLLFALAGQEYEDVRWTRETFQTEKQNLLFGQIPVLEVDGKQYAQSMAIAGFLAREFGFHGKTSVEQMEVDQVIGIINDIFSALIKQFHEQDEEKKADIIKQNNETTFPKFISFFEEILKNNNTGFYVGDKLGFADVAAFDTLSKIEAGINMDDFPLVKANKEKVASNERVAKWLQDRPVTQF

>Haliotis discus discus(B6RB01)

MPTYKLRYFNARGFGEVSRLLFALAGQEYEDVRFTQETWPAEKPNTPLGQMPVLDVDGQSFGQSSAISRFLARRFNFYGQGDVQALQVDQVLGIIQDIINALIKAYYEKDEERKAQALKENKEEKMPLYFGMLEKLLERNGSTGFFVGNSITLADVSVFDIYDKAKPMVDLDKFPLVKKSVDNVASNPKIKTWIEKRPQTEN

>Haliotis discus discus(B6RB02)

MPTYRFRYFDYKAVGELSRLLFALAGQEYEDVRITYETWPAEKPNTPLGQVPVLEIDGKPFSQSSAISRYLARTFGFYGNGDLEALAVDQVLGVVQDVNTFMRDYHKEQDEAKKAELLKEAKDVKIPLYFGMFEKLLKKNGSTGLFVGKKISIADVSLFDICDKTTDAMLKIEDYPLVKKCCDNVAANPKIKAWVEKRPVTAF

>Aedes aegypti(Q16P79)

MPDYKVYYFNVKALGEPLRFLLSYGNLPFDDIRITREEWPALKPSMPMGQMPVLSVDGKKVHQSVAMSRYLAKQVGLAGADDWENLMIDTVVDTINDFRLKIAVVSYEPDDDVKEKKLVTLNSEVIPFYLEKLDDIARDNNGHMANGKLTWADMYFVAILDYLNYMTKSDLVANHPNLQRVVDNVTSIDSIKAWIDKRPQTEI

>Plutella xylostella(204AA)

MPVVKFYYFPIKALGEGPRLLLAYGGQEFQDIRVDKESWPEFKPKTKYGQMPILEIDGKQYAQSAAICRYLASRYGLTGADAEQNFEIDEAVDFFNDIRAKAAQVHYEEDEKVKEKRHETYSQTVYPDLLGKLHDIVQRNNGHLAANKLTWADFYFAGVYDYMKVMLRRPDLDQQYPGFAKVYETVYSLPKVKAFADAAPKTDF

>Drosophila melanogaster(P41043)

MADEAQAPPAEGAPPAEGEAPPPAEGAEGAVEGGEAAPPAEPAEPIKHSYTLFYFNVKALAEPLRYLFAYGNQEYEDVRVTRDEWPALKPTMPMGQMPVLEVDGKRVHQSISMARFLAKTVGLCGATPWEDLQIDIVVDTINDFRLKIAVVSYEPEDEIKEKKLVTLNAEVIPFYLEKLEQTVKDNDGHLALGKLTWADVYFAGITDYMNYMVKRDLLEPYPALRGVVDAVNALEPIKAWIEKRPVTEV

>Anopheles gambiae(P46428)

MPDYKVYYFNVKALGEPLRFLLSYGNLPFDDVRITREEWPALKPTMPMGQMPVLEVDGKKVHQSVAMSRYLANQVGLAGADDWENLMIDTVVDTVNDFRLKIAIVAYEPDDMVKEKKMVTLNNEVIPFYLTKLNVIAKENNGHLVLGKPTWADVYFAGILDYLNYLTKTNLLENFPNLQEVVQKVLDNENVKAYIAKRPITEV

>Papilio polytes(I4DMZ0)

MAKVKVTYFNVKALGEGIRMLLAYGGQEFEDIRVERDSWPELKPKTPFGQLPMLEIDGKQYAQSIAICRYLGRKYGLAGTTPEEDLIIDQNLDFFNDIRLKTVAANYESDEKVKADKLEDLKKNHFPVLFSKLDQMIKENNGFLAVGRLTWADFVFAGVYDALRMFTHLPDLDEKYPSFSKLRDTVRSLPKVKEFCDSAPKTDL

>Caenorhabditis elegans(Q09596)

VSYKLTYFNGRGAGEVSRQIFAYAGQQYEDNRVTQEQWPALKETCAAPFGQLPFLEVDGKKLAQSHAIARFLAREFKLNGKTAWEEAQVNSLADQYKDYSSEARPYFYAVMGFGPGDVETLKKDIFLPAFEKFYGFLVNFLKASGSGFLVGDSLTWIDLAIAQHSADLIAKGGDFSKFPELKAHAEKIQAIPQIKKWIETRPVTPF

>Nototodarus sloanii(P46088)

PKYTLHYFPLMGRAELCRFVLAAHGEEFTDRVVEMADWPNLKATMYSNAMPVLDIDGTKMSQSMCIARHLAREFGLDGKTSLEKYRVDEITETLQDIFNDVVKIKFAPEAAKEAVQQNYEKSCKRLAPFLEGLLVSNGGGDGFFVGNSMTLADLHCYVALEVPLKHTPELLKDCPKIVALRKRVAECPKIAAYLKKRPVRDF

>Danaus plexippus(A0A212FJP7)

DNVKVLYFPLKAMAEGIRLILAYVGQDFEYVRISEEEWPSVKPNTPFGQVPVIEINGKRHAQTSSILRYLGKKHGLGGNNLEEDFEIDQVVDFFNDLRLRAASLHYEKDENKKAVLKQELYNNYFPEMFTRLNDIITRNNGYMAVGKLTWADFMFAGMYERIKVMLAMPDLDEKYPKFKKLEQTVLNLPKVKEYCANEPEYN

>Manduca sexta(P46429)

MPKVVFHYFGAKGWARPTMLLAYGGQEFEDHRVEYEQWPEFKPNTPFGQMPVLEIDGKKYAQSLAISRYLGRKYGLAGNDIEEDFEIDQIVDFVNDIRASAASVEYEQDAANKEVKHEENMKNKYPFQLNKLSEIITKNNGFLALGRLTWADFVFVGMFDYLKKMLRMPDLEEQYPIFKKPIETVLSNPKLKAYLDSAPKKEF

>Fasciola hepatica(Q06A71)

MDKQHFKLWYFQFRGRAEPIRLLLTCAGVKFEDYQFTMDQWPTIKPTLPGGRVPLLDVTGPDGKLRRYQESMAIARLLARQFKMMGETDEEYYLIERIIGECEDLYREVYTIFRTPQGEKEAKIKEFKENNGPTLLKLVSESLESSGGKHVAGNRITLGDLFLFTTLTHVMETVPGFLEQKFPKLHEFHKSLPTSCSRLSEYLKKRAKTPF

>Crassostrea gigas(Q5K4L8)

MASYRLHYFDVRGRGEIVRMLFKLAQAEFGDIRVTQGEWTDVKHDTPTGELPYLEVGEKQLTQSLTIARYLAREFGLAGDTNWERALVEQVVDTCDDLRAENAKIIHERDPVRLALMKSKMKDQILPKYLNRLTKFLNEHGDRYFIGSKITSADIAVHEVLTTFLQNDPSCLDKHDVLRKHRQLVEHHPNLSEYLSSRPRFVV

>Spodoptera littoralis(A0A3G1ZLC8)

MPKYVFHYFPIKALGESVRLLLAYGGEGFEDHRIDLDDWPKFKPNTPFGQMPVIEFDGKQYAQSIAIARYLGNKYGLTGDTLEDNLEIDQNVYLINDLRIKAASAHYEKDEVIKEQKYKELSKGAFPDSLEMLNALFAKNNGHVALGKLTWADFMFAGLFDYLSAMMRMPDLGQKYPALQQVKDRVYSLPKVKAYADAVPA

>Spodoptera littoralis(A0A3G1ZLD6)

MAKKLHYFNFIAIAEPIRYILHYTKQEFEDVRHDHRFWPNAEFKEKLPFGQFPLYEEGDRMLTQSLAIAKYVARGTDLIPSDPWTQAVLDAAVYTIYDYWSKVVTFIREKDPEKKKELKRELLDETIDFFFSRLDKDLKENGGYFSGKLSWVEFVLCGLVEASNYFLDTELEKKYPRVEALIKKIKSLPGVKEYVAARGPNVFKQ

>Haliotis fulgens(A0A346QRM4)

MPTYKLRYFNARGFGEVSRLLFALAGQEYEDVRFTQETWPAEKPNTPLGQMPVLDVDGQSFGQSSAISRFLARRFNFYGQGDIQALQVDQVLGIIQDIINSMIKAFYEKDEERKAQFMKENKEEKLPLYFGMLEKLLERNGSTCFFVGNSITLADVSVFDIYDKVKSMVDLDKFPLVKKSIDNVASNPKVKTWIEKRPQTEN

>Argopecten irradians(A0A173DQE4)

MPSYKLIYFTVRGRGELIRLAFAASGQSYDEEKVTFETWPALKPKMPTKQLPVLEVDGKQLTQSLAIARYLGREFGLAGEGNMDQFLVDQVIDTGADALTAYVKWYFEKEETKKAELKKELVDTTIPKFAEILTNYLENSGGKNGFFVGSKLSLADLACHETFTDFLQLNPDCLKDYPKLAANRQKVEENANVKQYLSSRPESVI

>Ruditapes philippinarum(H6B8N8)

MATYKVSYFPWKGNGEIIRLVLVIAEQDFKDERLTMDEWLKVKEASPTKHMPLLTVNGTVYGQTAACARYLARKYGLMGSTPEEELLIDEVYECIVDFLKEVFKLTYEKDDKTKEELKQKIITENVPKLNDYIKLRSKLGGNGFIIGKKISLADIHLYNIVDQCEASFPGFFSSAPDIKKHADVVKSDARIQKWIATMPKMPQ

>Azumapecten farreri(I6LKU6)

MPSYKLIYFGVRGRGELIRLAFAASGQTFEEDTITFADWPELKQKMPTGQIPVLEIDGKQLSQSLAIARYLGREFGLAGKTNMDQCLVDQVIDTAGDCLTEYVKSHFESDETKKAELRKTLVETTIPKFAKIFTTFLENSGGKNGFFVGSELTLADLACHEAFTDFLQLNADALKDYPQLAANRQKVEENENVKRYLAKRKESPI

>Laternula elliptica(B9VX80)

MAGTVQGDKWVLYYWPGFKGRAEFVRLVFEEAGIPYLESNQGVADSIIKGEIGGYPVMMPPVVAKGDFRLGQTQMICQYLAGKYGLAPKGEEDKIHAEQVCASMYDYLTEGYGAFHGAKPGVKYADQKEEAQRYIDRVVQQRLPRYLKHFETVLAANTAGTGFLFGDSISHADLALFHIMNATEFQFPEVYKSADYIPLLKAHRDRIASRPNIVAYTQSERCKPFSGDSFM

>Spodoptera littoralis(A0A3G1ZLD1)

MPKYVFHYFDGKGLGEPVRLLLAFGDEGFEDHRVAFKDWPDFKPKTPFGQMPLLEFDGKQYAQSLSIARYLGKKYGLAGESLEDALEIDQNVDLINDLRAKAAIASYEKDEAVKEKKYAEFNKDVFPNMLEKLNEIITKNNGHIAIGKLSWGDFVFAGMFDYIKHLLVVPDLEKKYPAFQKVVDAVYSIPKVKTYADAVGPTEF

**Tau Class GSTs**

>Arabidopsis thaliana(Q9ZRW8)

MANEVILLDFWPSMFGMRTRIALREKGVEFEYREEDLRNKSPLLLQMNPIHKKIPVLIHNGKPVNESIIQVQYIDEVWSHKNPILPSDPYLRAQARFWADFIDKKLYDAQRKVWATKGEEQEAGKKDFIEILKTLESELGDKPYFSGDDFGYVDIALIGFYTWFPAYEKFANFSIESEVPKLIAWVKKCLQRESVAKSLPDPEKVTEFVSELRKKFVPE

>Populus trichocarpa(D2WL48)

MEDRVTLLDFWPSPWATRVKVALAEKGIEYESREQNLIDKSPLLLEMNPVHKTIPVLIHNGKPICESHNIVQYIDEVWKDKSPLLPSDPYQRSQARFWADYIDKKIYNNAKKLWKEKGEEQEEVKREFIEGLKTLEGELGDKLYFGGESFGFVDVVLVPVTSWFYSLEICGKFSIEAECPRFTAWIKRCMEKESVSSSLPDPHKVYDYVLLLKKKMGIE

>Populus trichocarpa(D2WL57)

MAEVKLLGAWGSPFSRRVEMALKLKGVEYEYIDEDLANKSPLLLKYNPIHKKVPVLLHNGKTMAESLVILEYIDETWKSNPILPEDPYDKAMARFWAKFIDEKCMPAIWQIMLSKENEREKAIEEAIQHLKTLENELKDKKFFGGETIGLVDIVANFIGFWLGAAQEATGMELVNKERFPVLCKWIDEYANCSVVKENLPPRDKLIAFLRPRLSASSWKY

>Glycine max(Q9FQE8)

MTDEVVLLDFWPSPFGMRVRIALAEKGIEYEYKEEDLRNKSPLLLQMNPVHKKIPVLIHNGKPISESLIAVQYIEEVWNDRNPLLPSDPYQRAQARFWADYVDIKIHDLGKKIWTSKGEEKEAAKKEFIEALKLLEEQLGDKTYFGGDNIGFVDIALVPFYTWFKVYETFGSLNIENECPRFVAWAKRCLQKESVAKSLPDQHKVYEFVVEIRKKLVIE

>Pinus tabuliformis(Q6DNI8)

MENQVKVLNLWASPFGLRVLVGLEEKGVKYEYQEENLPSKSELLLKMNPIHKKIPVLIHNDKPVLESLIIVEYIDEAWPNTNPFMPSSAYERARARFWADFVDKKIYDNGSALIMKCKGEAQEEAKRNMLEYLGLLEGALDELSGGIKPYFGGEKFGYMDIAFIPFASWFQAWEVMGNWKIPLETQFPRLHEWVNACMERESVKKVLPHPEKVAEFAMQIRQRFVGSD

>Capsella rubella(R0GRU5)

DEVILLDFWPSMFGMRTRIALEEKNVKFDYREQDLWNKSPILLEMNPVHKKIPVLIHNGKPVCESLIQVEYIDEVWPSKNPLLPSDPYQRAQAKFWGDFIDKKVYASARLIWGAKGEEQEAGKKEFIEILKTLESELGDKIYFGGETFGYVDIALIGFYSWFEAYDKFGNFSIEAECPKLIAWAKRCVERESVAKSLPDSNKIVEF

>Medicago truncatula(G7L788)

QDVKLLNFLLSPVGRRVEWALKLKGVEFDYIEEDIFNKSSLLLEMNPVHKKVPVLVHGQKSIAESLIILEYIDETWKQYPLLPPDPYQRSRARFWAKLSDEKLALGSWIALIKKGNEWEKALKEAREIMEKLEEDIKGKKFFGGDTIGYLDLTLGWITCFLPIWEEIGSTQILDPLKCPSISSWKINFLSHPIIKECLPPRDEMILYCHRRIKEY

>Glycine soja(K9M8M1)

EEVILLGKWASPFSNRVDLALKLKGVPYKYSEEDLANKSADLLRYNPVHKKVPVLVHNGNPLPESLIIVEYIDETWKNNPLLPRDPYERALARFWSKTLDDKILPAIWNACWSDENGREKAVEEALEALKILQEALKDKKFFGGESIGLVDIAANFIGYWVAILQEIAGLELLTIEKFPKLYKWSQEFINHPVIKEGLPPRDELFAFF

>Arabidopsis thaliana(Q9FUS8)

MASSDVKLIGAWASPFVMRPRIALNLKSVPYEFLQETFGSKSELLLKSNPVHKKIPVLLHADKPVSESNIIVEYIDDTWSSSGPSILPSDPYDRAMARFWAAYIDEKWFVALRGFLKAGGEEEKKAVIAQLEEGNAFLEKAFIDCSKGKPFFNGDNIGYLDIALGCFLAWLRVTELAVSYKILDEAKTPSLSKWAENFCNDPAVKPVMPETAKLAEFAKKIFPKPQA

>Arabidopsis thaliana(Q8L7C9)

MANLPILLDYWPSMFGMRARVALREKGVEFEYREEDFSNKSPLLLQSNPIHKKIPVLVHNGKPVCESLNVVQYVDEAWPEKNPFFPSDPYGRAQARFWADFVDKKFTDAQFKVWGKKGEEQEAGKKEFIEAVKILESELGDKPYFGGDSFGYVDISLITFSSWFQAYEKFGNFSIESESPKLIAWAKRCMEKESVSKSLPDSEKIVAYAAEYRKNNL

>Arabidopsis thaliana(Q9FUS6)

MAQNDTVKLIGSWSSPYSLRARVALHLKSVKYEYLDEPDVLKEKSELLLKSNPIHKKVPVLLHGDLSISESLNVVQYVDEAWPSVPSILPSDAYDRASARFWAQYIDDKCFAAVDAVVGAKDDEGKMAAVGKLMECLAILEETFQKSSKGLGFFGGETIGYLDIACSALLGPISVIEAFSGVKFLRQETTPGLIKWAERFRAHEAVKPYMPTVEEVVAFAKQKFNVQ

>Glycine max(Q9FQE7)

MASSQEEVTLLGVVGSPFLHRVQIALKLKGVEYKYLEDDLNNKSDLLLKYNPVYKMIPVLVHNEKPISESLVIVEYIDDTWKNNPILPSDPYQRALARFWAKFIDDKCVVPAWKSAFMTDEKEKEKAKEELFEALSFLENELKGKFFGGEEFGFVDIAAVLIPIIQEIAGLQLFTSEKFPKLSKWSQDFHNHPVVNEVMPPKDQLFAYFKARAQSFVAKRKN

>Glycine max(Q9FQD8)

MGSEEVKLLSFWVSPFGKRIEWALKLKGVEYEYIEEDIFNKSSLLLELNPVHKKVPVLVHAEKSIIAESFIILEYIDEKWKQYSLLPHHPYQRALARFWAATAEEMFRKVVWIALRSPTSGDEREKALKESREVMERIEEEIRGKKYFGGDNIGYLDIALGWISYWLPVLEEVGSMQIIDPLKFPATTAWMTNFLSNPVIKDNLPPRDKMLVYLKDLRSKYIVL

>Glycine max(I1MJ34)

MADEVVLLDFWPSPFGMRVRIALAEKGIKYEYKEEDLRNKSPLLLQMNPVHKKIPVLIHNGKPICESLIAVQYIEEVWNDRNPLLPSDPYQRAQARFWADYVDKKIYDLGRKIWTSKGEEKEAAKKEFIEALKLLEEQLGDKTYFGGDNLGFVDIALVPFYTWFKAYETFGTLNIESECPKFIAWAKRCLQKESVAKSLPDQQKVYEFIMDLRKKLGIE

>Capsella rubella(B2BXV3)

MAERSEDSEEVKLLGMWASPFSRRIEIALTLKGVPYEFSEQDITNKSDLLLRLNPVYKMIPVLVHNGKPISESLVILEYIDDTWRNNPILPQDPSERAMARFWAKLIDQQICVAAMKVAGTIGEERDAAVEETRNLLMFLEKELVGKDFFGGQSLGLVDIVATLVAFWLIRTEDLLGVKVVPVEKFPEIHRWVKNLSGNDVIKKCIPPEDEHLEYIRARMDKLNIKSA

>Pinus brutia(D3YLT8)

MENQVKVLNLWASPFGLRVLVGLEEKGVKYEYQEENLASKSELLLKMNPIHKKIPVLIHNDKPVLESLIIVEYIDEAWPNTNPFMPSSAYERARARFWADFVDKKIYDNGGALIMKCKGEAQEEAKRNMMEYLGLLEGALDELSGGMKPYFGGEKFGYMDIAFIPFASWFQAWEVMGNWKIPLETQFPRLHEWVNACMERESVKKVLPHPEKVAEFAMQMRQRFVGSD

>Glycine soja(I3NNW1)

MGSEEVKLLSFFASPFGKRVEWALKLKGVEYEYIEQDIFNKTSLLLQLNPVHKKVPVLVHAHKPIAESFVIVEYVDETWKQYPLLPQDPYQRALARFWANFAEQKLLDAAWIGMYSSGDEQQNAVKVAREAIEKIEEEIKGKKYFGGENIGYLDIALGWISYWLPIWEEVGSIQIIDPLKFPAITAWITNFLSHPVIKDNLPPRDKMLVYFHSRRTALSSTFQG

>Populus trichocarpa(D2X9R3)

MADVKLHGSWVSPFNYRVIWALKLKGVEFEHIVEDLTNKSELLLKYNPVYKKIPVLVHGGKPIAESLVILEYIEETWPENPLLPTDPYERAMARFWIQYGATKTAAFGALFRASGEELEKAAKEVVEVLRVLEEQGLGDKKFFGGDSINLVDISFGLFTCWLEAIEEAAGVKVLEPSTLPRLHAWAQNFIEVPLIKENIPDYDKLLLHMKGVREKMMNK

>Medicago truncatula(G7L6H8)

MAAIDEEVQLLGSVGSPFVIRVQIALKLKGIEHKYVEEKLGNLSETLLKYNPVYRMVPVLVHNGNPISESRVIIEYIDEAWKQNPILPSDPYKRALDRFWSKFIDEKCTIAAWKSVFMPDEKEREKAREELFEALQFLENELKDKFFGGEEIGFVDIAALFIPLFQEVAEKQLFPGDKFPKLQKWSLDFYNHPVVKEVMPSKEQQFGYFKARAASLAAPSK

>Pinus tabuliformis(L7S263)

MEGCGEDGQVKLLGVIHSPFVVRVRIALALKGIHYEFIEEEVLNNKSELLLQSNPVHKKIPVLIHNGKPVCESMIIVQYIEEAWGNKAFNFMPKDPYDRAIARFWAAFIDDKLTPSIWGVFNGVGEEQQKAVEESVANLLLLEEALSGKAYFGGDEIGLVDIALGGLLVGVQTIERVTGSVLIDTLKMPLLSTWAHKFCKAEEVKEVLTDPAKLFELLSEIRANLTSPPAGN

>Pinus tabuliformis(L7S255)

MATEGEKGQVKLLGATLSPFVVRVRIALALKGIDYEFIQESMHPKSELLVKSNPVHKKIPVLIHNGKPVCESMIIVQYIEEGWGNKAPNLMPEDPYDRAIARFWAAFVDDKLFPCLRGVFTGQGEQLQKAVEDSVTNFLLIEEALRTNHCFAGKAYFGGDQIGLIDIALGGLSAFIKGLEKATDSVLIDPEKMPLLSAWMDRFFKSDGVKEVMPDVTKQVEFISTRRASMISPPTSN

**Theta Class GSTs**

>Alitta succinea(A6MN06)

MSRLKLYFDLMSQPSRAVWIFLKATGIPFEEKPVALRKGEHQTEEFAKINPFQLVPVIDDGGFVLYESLSICKYLAKSRNLADHWYPSELKHRARIESYLQWHCLMVRLFASQVFRIQVIEPRAMNKPVDRQRLAKYENILSVVLDSFETVWLKDTPYICSNEISIADVACICELMQVYAVDYPLWEDRPKLEAWSKRVRERLNPHYDQANFMVDKVRNHFLASKI

>Homo sapiens(P0CG29)

MGLELFLDLVSQPSRAVYIFAKKNGIPLELRTVDLVKGQHKSKEFLQINSLGKLPTLKDGDFILTESSAILIYLSCKYQTPDHWYPSDLQARARVHEYLGWHADCIRGTFGIPLWVQVLGPLIGVQVPKEKVERNRTAMDQALQWLEDKFLGDRPFLAGQQVTLADLMALEELMQPVALGYELFEGRPRLAAWRGRVEAFLGAELCQEAHSIILSILEQAAKKTLPTPSPEAYQAMLLRIARIP

>Homo sapiens(P30711)

MGLELYLDLLSQPCRAVYIFAKKNDIPFELRIVDLIKGQHLSDAFAQVNPLKKVPALKDGDFTLTESVAILLYLTRKYKVPDYWYPQDLQARARVDEYLAWQHTTLRRSCLRALWHKVMFPVFLGEPVSPQTLAATLAELDVTLQLLEDKFLQNKAFLTGPHISLADLVAITELMHPVGAGCQVFEGRPKLATWRQRVEAAVGEDLFQEAHEVILKAKDFPPADPTIKQKLMPWVLAMIR

>Bos taurus(Q2NL00)

MGLELYLDLLSQPCRAIYIFAKKNRIPFELRTVDLRKGQHLSDAFAQVNPLQKVPILKDGDFILTESVAILLYLARKYKVPDHWYPQDLQACARVDEYLAWQHTALRRNCLRALWHKVMLPVFLGEPVSPEMLATTLAELDMALQVLEGKFLQDKAFLTGSHISLADLVAITELMHPVGAGCQVFKGRPKLAAWRQRVEAAVGEVLFQEAHEVILKAKDSQPADPTLKQKMLPKVLAMIQ

>Rattus norvegicus(Q4V8E6)

MGLELYMDLLSAPCRAVYIFARKNGIPFDFQFVDLLKGHHHSKEYIEINPLRKVPSLRDGKFILSESVAILCYLCRKYSAPSHWYPPDLHMRARVDEFMAWQHTAIQVPMSKILWIKLIIPMITGEEVPTERLDKTLDEVNKNIKQFEEKFLQDKLFITGDHISLADLVALVEMMQPMGTNHNVFISSKLAEWRMRVELAIGSGLFWEAHDRLVKLPSWDCSTLDPSIKMKICEFLQKYK

>Mus musculus(Q64471)

MVLELYLDLLSQPCRAIYIFAKKNNIPFQMHTVELRKGEHLSDAFARVNPMKRVPAMMDGGFTLCESVAILLYLAHKYKVPDHWYPQDLQARARVDEYLAWQHTGLRRSCLRALWHKVMFPVFLGEQIPPETLAATLAELDVNLQVLEDKFLQDKDFLVGPHISLADLVAITELMHPVGGGCPVFEGHPRLAAWYQRVEAAVGKDLFREAHEVILKVKDCPPADLIIKQKLMPRVLAMIQ

>Arabidopsis thaliana(Q9ZRT5)

MMKLKVYADRMSQPSRAVIIFCKVNGIQFDEVLISLAKRQQLSPEFKDINPLGKVPAIVDGRLKLFESHAILIYLSSAFPSVADHWYPNDLSKRAKIHSVLDWHHTNLRRGAAGYVLNSVLGPALGLPLNPKAAAEAEQLLTKSLSTLETFWLKGNAKFLLGSNQPSIADLSLVCELMQLQVLDDKDRLRLLSTHKKVEQWIENTKKATMPHFDETHEILFKVKEGFQKRREMGTLSKPGLQSKI

>Erythranthe guttata(A0A022Q7B5)

MELKVYADRMSQPSRAILIFCKANGLEFEEVPIQLAKKQHHSPEFAEINPMKQVPAIVHGDFKLFESHAILIYLASAFPGVADHWYPADARKRAKIHSVLDWHHSNLRRGSVGYIFNNTIALAFGLPLNPKAEAEGEKLLLASLSTIESLWLEDGPFLLGNSKPSIADLSLVCEITQLEFADEKDRERILSPHKKVLKWMEDTKKASAPYFEEIHSSMPPGIAQLKALKAQVISQLSASKH

>Macaca fascicularis(A0A023JCC0)

MGLELYLDLLSQPCRAVYIFAKKNGIPFELRIVDLIKGQHLSDAFAQVNPLKKVPALKDGDFTLTESVAILLYLTRKYKVPDYWYPQDLQARARVDEYLAWQHTTLRRSCLRALWHKVMFPVFLDEPVSPQTLAATLAELDVNLQLLEDKFLQNKAFLTGPHISLADLVAITELMHPVGAGCQVFESRPKLATWRQRVEAAVGEDLFREAHEVILKAKDFPPADPTIKQKLMPWVLAMIR

>Mus musculus(Q61133)

MGLELYLDLLSQPSRAVYIFAKKNGIPFQTRTVDILKGQHMSEQFSQVNCLNKVPVLKDGSFVLTESTAILIYLSSKYQVADHWYPADLQARAQVHEYLGWHADNIRGTFGVLLWTKVLGPLIGVQVPQEKVERNRDRMVLVLQQLEDKFLRDRAFLVGQQVTLADLMSLEELMQPVALGYNLFEGRPQLTAWRERVEAFLGAELCQEAHSTILSILGQAAKKMLPVPPPEVHASMQLRIARIP

>Rattus norvegicus(Q01579)

MVLELYLDLLSQPCRAIYIFAKKNNIPFQMHTVELRKGEHLSDAFAQVNPMKKVPAMKDGGFTLCESVAILLYLAHKYKVPDHWYPQDLQARARVDEYLAWQHTTLRRSCLRTLWHKVMFPVFLGEQIRPEMLAATLADLDVNVQVLEDQFLQDKDFLVGPHISLADVVAITELMHPVGGGCPVFEGRPRLAAWYRRVEAAVGKDLFLEAHEVILKVRDCPPADPVIKQKLMPRVLTMIQ

>Rattus norvegicus(P30713)

MGLELYLDLLSQPSRAVYIFAKKNGIPFQLRTVDLLKGQHLSEQFSQVNCLKKVPVLKDGSFVLTESTAILIYLSSKYQVADHWYPADLQARAQVHEYLGWHADNIRGTFGVLLWTKVLGPLIGVQVPEEKVERNRNSMVLALQRLEDKFLRDRAFIAGQQVTLADLMSLEELIQPVALGCNLFEGRPQLTAWRERVEAFLGAELCQEAHNPIMSVLGQAAKKTLPVPPPEAHASMMLRIARIP

>Homo sapiens(P0CG30)

MGLELFLDLVSQPSRAVYIFAKKNGIPLELRTVDLVKGQHKSKEFLQINSLGKLPTLKDGDFILTESSAILIYLSCKYQTPDHWYPSDLQARARVHEYLGWHADCIRGTFGIPLWVQVLGPLIGVQVPEEKVERNRTAMDQALQWLEDKFLGDRPFLAGQQVTLADLMALEELMQPVALGYELFEGRPRLAAWRGRVEAFLGAELCQEAHSIILSILEQAAKKTLPTPSPEAYQAMLLRIARIP

>Gallus gallus(P20135)

MGLELYLDLLSQPCRSIYIFARTNNIPFEFKHVELFKDSVLGKKPAAASGAERPRTGPSNSEGDGKISLLKKVPVLKDGDFTLAECTAILLYLSRKYNTPDHWYPSDIKKRAQVDEYLSWHHANIRANAPKTMWIKVLIPLFTGQPQPSEKLQEVMEGLSTSLKQFEERFLQDKAFIIGSEISLADLVAIVELMQPVGVGCDIFEDRPRLMEWRRRVEEAVGKELFFQAHEMILNIKELSNIQIDPQLKEHLAPVLMKMLK

>Lucilia cuprina(P42860)

MDFYYLPGSAPCRSVLMTAKALGIELNKKLLNLQAGEHLKPEFLKINPQHTIPTLVDGDFALWESRAIMVYLVEKYGKNDSLFPKCPKKRAVINQRLYFDMGTLYKSFADYYYPQIFAKAPADPELYKKMEAAFDFLNTFLEGHQYVAGDSLTVADLALLASVSTFEVAGFDFSKYANVAKWYANAKTVAPGFDENWEGCLEFKKFFN

>Homo sapiens(P0CG30)

MGLELFLDLVSQPSRAVYIFAKKNGIPLELRTVDLVKGQHKSKEFLQINSLGKLPTLKDGDFILTESSAILIYLSCKYQTPDHWYPSDLQARARVHEYLGWHADCIRGTFGIPLWVQVLGPLIGVQVPEEKVERNRTAMDQALQWLEDKFLGDRPFLAGQQVTLADLMALEELMQPVALGYELFEGRPRLAAWRGRVEAFLGAELCQEAHSIILSILEQAAKKTLPTPSPEAYQAMLLRIARIP

>Nasonia vitripennis(K7ITL4)

MSVKFYMDLMSQPSRALYIFMKTTNIPFEKKVTSLKNGENYKDGFEKISPFNKLPVIQHNGFNLTESVAIVRYLAREFNVEDHWYPKDSKAQAKVDEYLEWQHLNTRLHCASYFAVKFLWPIIKGQHIEPKTVVEHEARMIECLDQIENIWLKDNKPFLVGDRITVADLFGACEIEQPRVGGFNPREGRPVLTAWLDRVAKETAPYYEEAHSPMNKVTERNAKQSKL

>Danaus plexippus(A0A212EZ10)

MTLKLYYDLMSQPSRSLYILLKVSKCDFEPKFIDLRKGEHYSNEYAKINRFQKVPVIDHNGFVLSESVAILRYLSGENLIPPSLYPKDNQIRARVDEFLEWHHIEFRLHLSMYFRVKHMDPIITGIPPNPKTLKGYERRLISALETFESQWLKNGNEFITGNNITVADLFAASELEQPRMAGYNPAERFLRIGSWWKNVREHFSPYYDEGHVILNKIVNKQKQQSSKL

>Aedes aegypti(Q17DF6)

MPRPVKFFYDLLSTHSRALYMFFEATKIPYDPIPVCATKGEHLTDEYRECVNRFQQVPSIIDDGFKLSNGVTILKYLIREKLIPEHWYPRDSQLRAKIDEYLEWQHDNSSKVCNAFVQEKWSLLNIEDERSSEQKVEEYRRQMEQNLNQLEREWLVPGRFIIGDRITIADILAACEIEQPKIVGMDPFQGRPKLAAWLEKVRYTMTPYYQEAHQDFYKFTEKASVKN

>Aedes aegypti(Q16X19)

MANGRSIRFYYDLISQPCRALYIFLEQNKIHYQKCPIALRKCEHTTPEYLQNVNRFGKVPAIVDGKNFKLAESIAILRYLAREFTVPDHWYPRDSRRRARVDEYLEWQHSNTRLHCAGYVRYVWRGPLRGETMDPRVAKRLKAEMVGCLDFIETNVLQRDVHFIAGDEISIADLVAACEIEQPKLAGYDARVGRPKLTAWMQRVKETTQPDYDEAHKVLNKFAPTAT

>Plutella xylostella(217AA)

MSQPSRAVYILLKKSNINFEPKYVDLRKGVHYTDEYSNNINRFKKVPVIDHNGFILTESVAIIRYLGRENVLPEALFPRADKVLNTRLDEFLEWQHLGLRAPLAMYFRVVMFSPDSEKIPSYQKRMETALDEFSTLWLGRGNQYILGDTATVADLLAACEVEQPRMTGYDCTANYPVIREWMDRVRSYFNPHYAEASSIVEKIAAKRIPMKKPTAKL

>Drosophila melanogaster(E1JJS1)

MSVSFLASLLGLSNDEDQLQVAFDEVLKRRVPSRQPTNLRMSAPIRYYYDLMSQPSRALFIIFRLSNMPFEDCVVALRNGEHLTEDFKKEINRFQRVPCIHDNGYKLAESVAILRYLSAKGKIPEHLYPKYFVDQSRVDEFLEWQHMSLRLTCAMYFRTVWLEPLLTGRTPSEAKIETFRMQMERNLDVVEEVWLEGKDFLTGSSLTVADIFAACEIEQTRMADYDVRIKYPKIRAWLKRVRQSCNPYYDVAHEFVYKISGTGPQAKL

>Anopheles gambiae(Q8MUQ2)

MSRSVKLYYDLMSQPSRALYIFLSTNKIPFDRCPIALRKMQHKTDEYRRQVNRYGKVPCIVDGSFRLAESVAIYRYLCREFPTDGHWYPSDTVRQARVDEYLSWQHLNLRADVSLYFFHVWLNPLLGKEPDAGKTERLRRRLDGVLNFFDQELLSAGSGQAFLAGDRISIADLSAACEIEQAKIAGYDPCEGRPALASWLTAVRERTNPYYDEAHKYVYRLSPDHIVTPVVAEDE

>Aedes aegypti(Q5PY76)

SKLRYFYDLMSQPSRMLYIFLESTKIPYERCLVNLGKGEHLTDKFKAINRFQKVPCIVDKNDLHLAESVAIVRYLAREYPFSDHWYPKDSQKRARIDEYLEWQQHNTRAVCATYFQYVWLRPKLMGTKVNPERAEEYKQKMEDCLDFIESDYLGGGNPFLVGNEISVADLFAACEIEQPKMAGFDPCVGRPKMTAWMARVREATNPHYDEAHKLVYRIAPDS

>Drosophila melanogaster(Q7K0B6)

MSKAIKYYYDFLSQPSRALWIAMKLGKTPFEDCPVALRKQEQLTDEYRSINRFQKVPAIVDGKFQLGESVSIVRYLADKGVFSEQLYPKTLEERARVDEFLEWQHFNVRLVCSLFFRQVWLLPAKGLAPAPKPESVKKLIKDVESNLGLLERLWLEKDFLVGDKLTVADIFGSSEINQMKLCQYNVNEKQFPKVAKWMERVRDATNPYYDEAHSFVYKTSQQAVKAKN

>Drosophila melanogaster(A1Z7X7)

MSKPIRFYYDLLSPIARGLWIGLKFSNSPVEYCPIALRKFEQLTDEYKKINRFQKVPAIVGGDFHLSETIAIIRYLADKGQFDEKLYPKTLENRARVDEFLEWQHLNIRLACSMYFRDAWLFPMNGIAPKPKPEQIQALIEGVENNLGLLERLWLENDFLVGKNLTMADILGSSEINQLRLCQYRVDEKKFPKVVKWLERVRVSANPYHDEGLTFIDRKSKQSTAAKL

>Bombyx mori(B0LB14)

MVLKLYYDLMSQPSRVLYILLKTMKYDFEPKYVNLRKAEHYSEDFTKVNRMQRVPVIDHNGFILTESIAILKYLSRENVIAESLYSKESKLQARIEEFLEWQHIGLRLHCAMYFRVVHMDPILTGRKSDEKTIQGYKRRMMMALDDFDTKWLGRGTAFIVGETPTVADLVAACELEQPRMAGFEPKDHFPNIAAWWPKVRDHFAPHYEDAHVILNKIINKMDRAANSKL

>Anopheles gambiae(Q8MUQ1)

KNLKYYYDLMSQPSRALWIFLEKTKLPYEKCLINLGKGEHLTEEFKAINRFQKVPCITDSQIKLAESVAIFRYLCREYQVPDHWYPADSRRQALVDEYLEWQHHNTRATCAIYFQYVWLRPRMFGTKVDPKQAEKYRGQMEGTLDFIEREYLGSGARFIAGDEITVADLLAACEIEQPRMAGYDPCEGRPNLTQWMARVRESTNPYYDQAHKLVNKFAQDTAS

**Xi Class GSTs**

>Natrialba magadii(D3SS28)

ACPWAHRTLVTRTLKGLEDAISVSVVDPYRAEDGWQFTPEKEGCTHDHVHDVDYLRELYVRAAPDVTCRVTVPVLWDTEEDTIVNNESEEIMRMFDTEFDEFADHTVDLYPEGYQEKVDQIIDNIYEPINNGVYRAGFATEQEPYDEAVAELFGALAHWDDVLADQRYLAGDRLTEADIAMFTTLVRFDNVYHTHFMCNVQYIREFDNLWPYLRDLYQTHGIAETVEMDHITEHYYTTHPDVNP

>Haloferax volcanii(D4GT00)

GRYHLYICRACPWAHRTAMTRALKGLEDAISLSLVEPVRIDDGWEFSEDLPDPLYGEEFLRDIYLRADDEFTGRVTVPVLWDKQRETIVNNESREIMRMLDEAFDPLAERDVDLNPDGYEEEVDRLVDEIYEPVNNGVYRAGFATTQEAYEEAVEELFDALDHWDEVLDDQRFLAGDVVTEADIAMFATLIRFDHVYHTHFKCNKKAIHEYDNLWNYTKELYQLPGVAKTVNMDHIVRHY

>Halobacterium salinarum(Q9HN26)

GRYHLYVSYACPWAHRTLLVRALLGLEDAISVSVVDPVRYDQGWTFDPEKPGCTPDHVFGGTHLRDVYTEADPEYTGRVTVPVLYDTDADTIVNNESEEIMRMLDVAFDDHAARDVDLYPEGYRDEVDRLIEDIYDPINNGVYRAGFADSQRAYDNAVDDLFEALAHYDDVLAEQRYLAGDVLTEADVAMFTTLYRFDEVYHTHFKCNRKRISDYDNLWPYLRELCQLPGVADTLYMEHVKQHYYRS

>Halorhabdus utahensis(C7NPF2)

GRYHLYVCRACPWAHRTLVTRALKGLEDAITVDYVDPYRGEDGWQFTPEKDGCTPDTVNGSDYLREVYVEADPDMTGRVTVPVLWDKQEETIVNNESAEIMRMLDTEFDDVAEHDVDLYPEGYQEDIDEIIEAIYEPINNGVYKAGFADSQAAYDEAVEELFDALDHWDSVLEDQRYLAGDRLTEADIAMFTTLVRFDEVYHTHFMCNHKLIAEYDNLWPYLRDVYTTDGVAETVDIDHIKEHYYTTHPDVSP

**Zeta Class GSTs**

>Homo sapiens(O43708)

MQAGKPILYSYFRSSCSWRVRIALALKGIDYKTVPINLIKDRGQQFSKDFQALNPMKQVPTLKIDGITIHQSLAIIEYLEEMRPTPRLLPQDPKKRASVRMISDLIAGGIQPLQNLSVLKQVGEEMQLTWAQNAITCGFNALEQILQSTAGIYCVGDEVTMADLCLVPQVANAERFKVDLTPYPTISSINKRLLVLEAFQVSHPCRQPDTPTELRA

>Mus musculus(Q9WVL0)

GKPILYSYFRSSCSWRVRIALALKGIDYEIVPINLIKDGGQQFTEEFQTLNPMKQVPALKIDGITIVQSLAIMEYLEETRPIPRLLPQDPQKRAIVRMISDLIASGIQPLQNLSVLKQVGQENQMQWAQKVITSGFNALEKILQSTAGKYCVGDEVSMADVCLVPQVANAERFKVDLSPYPTISHINKELLALEVFQVSHPRRQPDTPA

>Coccidioides immitis(D2YW48)

MTTPNFELYGYFRSSCSGRLRIAFHLKSIPYTRHPVNLLKGEQHSDTYKSLNPTNTVPLLVVSNINNTVSPSSASFSIGQSLAALEYLEEALPTNARPLLPPISNPVARAHVRTICNIIACDVQPVTNLKIQKKVKALDGDPTVWSRDLATQGFGAVEKLLELSAGRFCVGDEITLADVCLVPAVWAAERVGMDLARFPITKRVFEEMLKEEAVQKAHWQKQEDTPEDLRA

>Dianthus caryophyllus(P28342)

MSSSETQKMQLYSFSLSSCAWRVRIALHLKGLDFEYKAVDLFKGEHLTPEFLKLNPLGYVPVLVHGDIVIADSLAIIMYLEEKFPENPLLPQDLQKRALNYQAANIVTSNIQPLQNLAVLNYIEEKLGSDEKLSWAKHHIKKGFSALEKLLKGHAGKYATGDEVGLADLFLAPQIIASITGFGMDMAEFPLLKSLNDAYLKYQHFRMRCQRISPMLDEAKS

>Euphorbia esula(P57108)

MASVEQPNKPKLKLYSYFRSSCSFRVRIALNLKGLDYEYVPVNLLKGEQFTPEFLKINPIGYVPALVDGEDVISDSFAILMYLEEKYPEHPILPADIHKKAINYQAANIVSSSIQPLQNLAVLNFIGEKVSPDEKVPWVQRHISKGFAALEKLLQGHAGRFATGDEVYLADLFLEPQIHAAITRFNVDMTQFPLLLRLHEAYSQLPEFQNAMPDKQPDSTSPTAS

>Dianthus caryophyllus(Q03425)

MSSSETQKMQLYSYSSSSCAWRVRIALHLKGLDFEYKAVDLLKGEHLTPEFLKLNPLGYVPALVHGDIVIADSLAIIMYLEEKFPENPLLPRDLQKRALNYQAANIVASNIQPFQNLAVLNYIEEKLGSDEKLSWANHHIKKGFS

>Aedes albopictus(A0A023EJH2)

YWRSSCSWRVRIALNLKEIPYDIKPISLIKSGGEQHCNEYREVNPMEQVPALQIDGHTLVESLAIMHYLEETRPQRPLLPQDVLKRAKVREICEVIASGVQPLQNLIVLIHVGEEKKKEWAQHWITRGFRAIEKLLSTSAGKFCVGDEITLADCCLVPQVFNARRFHVDLRPYPIILRIDRELEGHPAFRAAHPSNQPDCPPEAAK

>Aedes albopictus(A0A023EMR1)

SACQTAIATSATHPPSSGLHLFLSSRWNDTAAKINYSKFHNAMSLSAMSKPILYSYWRSSCSWRVRIALNLKEIPYDIKPISLIKSGGEQHCNEYREVNPMEQVPALQIDGHTLVESLAIMHYLEETRPQRPLLPQDVLKRAKVREICEVIASGVQPLQNLIVLIHVGEEKKKEWAQHWITRGFRAIEKLLSTSAGKFCVGDEITLADCCLVPQVFNARRFHVDLRPYPII

>Triatoma infestans(A0A023FAZ9)

MSLIGKPVLYSYWRSSCSWRVRIALNLKEIPYDIKPISLIKGGGEQHSNEFREINPMEHVPALQIDGHTLIESLSIMYYLEETRPQRPLLPQDVYKRAKVREICDVIASGIQPLQNIGVLIYVGEEKKKEWAQHWITRGFRAVEKLLSSSAGKYSVGDELTLADCCLIPQVFNARRFQVDLRPYPIILRIDRELENHPAFRAAHPSNQPDCPPEATK

>Amblyomma cajennense(A0A023FJC9)

MSKPVLLSNYLSSCAWRVRIVLEVKKIPYEYRTVNLKPVDGEQQTDKFKALNPMGQVPVLLVDGKSISQSVAIMEYLEEKYPEPRLLPADAYLRAKCREVVELLVSGIQPLQSIGLIPLLGKAEWKKWADRTITRGFTALETIFAETAGKYCFGDEVTFADACLVPQVCNAYRFGVDVTPXXTIRRIYDALQQHPLVKKADPSCQPDAHLH

>Amblyomma parvum(A0A023FXL6)

MSKPVLLSNYLSSCAWRVRIVLEAKKIPYEYRTVDLKPANGEQQTDKFKALNPMGQVPVLLVDGKPISQSVAIMEYLEEKYPEPRLLPADSYLRAKCREVVELLVSGIQPLQSIGLIPLLGKAEWKKWADRTISRGFTALEAIFAETAGKYCFGDELTFADACLVPQVCNAYRFGVDVTPXPTIRRIYDALQQHPLVKKADPSCQPDTPPTGIPNT

>Amblyomma triste(A0A023GJ13)

MSKPVLLSNFLSSCAWRVRIVLEVKKISYEYRTVDLKPKDGEQQTDKFKALNPMGQVPVLLVDGKPISQSVAIMEYLEEKYPQPSLLPTDLYFRAKCREVVEVLVSGIQPLQSIGLIPLLGKAEWKKWADRNITRGFTALEAILTETAGKYCFGDEVTFADACLVPQVCNAYRFGVDVTPFPTIRRIYEALQQHPLVKKADPSCQPDTPPTGIPNTTDLFKGPGDQ

>Escherichia coli(A0A023Z6H5)

MKLYSFFNSSASYRVRIALALKGINYQTEGVNIRIGQQNELAYRRMNPVGLVPTLLTDEGQSLGQSLAIIDWLERHYPQVPLVPQEEPARNKVLEIVYAIACDIHPLNNLRVLRYLTEELNVSEEEKKRWYAHWIQQGLSAVEQLLRQNQSGQFCVGETPTLADCCLVPQWANALRMNCDLSGYPRCKAVYDACTQLPAFIAAAPENQQDKISA

>Serpentinomonas raichei(A0A060NLQ4)

MKLYNYFRSSTSFRVRIALNLKGLDCEYASVHLARGEQREAAYRALSPDGLVPLLDLQGVPEPGLLSQSMAIIEYLDEVYPQPPLLPPDPLGRSRVRALAQSVACEMHPINNLRVLKYLAQPLGLNEEQRAAWYNHWVVQGLLAYEQRLRELQAERAARALPPARYSYGDAPSLADCCLVPQLINGRRFGLSYDALDIPLTLAVLQACLELDAFRRALPENCPDAPPV

>Theobroma cacao(A0A061DU28)

MAPKDGGEASSKLVLYSYWQSSCSWRVRFALNLKGLSYEYKAVNLAKGEQFTPEFEKLNPLHFVPVLVDGDVVVSDSYAILMYLEEKYPQRTLLPADPQQKALNLQVASIISSSIQPLLMLSILKYLEEKVGLEERLLFVQTNIEKGFLALEKLLKDFVGKYATGEEVYMADVFMAPQIAVATERFKIDMSKFPTLSRIYESQRALPEFLAASPERQPDAVH

>Theobroma cacao(A0A061DV17)

MAPKDGGEASSKLVLYSYWQSSCSWRVRFALNLKGLSYEYKAVNLAKGEQFTPEFEKLNPLHFVPVLVDGDVVVSDSYAILMYLEEKYPQRTLLPADPQQKALNLQKYLEEKVGLEERLLFVQTNIEKGFLALEKLLKDFVGKYATGEEVYMADVFMAPQIAVATERFKIDMSKFPTLSRIYESQRALPEFLAASPERQPDAVH

>Theobroma cacao(A0A061DVZ4)

MAPKDGGEASSKLVLYSYWQSSCSWRVRFALNLKGLSYEYKAVNLAKGEQFTPEFEKLNPLHFVPVLVDGDVVVSDSYAILMYLEEKYPQRTLLPADPQQKALNLQVASIISSSIQPLLMLSILKYLEEKVGLEERLLFVQTNIEKGFLALEKLLKDFVGKYATGEEVYMGFVICRLMYLWHLRLLWLQNGLRLTCPSSLL

>Panstrongylus megistus(A0A069DPN4)

MSLIGKPVLYSYWRSSCSWRVRIALNLKEIPYDIKPISLIKGGGEQHSNEFREINPMEHVPALQIDGHTLIESLSIMYYLEETRPQRPLLPQDVYKRAKVREICDVIASGIQPLQNIGVLIYVGEEKKKEWAQHWITRGFRAVEKLLSSSAGKYSVGDELTLADCCLIPQVFNARRFQVDLRPYPIILRIDRELENHPAFRAAHPSNQPDCPPEATK

>Erythrobacter litoralis(A0A074MD55)

MKLHGYYRSSTSYRLRIALELKGLAFENVPVNLLESAQKDAAFTSRNPFASVPMLEADGRDRAQSMALIEWLDEAYPAKPLLPADIEARYTARELAYAIATELHAPLNLPVLKYLKEEYGKSPGEIGEWYRHWLARTLVPVEQRLEQLGTGDFLFDAPGLFETVLVPQLYNARRFEYDLSASPRMTRIEAACLALEPFRRAHPDNQNDNPQRETS

>Erythrobacter longus(A0A074N282)

MKLYGYFRSSTSYRLRIALELKGLAYENIGVNLLHSEQKDEGFTSRNPFGSVPLLEADGRDRAQSMAMIEWLDEAYPQKPLLPSDIEDRYTARELTYAIATELHAPLNLPVLKYLKNEYGKSQDEIDIWYRHWLARTLVPLEQRLAQLGTGDFLFDKPGIFEVVLMPQIYNARRFAYDFGDAPHMMRIEAACLALPEFQRAHPDNQNDNPERT

>Spodoptera litura(A0A075X262)

MGDDVETKPVLYSYWRSSCSWRVRIALNLKEIPYDIKAVSLIKGGGEQHCNEYREVNPMEQVPSLCIDGHTLVESLNIMHYLEETRPNQRPLMPQDCFKRAKVREICEVISSGIQPLQNLIVLIYVGEEKKKEWAQHWITRGFRAVEKLLSACAGKYCVGDEITLADCCLVPQVFNARRFHVDLRPFPIILRIDRELENHPAFRAAHPSSQPDCPPEVAK

>Spodoptera litura(A0A075X2K1)

MAENRAILYAYWLSSCSWRVRAALHFKGIPFEERPIDIVKTNQQKTEQFRAINPAQKVPALVIDNVTLVESMAIVQYLEDTHPEPTLTPKTPVLRARMRELCEVVVSGIQPLQNIGLRSQFDTTEQYTKFTKYWTDRGLMTLEDLLQKSAGKYCVGDQLTLADLCLVPQLYNAVTRHALDISKYPTVSKLYESLLKENVFKETHPESVKSKM

>Triticum aestivum(O04437)

MATAKPILYGAWISSCSHRVRIALNLKGVDYEYKAVNPRTDPDYEKINPIKYIPALVDGDFVLSDSLAIMLYLEDKYPQHPLVPKDIKTKGLDLQIANIVCSSIQPLQGYGVIGLHEGRLSPDESLEVVQRYIDKGFRAIEKLLDGCDSKYCVGDEVHLGDVCLAPQIHAAINRFQIDMTKYPILSRLHDAYMKIPAFQAALPQNQPDAPSAK

>Nasonia vitripennis(K7JNH5)

MSVIGKPILYSYWRSSCSWRVRIALNLKEIPYDIKPISLVKNGGEQHSNEFREINPMEQVPALHIDNHTLIESLNILLYLEETRPHRPLMPVDPVKRARVREICEVIASGIQPLQNLIVLIYVGEERKKEWAQHWITRGLKAVEKLLSASAGKYCVGDEITLADCCLVPQIFNARRFHVDLRPFPTILRVDRHLENHPAFTAAHPNNQPDCPPEATK

>Apis mellifera(A0A088AB84)

MSVMGKPILYSYWRSSCSWRVRIALNLKEIPYDIKPVSLIKGGGEQHSNEFREINPMEQVPALHIDNHTLIESLNILQYLEETRPHRPLMPADPVKRARVREICEVIASGIQPLQNLVVLIYVGEERKKEWAQHWITRGLTAVEKLLSSSAGKYCVGDEITLADCCLIPQIFNARRFLVDLRPFPTILRVDRHLENHPAFTAAHPNNQPDCPPEATK

>Plutella xylostella(214AA)

MAKPVLYSYWRSSCSWRVRIALNLKEIPYDIKAVSLIKGGGEQHCNEYRECEVISSGIQPLQNLVVLIYVGEEKKKEWAAHWMTRGFRAVERLLSGSAGKYCVGDEITLADCCLVPQVFNARRFHVDLRPFPIILRIDRELEHHPAFRAAHPSTQPDCPPEAAK

>Plutella xylostella(213AA)

MASPAVLHGFFASSCTWRVRAALVLKSIPFEERHVDIVQLKTHLSDQYQAVHPAQKVPALEIDGTTLVESMAILQYLEDTRPRPALAPAAPLPRARMREIVETIVSGIQPLQNVGVRGLLGSDEEYSAFSRGAARRALQTLEALLARSAGQYCVGDQLSMADLCFVPQLFNAVGRLKLDISDLPTISKLYAKLSKEEIFMKTHPRTVKHLSET

>Drosophila melanogaster(Q9VHD2)

MSTNLCPNASSSDIQPILYSYWRSSCSWRVRIAMNLKEIPYDIKPISLIKSGGEQHCNEYREVNPMEQVPALQIDGHTLIESVAIMHYLEETRPQRPLLPQDVHKRAKVREIVEIICSGIQPLQNLIVLIHVGEEKKKEWAQHWITRGFRAVEKALSTSAGKYCVGDEISMADCCLVPQVFNARRFHVDLRPYPIILRIDRELESNPAFRAAHPSNQPDCPPELPNK

>Bombyx mori(Q2I0J4)

MGKQPVLYSYWRSSCSWRVRIALNLKEIPYDIKAVSLIKGGGEQHCNEYREVNPMEQVPSLCIDGHTLIESLNIMHYLEETRPQRPLMPQDCFKRAKVREICEMIASGIQPLQNLIVLIYVGEEKKKEWSQHWITRGFRAIEKLLSTTAGKYCVGDEITLADCCLVPQVFNARRFHVDLRPFPIILRIDRELENHPAFRAAHPSSQPDCPPEVAK

>Bombyx mori(A5HSJ9)

MVENRVILHAYWLSSCSWRVRAMLHAKSIPFEERPVDIVKTGKQLTEEYRAINPAQKVPALEIDGVTLVESTAIIQYIEDTRPEPKLMPDTALQRARMREICETIVSGIQPLQNFGLKKHLGTEEKFLSFTKYWTERGLQTLNDLLAKTSGAYCIGDQITLADICLVPQIYNGVSRHKLDLKTYPIVSKVYENLLKEELYQATHPKATKEKLKINL

>Danaus plexippus(A0A212FF03)

MAETRAILYGFWASSCTWRVRAALHFKGIAFEEKSVDIVTEQKQLTDEYRYINPSQKVPALVMNGETIVESMAIIQYVEEIKPEASLVPTTPILRARMREICETIVSGIQPLQNIGLKRRFDSEHKFKEFAEYFTSRGLESVEELLKKTAGRFCIGDQITVADLCLVPQVYNGIVRYKMNMEKFPIVSSVYEHLLKEKTFIETHPKNIK

>Arabidopsis thaliana(Q9ZVQ4)

AKLKLYSYWRSSCAHRVRIALTLKGLDYEYIPVNLLKGDQSDSDFKKINPMGTVPALVDGDVVINDSFAIIMYLDDKYPEPPLLPSDYHKRAVNYQATSIVMSGIQPHQNMALFRYLEDKINAEEKTAWITNAITKGFTALEKLLVSCAGKYATGDEVYLADLFLAPQIHAAFNRFHINMEPFPTLARFYESYNELPAFQNAVPEKQPDTPS

>Arabidopsis thaliana(Q9ZVQ3)

EKLKLYSYWRSSCAHRVRIALALKGLDYEYIPVNLLKGDQFDSDFKKINPMGTVPALVDGDVVINDSFAIIMYLDEKYPEPPLLPRDLHKRAVNYQAMSIVLSGIQPHQNLAVIRYIEEKINVEEKTAWVNNAITKGFTALEKLLVNCAGKHATGDEIYLADLFLAPQIHGAINRFQINMEPYPTLAKCYESYNELPAFQNALPEKQPDAPS

**Out Group**

>Oryctolagus cuniculus(P08628)

MVKQIESKSAFQEVLDSAGDKLVVVDFSATWCGPCKMIKPFFHALSEKFNNVVFIEVDVDDCKDIAAECEVKCMPTFQFFKKGQKVGEFSGANKEKLEATINELL
