# Supplementary material for: Comparative analyses and structural insights of new class glutathione transferases in Cryptosporidium species
Source: Sci Rep. 2020 Nov 23;10:20370. doi: 10.1038/s41598-020-77233-5 (PMC7683740; doi:10.1038/s41598-020-77233-5)
Supplement: Supplementary file 3 — Supplementary Dataset 2. [file 41598_2020_77233_MOESM3_ESM.pdf]

Comparative Analyses and Structural Insights of New Class Glutathione Transferases in *Cryptosporidium* species

Mbalenhle Sizamile Mfeka [1], José Martínez-Oyanedel [2], Wanping Chen [3], Ikechukwu Achilonu [4], Khajamohiddin Syed [5]*, Thandeka Khoza [1]*

[1] Department of Biochemistry, School of Life Sciences, University of KwaZulu-Natal (Pietermaritzburg campus), Scottsville, 3209, KwaZulu-Natal, South Africa.

[2] Laboratorio de Biofísica Molecular, Departamento de Bioquímica y Biología Molecular, Facultad de Ciencias Biológicas, Universidad de Concepción, Barrio Universitario S/N, Casilla 160_C, Concepción, Chile.

[3] Department of Molecular Microbiology and Genetics, University of Göttingen, Göttingen 37077, Germany; chenwanping1@foxmail.com.

[4] Protein Structure-Function Research Unit, School of Molecular and Cell Biology, University of the Witwatersrand, Braamfontein, Johannesburg, South Africa.

[5] Department of Biochemistry and Microbiology, Faculty of Science and Agriculture, University of Zululand, 1 Main Road Vulindlea, KwaDlangezwa 3886, South Africa.

* Corresponding authors' email:

khajamohiddinsyed@gmail.com and khozat1@ukzn.ac.za

Supplementary Dataset 2. A high-resolution phylogenetic tree of the glutathione transferases (GSTs) protein sequences of *Cryptosporidium* species with GSTs from 17 different GST classes. Thioredoxin from *Oryctolagus cuniculus* (protein ID: P08628) is used as an outgroup. Three new GST classes reported in this study from *Cryptosporidium* species named Vega, Gamma and Psi are also shown in the tree.

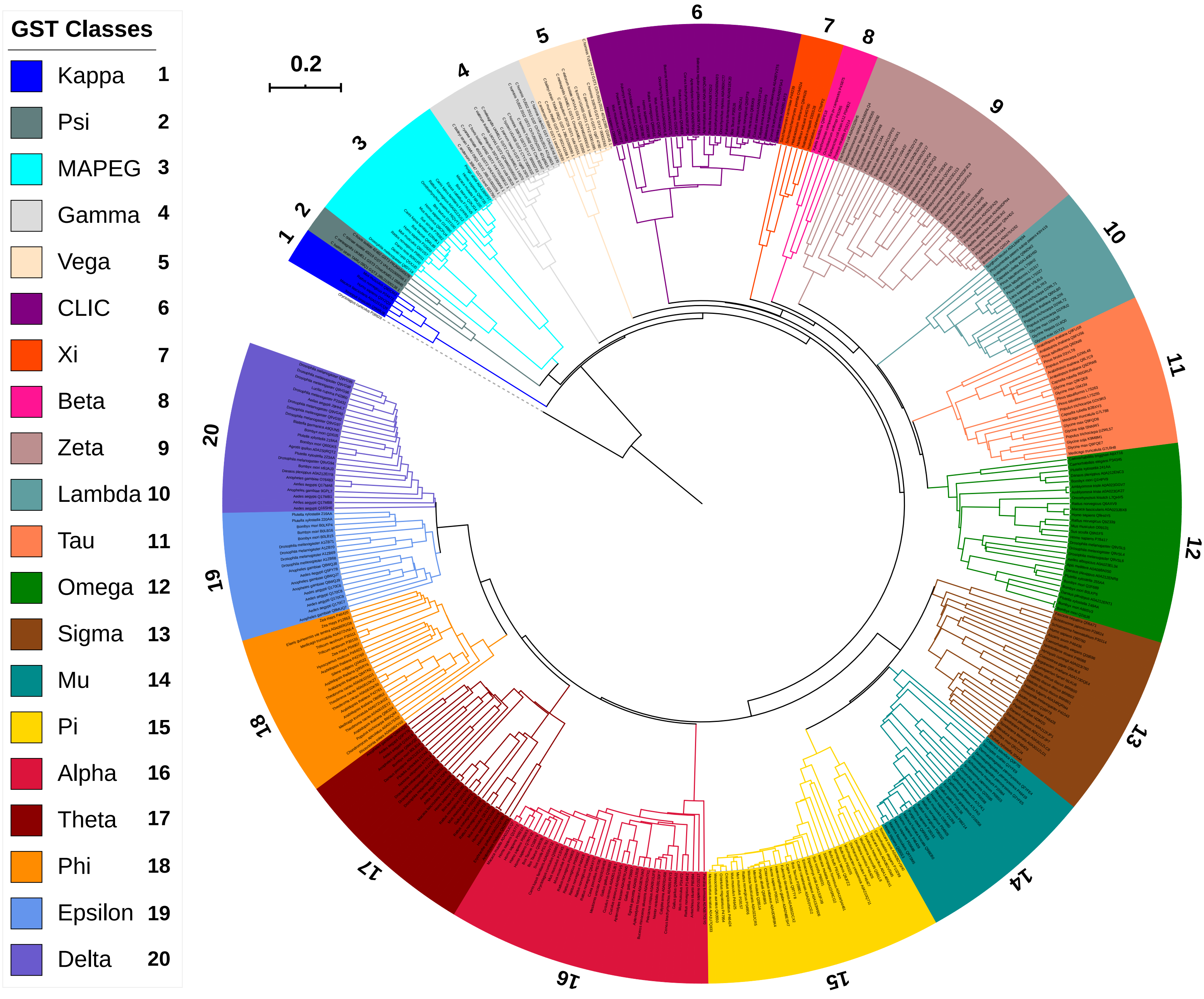
GST Classes
Kappa 1
Psi 2
MAPEG 3
Gamma 4
Vega 5
CLIC 6
Xi 7
Beta 8
Zeta 9
Lambda 10
Tau 11
Omega 12
Sigma 13
Mu 14
Pi 15
Alpha 16
Theta 17
Phi 18
Epsilon 19
Delta 20
0.2
1
2
3
4
5
6
7
8
9
10
11
12
13
14
15
16
17
18
19
20
